# Differences in genetic diversity between early isolates of yellow fever virus genotypes

**DOI:** 10.1080/22221751.2025.2601371

**Published:** 2025-12-15

**Authors:** Madison E. Lee, Clairissa A. Hansen, Jill K. Thompson, Haiping Hao, Nigel Bourne, Alan D.T. Barrett

**Affiliations:** a Department of Pathology, University of Texas Medical Branch, Galveston, TX, United States; b Department of Biochemistry and Molecular Biology, University of Texas Medical Branch, Galveston, TX, United States; c Sealy Institute for Vaccine Sciences, University of Texas Medical Branch, Galveston, TX, United States; d Division of Vaccinology, Department of Pediatrics, University of Texas Medical Branch, Galveston, TX, United States

**Keywords:** Yellow fever virus, genotype, genetic diversity, next-generation sequencing, single-nucleotide variants, orthoflavivirus

## Abstract

Yellow fever virus (YFV) is the prototypical member of the genus *Orthoflavivirus* and is responsible for the disease yellow fever. Phylogenetic studies show YFV can be characterized into seven genotypes (East Africa [EA], East and Central Africa [ECA], West Africa I [WAI], West Africa II [WAII], South America I [SAI], South America II [SAII], and Angola [ANG]). We used Next Generation Sequencing to investigate and compare the genetic diversity of the seven genotypes of YFV using early isolated strains of known passage history from a variety of collection years. Genetic diversity varied between the seven genotypes, with the ECA genotype being more diverse than EA and WAI genotypes, but not the WAII genotype. Genotype-specific amino acids (GSaas) were identified that occurred in all viral proteins but varied by genotype, with most being found in the non-structural proteins, which make up the viral replication complex. Interestingly, the fewest GSaas were found in the EA and ECA genotypes despite the ECA genotype having greater genetic diversity than either the EA or WAI genotypes. Overall, based on examination of early strains, we show that genetic diversity and GSaas varied between the seven genotypes of YFV and are not random but likely caused by selection pressure(s) such as different primate and mosquito hosts in different geographic areas. Examination of genetic diversity and GSaas in recent strains will enable genetic diversity to be investigated over time, and those involved in outbreaks may enable a better understanding of how outbreaks develop and help identify interventions to their spread.

## Introduction

Yellow fever virus (YFV) is a well-characterized representative of the genus *Orthoflavivirus*, family *Flaviviridae*, and is the cause of the disease yellow fever. YFV is a mosquito-borne virus that is transmitted via *Haemagogus, Sabethes*, and *Aedes* mosquitoes to non-human primates (NHPs) and humans. Yellow fever can range in severity from a mild febrile illness to a severe disease with haemorrhage and substantial liver damage that can be fatal [1]. Currently, YFV remains endemic across tropical regions of sub-Saharan Africa and South America, encompassing 47 countries. Large epidemics of YFV canonically occur when infected individuals travel to areas with a high population density, high host mosquito density, and poor vaccination rates.

The genome of YFV is an 11 kb single-stranded, positive-sense RNA molecule that has a single open reading frame (ORF). This is translated as a single polyprotein, and undergoes co- and post-translational processing to yield three structural proteins (envelope [E], membrane [M], and capsid [C]) and seven nonstructural (NS) proteins (NS1, NS2A, NS2B, NS3, NS4A, NS4B, and NS5), which form the viral replication complex [2]. Based on genomic sequences, YFV can be classified into seven genotypes (East Africa [EA], East and Central Africa [ECA], West Africa I [WAI], West Africa II [WAII], South America I [SAI], South America II [SAII], and Angola [ANG]) [3]. Phylogenetic studies indicate YFV originated in East Africa and subsequently spread across the African continent before entering the Western Hemisphere via the slave trade in the 1600s [4–8].

The current strategy to control YF is prophylactic use of the live-attenuated vaccine (LAV), strain 17D, derived from the wild-type (WT) strain Asibi by 176 passages in mouse and chicken tissue. Strain 17D is no longer available, but three vaccine substrains are used today: 17D-204, 17DD and 17D-213. There are only 20 common amino acid substitutions between Asibi and the three 17D substrains, although the exact mechanism of attenuation has not been fully defined [6]. Next Generation Sequencing (NGS) studies have shown that 17D LAV strains have very low genetic diversity compared to the WT Asibi strain [6]. Interestingly, the same phenomenon is seen in another YFV parent/vaccine pairing: WT French Viscerotropic virus (FVV) and its LAV derivative French Neurotropic vaccine (FNV) [9,10].

With the exception of the WT YFV Asibi and FVV strains (both members of genotype WAII), few studies have utilized NGS to examine the genetic diversity of YFV strain populations. The studies that have been published generally analyse viral RNA directly from serum samples for diagnostics and to determine consensus genomic sequences [9,11,12]. The first such study conducted NGS of serum from a 16-year-old male in Uganda but had an average coverage of only 92 reads per nucleotide [12]. A more recent study analysed an isolate (CNYF01/2016 of the ANG genotype) collected from an individual who returned to China from Angola [11]. The only NGS study with high-depth sequence coverage investigated genetic variation in an individual infected with an ANG genotype strain during the 2016 YFV outbreak in Angola, using samples collected at multiple time-points during infection [13]. However, to the best of our knowledge, NGS has never been used to examine the genetic diversity of all seven WT genotypes of YFV and obtain a better understanding of how outbreaks develop. To address this gap in knowledge, we examined the genetic diversity of representatives of all seven genotypes, with the inclusion of 21 strains of known passage history, ranging in collection years, plus examples of different passage histories of some strains. Interestingly, the ECA genotype viruses showed the greatest genetic diversity, yet the fewest genotype-specific amino acids were found in the EA and ECA genotypes.

## Methods

### Viruses

A panel of 21 WT YFV virus strains was selected that have been described and used in our previous publications, and have known passage histories [5,7,8,10,14,15]. An additional eight YFV stocks were added to examine the implication of passage history on our dataset; among these other stocks are isolates of wild-type strain FVV from different laboratories, [5, 9, 10,16].

### RNA extraction and sequencing

RNA was extracted from WT YFV strains using the Qiagen Viral RNA Mini Kit and sent to the University of Texas Medical Branch Next-Generation Sequencing (UTMB NGS) Core for genome-wide sequencing as described previously [9]. Briefly, random hexamer primers were used to construct an NGS library using the NEBNext Ultra II RNA kit via RT–PCR.

Complementary DNA (cDNA) libraries were constructed, and samples were sequenced on the Illumina NextSeq550 platform, resulting in the generation of consensus sequences and BAM files. The BAM files were converted to FASTQ for each strain were assembled to complete genome using Abyss 2.3.7 using a variety of kmer sizes and read depth. FASTQs were further re-aligned to the assembled consensus sequence using Bowtie 2. The files were then sorted using SAMtools, and duplicate reads were removed with Picard-tools. To ensure uniform coverage, the data sets were downsampled to 723 reads/nucleotide. Single-nucleotide variant (SNV) frequencies were computed using RStudio, while Shannon entropy (SE) values were calculated manually in Microsoft Excel to examine the levels of genetic diversity exhibited by each sample. The WT Asibi strain, which has been extensively studied, was included as a control to ensure the validity of sequencing data. Statistical analyses were performed using RStudio, Microsoft Excel (for example, ANOVA tests were performed with a threshold of *p* < 0.05), and Geneious software (version 2025 1.2).

## Genotype consensus sequence generation

Individual viral consensus sequences were aligned according to genotype, and a consensus sequence for each genotype alignment was created using the Geneious software program. The genotype-level consensus sequences were aligned and then exported into Microsoft Excel to assess amino acid variation across the seven genotypes. Following the identification of GSaas, amino acid changes-to-protein-length ratios were calculated to determine if there was a correlation between protein length and amino acid changes.

### Phylogenetic analysis

Using the amino acid consensus sequences generated, a phylogenetic tree was constructed using Clustal with the New York 99 strain of West Nile virus (WNV) serving as an outgroup (Genbank HQ59519.1).

## Results

### Viral strains included in the analysis and comparison of consensus sequences

Twenty-one WT YFV strains collected from different geographic locations and in different years that represented all seven YFV genotypes were selected for this study (Table 1). The passage history of all the strains was known, and they were the earliest isolates of each genotype that were available to the authors. In addition, we included eight virus preparations with different passage histories to explore the impact of passage history on genetic diversity within the same strain (Supplementary Table 1).

**Table 1.** Nucleotide length of each viral strain included in the study. The polyprotein and 5’ NCR remained consistent (10236 nucleotides and 117 nucleotides, respectively) while the 3’ NCR varied in length due to repeat sequences. Information on the strains can be found in references [5,7,8,10,14,15].

Genotype	Strain	Taxon	#nt in polyprotein	#nt in 5’ NCR	#nt in 3’ NCR
Angola	14FA	Angola/1971	10236	117	468
South America I	1337	Ecuador/1979	10236	117	651
	P128MC	Venezuela/1959	10236	117	651
	GML902621	Trinidad/1954	10236	117	675
	BeAR 512943	Brazil/1992a	10236	117	651
	CAREC 890692	Trinidad/1989a	10236	117	603
South America II	OBS 7549	Bolivia/1999b	10236	117	432
	149	Peru/1995d	10236	117	441
	HEB 4236	Peru/1995c	10236	117	438
East Africa	BC7914	Kenya/1993	10236	117	465
	MR896	Uganda/1948b	10236	117	432
	A709-4-A2	Uganda/1948a	10236	117	528
East and Central Africa	ArB8883	Central African Republic/1977a	10236	117	426
	STA-LSF-4-4143	DRC/1958	10236	117	465
	Z19039	Uganda/1972	10236	117	465
	Couma	Ethiopia/1961b	10236	117	465
	Serie227	Ethiopia/1961a	10236	117	465
West Africa I	BA55	Nigeria/1987b	10236	117	507
	69056	Nigeria/1946	10236	117	507
West Africa II	FVV	Senegal/1927	10236	117	507
	Asibi	Ghana/1927	10236	117	507

All viruses underwent NGS, and the consensus genome sequence for each virus was determined. Since the 5’ untranslated regions (UTR) of *Orthoflaviviruses* are very consistent in length and nucleotide content, while the 3’UTRs vary in length (Table 1), we focused our consensus sequence analysis on the nucleotide open reading frame (ORF) and translated the amino acid polyprotein coding region of the genomes, excluding both 5’ and 3’ UTRs. The consensus sequences of the 21 strains were used to construct a phylogenetic tree with WNV serving as the outgroup. Bootstrap values (100 bootstrap replicates) were incorporated to provide statistical support for our phylogenetic tree (Figure 1). This allowed for the confident visualization of the “age” of the viruses and the examination of how closely strains/genotypes were related to each other (Table 2). Figure 1 shows that WAI/WAII and SAI/ SAII genotypes were closely related but divergent from the EA, ECA, and ANG genotypes, which was consistent with our previously reported research and confirmed the validity and reproducibility of our dataset [7,8,14]. The consensus sequence of an ANG isolate from 2016 (Genbank KX010994.1) was included in our phylogenetic tree and analysis of genotype-specific amino acids (GSaas) so that our results for the ANG genotype did not depend on one strain, which was isolated in 1971 (Table 2 and Figure 1). As reported previously, the 1971 and 2016 ANG isolates are genetically very similar [13].

**Figure 1.**
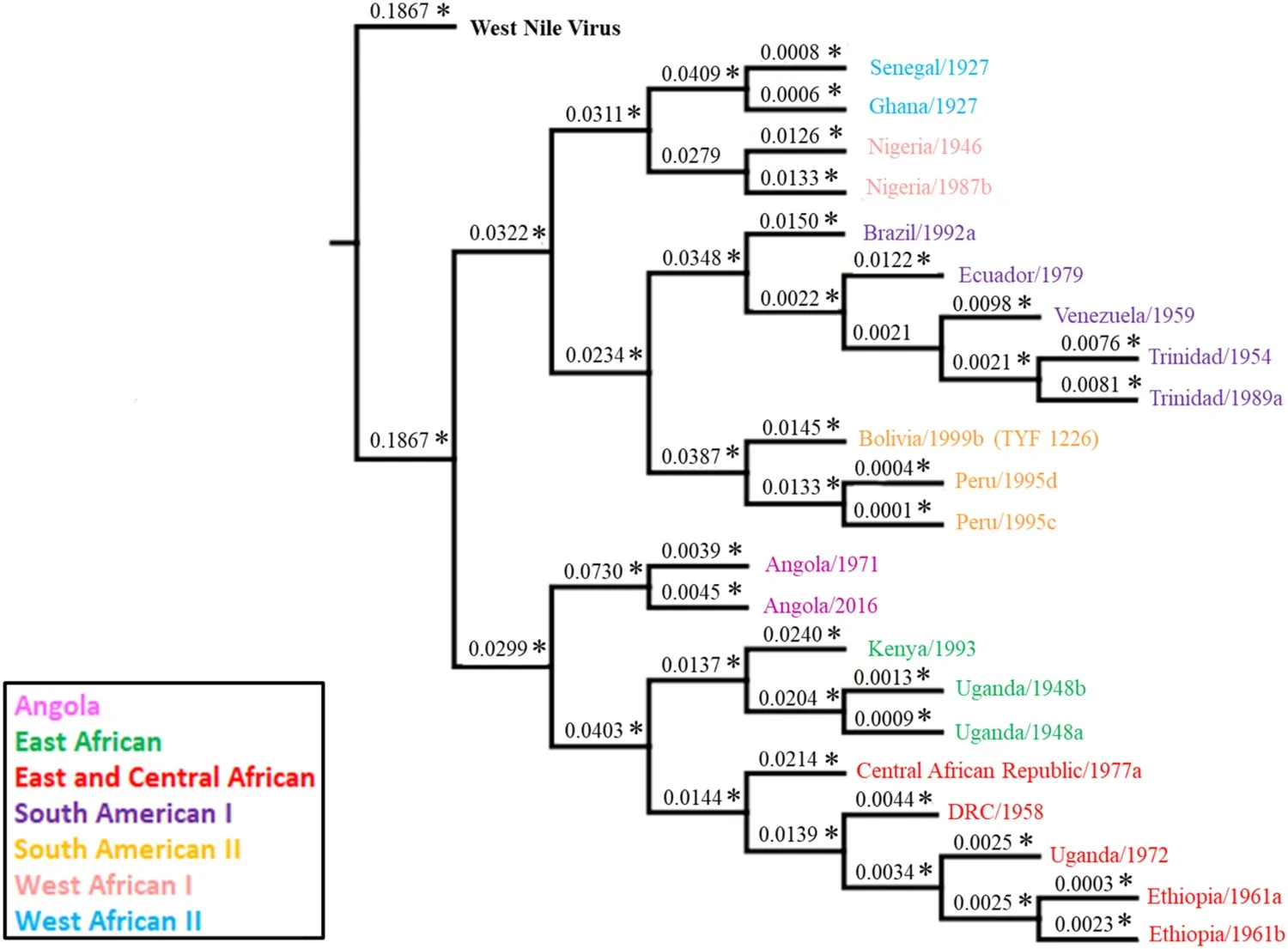
Phylogenetic tree depicting genotype-specific amino acids for EA and ECA. This phylogenetic tree was generated using Clustal with 100 bootstrap replicates (nodes with bootstrap values > 80% are designated with an *) and depicts the relationships between strains within each genotype and between each genotype. Genotype-specific amino acids that separate the EA and ECA were labelled at each node and discussed in the corresponding boxes. A. lists changes resulting in the divergence of the EA genotype (two total). B. lists changes resulting in the divergence of the ECA genotype (three total).

**Table 2.** The mutation frequency of the open reading frame for each genotype (nucleotide substitutions per site) of each strain used in the study. The nucleotide (nt) substitution per site values of the 7 genotypes of WT YFV.

Genotype	Substitutions per Site (nt)
Angola	0.0875
East Africa	0.0143
East and Central Africa	0.0165
South America I	0.0413
South America II	0.0411
West Africa I	0.0441
West Africa II	0.0315

The mutation frequencies of each isolate were calculated using Geneious software (Table 2). There were differences between genotypes in the number of nucleotide substitutions per site in the ORF region with ANG containing the most (0.0875), EA the fewest (0.0143), and the other genotypes falling in-between 0.0713 (SAI), 0.0411 (SAII), 0.0165 (ECA), 0.0441 (WAI), and 0.0315 (WAII) (Table 2). However, the number of substitutions per site of all seven genotypes did not vary significantly.

### Genetic diversity varies significantly between genotypes of WT YFV

NGS was also used to examine the genetic diversity of the individual strains used in this study. All samples were downsampled to 723 reads per nucleotide to consistently compare SE and SNVs for each strain. We analysed SE individually for each virus and averaged SEs of all viruses within each genotype and found that all genotypes contained similar levels of SE (0.0032 [ANG] to 0.0038 [ECA]) (Table 3). We analysed SNVs in the ranges >1.00%, >5.00%, and >10.00%, plus coding SNVs, to identify differences in the prevalence of SNVs for each of the genotypes. Overall, there were differences in both SE and SNVs between genotypes, but the strains within a particular genotype exhibited comparable levels of both characteristics. The ECA genotype contained the greatest number of SNVs, while the WAII viruses contained the fewest (Table 3). ECA genotype strain Ethiopia 1961b had the highest number of SNVs of all strains tested. A second preparation of Ethiopia 1961b was then analysed, and the results were confirmed. One possible explanation for the large number of SNVs found in Ethiopia 1961b is that it underwent many more passages in mouse brain than Ethiopia 1961a but, interestingly, it had fewer passages than Nigeria/1946 (46 vs 61 mouse brain passages), which did not show a greater number of SNVs than other strains in the same genotype (Table 3, Supplementary Figures 1 and 2, and Supplementary Table 1). Strains from the WAI (Nigeria/1946) and WAII (Senegal/1927) genotypes exhibited SE and SNVs similar to the SAI (Brazil/1992a) and SAII (Peru/1995d) genotypes (Figure 2(A–D) and Figure 3(A–D), respectively). The EA (Kenya/1993), ANG (Angola/1971), and ECA (Ethiopia/1961) genotypes exhibited slightly higher SE and numbers of SNVs from the WAI/WAII/SAI/SAII viruses, although these increases did not reach statistical significance (Figure 2(E–G), and Figure 3(E–G), respectively).

**Figure 2.**
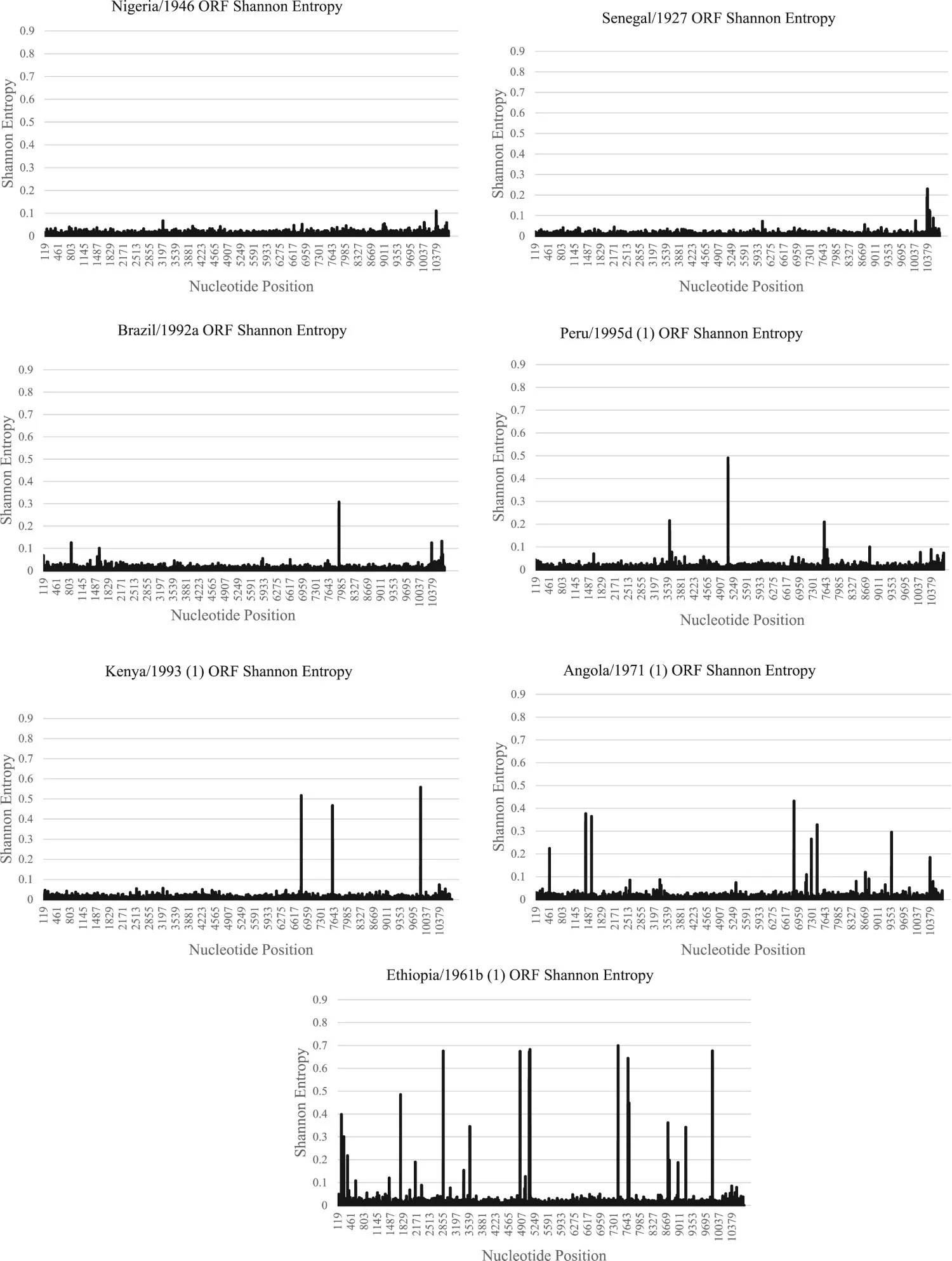
Examples of Shannon entropy per genotype. Graphical representation of the SE for a strain from each of the 7 genotypes of WT YFV. Only the coding regions of each sample were included (Nucleotides 119-10700). WAI genotype is represented by Nigeria/1946 (A). WAII genotype by Senegal/1927 (B). SAI genotype by Brazil/1992a (C). SAII genotype by Peru/1995d (D). EA genotype by Kenya/1993 (E). ANG genotype by Angola/1971 and (F). ECA genotype by Ethiopia/1961b (G). The graphs for other isolates used in these studies can be found in Supplementary Figure 1.

**Figure 3.**
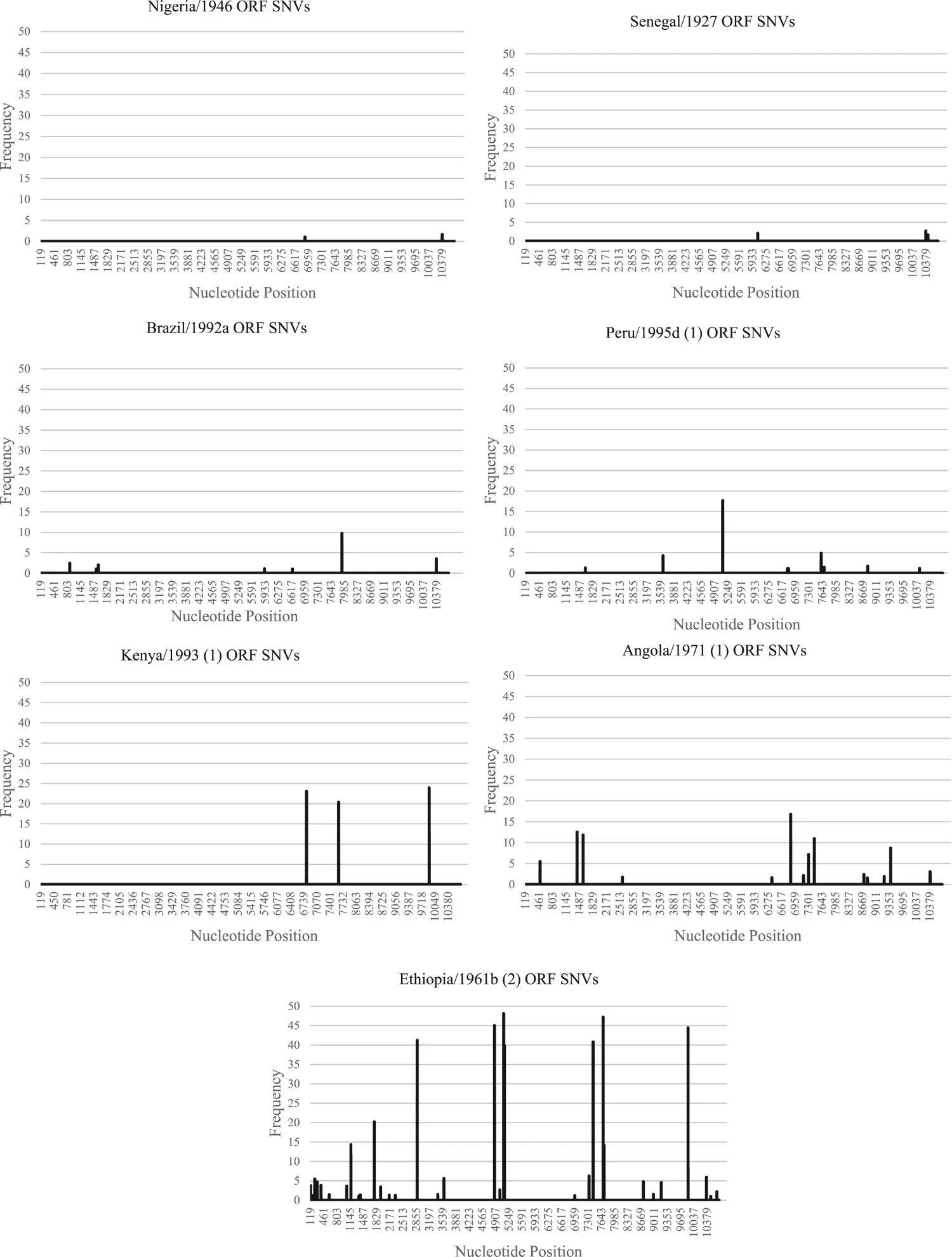
Examples of SNVs per genotype. Graphical representation of the SNV content for a strain from each of the 7 genotypes of WT YFV. Only the coding regions of each sample were included (Nucleotides 119-10700). WAI genotype is represented by Nigeria/1946 (A). WAII genotype by Senegal/1927 (B). SAI genotype by Brazil/1992a (C). SAII genotype by Peru/1995d (D). EA genotype by Kenya/1993 (E). ANG genotype by Angola/1971 and (F). ECA genotype by Ethiopia/1961b (G). The graphs for other isolates used in these studies can be found in Supplementary Figure 2.

**Table 3.** Passage history, Shannon entropy, and SNV data for each genotype. Passage histories of each virus preparation used in the study. The SE, SNVs > 1.00%, SNVs>5.00%, SNVs>10.00%, and coding SNVs of each virus preparation are listed individually for each genotype. The average and standard deviation of the genotype are listed in the corresponding columns. SM = suckling mouse brain.

Genotype	Strain	Taxon	Total passage history	Shannon entropy	Average Shannon entropy	Std. Dev SE	SNVs > 1.00%	SNVs > 5.00%	SNVs > 10%	Coding SNVs
Angola	14FA	Angola/1971	SM 7, Mosq. 1, Vero 4	0.0032	0.0032	N/A	15	7	4	2
South America I	1337	Ecuador/1979	Vero 1	0.0032	0.0031	0.0005	7	0	0	0
	P128MC	Venezuela/1959	SM 3, Mosq. 1, Vero 1	0.0036			13	7	6	6
	GML902621	Trinidad/1954	Monkey 1, Vero 1	0.0033			7	1	1	1
	BeAR 512943	Brazil/1992a	C6/36 1, Vero 1	0.0029			7	1	0	0
	CAREC 890692	Trinidad/1989a	C6/36 2	0.0023			2	1	0	0
South America II	OBS 7549	Bolivia/1999b	Vero 1	0.0030	0.0032	0.0002	13	6	3	3
	149	Peru/1995D	Vero 2	0.0034			10	1	1	1
	HEB 4236 (156)	Peru/1995c	Vero 3	0.0033			13	13	6	5
East Africa	BC7914	Kenya/1993	SM 1, C6/36 1, Vero 1	0.0029	0.0030	0.0001	5	4	4	3
	MR896	Uganda/1948b	C6/36 1, Vero 1	0.0031			9	6	6	6
	A709-4-A2	Uganda/1948a	C6/36, Vero 1	0.0031			3	0	0	0
East and Central Africa	ArB8883	Central African Republic/1977a	SM 5, Mosq. 1, Vero 1	0.0039	0.0038	0.0005	21	6	2	2
	STA-LSF-4-4143	DRC/1958	SM 2, Mosq. 1, Vero 1	0.0031			13	6	6	4
	Z19039	Uganda/1972	C6/36 1, SM 3, Vero 1	0.0037			19	5	5	4
	Couma	Ethiopia/1961b	SM 46, Mosq. 2, Vero 1	0.0045			35	14	13	12
	Serie227	Ethiopia/1961a	SM 7, Mosq. 1, Vero 1	0.0036			27	7	6	5
West Africa I	BA55	Nigeria/1987b	SM 3	0.0038	0.0034	0.0006	12	2	2	1
	69056	Nigeria/1946	SM 61	0.0029			3	0	0	0
West Africa II	FVV	Senegal/1927	C6/36 1	0.0024	0.0026	0.0002	5	0	0	0
	Asibi	Ghana/1927	Vero 2	0.0027			16	5	2	2

An analysis of variance (ANOVA) was performed using RStudio to evaluate the significance of differences in SE values and SNV frequencies among genotypes. While no significant differences were found in mean SE values or in SNV frequencies exceeding 5.00% and 10.00%, a significant difference was observed for SNV frequencies >1.00% among genotypes (F = 4, df = 20, *p* = 0.0099). Post-hoc Tukey HSD tests indicated that the ECA genotype had significantly more SNVs >1.00% compared to the EA genotype (Q critical value of 14.98 and Absolute mean difference 17.33) and the WAI genotype (Q critical value of 14.18 and Absolute mean difference 15.50) (Supplementary Tables 2–8 show statistical analysis).

### The passage history of a particular virus does not affect genetic diversity

To determine the potential impact of passage history of strains on genetic diversity, eight virus preparations were included in our analysis with different passage histories (i.e. one to four passages in Vero cells) (Supplementary Table 1). These strains included a different passage of Ethiopia/1961b (ECA genotype), duplicate preparations of Kenya/1993 and Uganda/1948a (EA genotype), Angola/1971 strain (ANG genotype), and Ecuador/1979 (SAI genotype) at the same passage (Supplementary Table 1). As expected, consensus genome analysis showed strong homology between passages of the same viral strain (data not shown). SE and SNV analyses highlighted that passage history did not significantly alter genetic diversity for a particular strain. (Supplementary Table 1 and Supplementary Figure 1). Furthermore, a more in-depth analysis of SNV data for the categories of all >1.00%, >5.00%, and >10%, plus SNVs that encode AA substitutions, revealed that virus preparations differing in passage history of the same strain also did not differ significantly from each other (data not shown).

### YFV genotypes contain genotype-specific amino acids (GSaas)

Using the Geneious software program, consensus polyprotein sequences were generated for each of the 21 different virus strains representing the seven YFV genotypes. Analysis of the consensus polyprotein sequences revealed 420 amino acid differences among the seven genotypes. Of these differences, 88 were genotype-specific amino acids (GSaas) that were only found in a single genotype (Table 4); thus, distinguishing a particular genotype from the other genotypes (Figure 1). The SAII and the ANG genotypes exhibited the most GSaas with 26 and 23, respectively (Table 4). The SAII genotype had GSaas in all 10 viral proteins and the ANG genotype in all except the E protein. In comparison, the EA and ECA genotypes contained the fewest GSaas with two and three, respectively (Table 5). Further analysis of the amino acid differences exhibited by EA and ECA genotypes revealed that most substitutions were comparable to the “original” amino acid in terms of charge, polarity, molecular weight, and size (Table 5). For the EA genotype, both GSaas occurred in NS5, while in the ECA genotype, one GSaas was found in each of NS4A, NS4B, and NS5. The WAI, WAII, and SAI genotypes had 7, 13, and 13 GSaas, respectively (Table 4). Both the SAI and WAI genotypes had GSaas in five proteins (C, E, NS1, NS4A and NS5, and C, E, NS2A, NS4B and NS5, respectively), while GSaas were found in seven viral proteins (C, E, NS1, NS2B, NS3, NS4B and NS5) for the WAII genotype viruses.

**Table 4.** Genotype-specific amino acids. The genotype-specific amino acids for each genotype are listed in the corresponding columns. The protein, conical amino acid is listed before the amino acid number, followed by the genotype-specific amino acid (GSaa) (i.e. the GSaa occurring in the ANG genotype at position 114 within the C protein is listed as C: M-114-L).

Angola (23)	East Africa (2)	East and Central Africa (3)	South America I (14)	South America II (26)	West Africa I (7)	West Africa II (12)
C: M-114-L	NS5:R-673-K	NS4A: V-146-I	C: N-24-S	C: R-17-Q	C: S-89-G	prM: A-48-T
M: M-57-T	NS5: N-710-S	NS4B: K-16-R	C: N-35-S	C: K-58-R	E:V-430-I	E:S-450-N
NS1: V-172-T		NS5: V-169-I	C: I-47-V	prM: N-7-S	NS2A: V-56-M	NS1: T-218-A
NS1: H-256-R			E: H-67-N	prM: Y-39-N	NS4B: K-8-R	NS1: D-240-E
NS2A: L-54-F			E: A-83-E	E: N-90-H	NS5: D-275-N	NS2B: K-126-R
NS2A: L-155-M			E: R-207-K	E: T-154-A	NS5: Q-635-H	NS3: K-397-R
NS2A: V-158-M			E: N-272-K	E: A-343-S	NS5: L-681-M	NS3: I-515-V
NS2B: V-59-I			E:S-421-G	E: D-360-E		NS3: R-535-K
NS3: K-119-R			E:A-459-V	E: T-436-M		NS3: A-613-S
NS3: H-241-Q			NS1: *P*-93-S	E: T-468-M		NS4B: T-122-S
NS3: S-551-A			NS4A: M-24-V	NS1: I-19-V		NS5: T-293-S
NS4A: V-8-I			NS4A: I-53-T	NS1: S-343-N		NS5: D-658-N
NS4A: M-67-A			NS5: K-96-R	NS2A: V-20-F		
NS4B: N-18-D			NS5: T-231-A	NS2A: R-22-K		
NS4B: G-249-E				NS2A: N-146-S		
NS5: L-27-M				NS2B: V-42-I		
NS5: V-182-I				NS2B: L-115-M		
NS5: A-388-S				NS3: T-10-A		
NS5: V-570-I				NS3: I-14-V		
NS5: D-641-G				NS3: S-70-T		
NS5: K-805-R				NS3: K-178-R		
NS5: V-835-I				NS4A: H-32-N		
NS5: Y-884-F				NS4B: S-29-N		
				NS4B: V-99-I		
				NS5: D-39-E		
				NS5: I-632-V

**Table 5.** Original amino acid compared to the genotype-specific amino acids for EA and ECA genotypes. Information regarding amino acid type and amino acid molecular weight for each EA and ECA genotype-specific acids is listed along with the molecular weight (MW) differences.

Genotype	Original amino acid type	Changed amino acid type	Original amino acid MW	Changed amino acid MW	MW difference
East Africa (2)					
NS5:R-673-K	Positive	Positive	174.20	146.20	28.00
NS5: N-710-S	Polar Uncharged (Amide)	Polar Uncharged (Nucleophile)	132.10	105.09	27.01
East and Central Africa (3)					
NS4A: V-146-I	Hydrophobic	Hydrophobic	117.10	131.20	−14.10
NS4B: K-16-R	Positive (Basic)	Positive (Basic)	146.20	174.20	−28.00
NS5: V-169-I	Hydrophobic	Hydrophobic	117.10	131.20	−14.10

While GSaas were found in all YFV-encoded proteins (Table 4), only NS3, NS5, and E proteins contained greater than seven GSaas. Eleven GSaas were in NS3 but were distributed between three genotypes (ANG [*n* = three], SAII [*n* = four], and WAII [*n* = four]). Twenty were in NS5, with all seven genotypes having at least one (ANG [*n* = eight], WAI [*n* = three], SAI, SAII, WAII [*n* = two each], and ECA [*n* = one]). This suggested there were selection pressures in the replication complex for each genotype. There were 14 E protein GSaas (Table 4), with most (12) in the two South American genotypes (*n* = six each) and only two among all five African genotypes, suggesting a strong selection pressure for E protein substitutions during the introduction of YFV from Africa into South America. Interestingly, the E protein GSaas were predominantly in the stem/anchor region rather than surface-exposed.

## Discussion

The studies reported here investigated differences in the level of genetic diversity among 21 early strains representing all seven genotypes of WT YFV using SE and SNV measurements. The strains used were the earliest examples of each genotype available to the authors. Thus, a limitation of our studies is that we examined early isolates from each genotype, and examination of more recent isolates may have generated different data. Clearly, recent viral isolates need to be examined in the future. Nonetheless, comparison of the strains used showed that while genetic diversity was comparable in six genotypes, it was much greater in the ECA genotype.

Our studies expand upon previous studies that utilized phylodynamic and phylogeographic time-scale estimation to show the evolutionary rates and dispersal of YFV across the African continent to South America, and the ratio of nonsynonymous (dN) to synonymous (dS) substitution rates for different strains and genotypes of YFV [5,7,8,14,15,16,20,22]. In contrast, we opted to use the total number of substitutions per site to allow for broader comparisons across genotypes. This approach was particularly advantageous given the size of our dataset and represents the most comprehensive analysis of variation among the seven recognized YFV genotypes undertaken to date. Our codon usage analysis indicated the ANG genotype exhibited the highest number of NT substitutions per site (0.0875), nearly double that of the genotype with the second-highest rate, WAI (0.0441 substitutions/site). This finding aligns with previous findings from our laboratory and others [1,3,5,7,8,14,17,18–20,21–24]. For instance, a prior study from our group reported 74.8%–100% nucleotide identity between the partial prM/E protein sequences among genotypes [14]. The present study expands on these findings by assessing the entire polyprotein and revealing a similar range of nucleotide identity (from 76% to 100%) to that reported study. These results further refine and expand our understanding of genetic homology across wild-type YFV genotypes.

Previous foundational studies conducted in our laboratory have been pivotal in advancing our understanding of YFV phylogeography and have contributed to the results presented here [5,7,8,14,15, 16,20,22]. The phylogenetic tree developed from our analysis based on the entire YFV ORF revealed similar nucleotide mutation frequencies among all genotypes (Figure 1). Our data suggest that as YFV moved westwards across the African continent, the viral RNA population became more homogenous, losing genetic diversity (Table 3). This aligns with other models of evolution showing that viral quasispecies are abundant, especially at the origin of virus emergence, further reinforcing current models of YFV spread [5,25,26]. As such, we were not surprised to find the ECA strains exhibited greater genetic diversity than the other genotypes. Nonetheless, genetic diversity did not correlate with the number of GSaa residues, suggesting different selection pressures in different geographic regions. Furthermore, analysis showed that the ratios of GSaas in each protein to the length of the protein were not proportional. Interestingly, many GSaas occurred in the NS proteins, which form the viral replication complex, but none were found in motifs important for protein function (data not shown). These data strongly suggest that selection pressure, not random mutations, occurred to alter the amino acids in YFV proteins in particular genotypes. Nonetheless, we cannot exclude the possibility that GSaas contribute to differences in the function of viral proteins. In addition, no antiviral drug has been licensed to treat YF disease, so we cannot speculate on whether or not any of the GSaas would be involved in the targets of such drugs. Such studies are beyond the scope of the current studies and would require reverse genetics to modify one or more GSaas to investigate their effects on the function of the viral protein(s) and or virion.

Other areas that would be of particular interest to examine include competency for particular mosquito species, or multiplication and clinical signs in NHP species due to the different geographical areas where particular genotypes are found. The ECA genotype contained the most genetic diversity but exhibited the second lowest number of GSaas (Table 4). One plausible explanation for the fewer GSaas could be differences in mosquito vectors in the transmission cycles, as these vary by geographic regions [20]. The five genotypes of the African continent (ANG, ECA, EA, WAI, WAII) utilize members of the *Aedes* genus of mosquitoes [22] while the South American YFVs are chiefly vectored by *Haemagogus* and *Sabethes* mosquitoes [27]. The precise mosquito species varies depending on the transmission cycle. Studies show that variation in codon usage in mosquito vectors, as well as differing NHP hosts, can affect viral genetic diversity [7,14,15]. These studies relate codon usage variation to viral responses to differing species, leading to mutations whereby the codon usage becomes altered to allow the virus to optimally harness the cellular machinery of the particular host [14,15,22–24]. In comparison, other studies have shown that codon usage between ECA and WAI/WAII genotypes does not differ significantly, suggesting that transmission cycle variation between the genotypes does not substantially contribute to the GSaas [7,14,15]. Clearly, additional work is needed in this area to draw cohesive conclusions on viral codon usage.

Furthermore, there were variations between SAII and SAI genotypes in terms of GSaas, with 26 and 13, respectively (Table 4). One plausible explanation for this difference can be found when examining the ecology of these genotypes. Previous research from our lab has shown that SAII tends to occur in geographic foci, isolates vary in nucleotide sequence by geographic area, and particular strains stay in one specific geographic area. In contrast, SAI moves across the continent in waves [7,8,20]. This may contribute to why the SAII genotype contains more GSaas. We believe that the SAI genotype and WAI/WAII genotypes contain similar numbers of GSaas due to how YFV spread from Africa to the Western Hemisphere [5,8].

Interestingly, the ANG genotype contains nine more GSaas than the second-highest genotype (23 vs. 14) (Table 4). We infer this is likely due to the emergence pattern and apparent lack of viral evolution of YFV in the country, as evidenced by the fact that there are only two isolates of YFV from Angola, one from 1971 and a second from 2016, both of which exhibit extremely high sequence homology [13], despite a 45-year difference in collection years. The 23-GSaas for the ANG genotype needs to be considered with care, as we only have two examples in our studies. The YFV transmission cycle in Angola is poorly understood, requiring further studies to fully elucidate the lack of viral emergence and evolution.

As the EA and ECA genotypes exhibit high genetic diversity, but low numbers of GSaas, we were interested in examining the possible role of the GSaas on the structure–function of each protein (NS4A, NS4B, and NS5). As shown in Table 5, the majority of the GSaas are similar in molecular weight and amino acid properties to those occurring in other genotypes. The only exception to this is the EA GSaa of NS5: N-710-S, where the substitution alters the functional group from an amide to a nucleophile. This is the only EA and ECA GSaa that we believe could alter the structure–function of the viral protein. The EA genotype has only two GSas, both in NS5 (NS5 R-673-K and N-710-S). Figure 4 shows the structure of NS5 RdRp, showing the spatial locations of NS5-673 and NS5-710, and as can be seen, they are not close to each other in the structure. NS5 residue 710 is located in the α31 helix E motif, which contributes to the active site of NS5 (along with motifs A, B, D, and G) [9,31–33]. This residue also plays a role in the stabilization of the secondary zinc binding finger, thus contributing to stability through coordination of metal ions used in nucleotide polymerization as well as interactions with the RNA product [16,32,33]. In particular, the motif binds with incoming nucleotides to ensure that RNA/NS5 binding is stable and in the proper conformation [9,31–33]. Typically, amino acids are linked via peptide bonds, but the alteration of a functional group may impact the function of the domain of NS5, as it is in the active site of the protein, particularly as nucleophiles are common components of enzymes and are able to form new bonds through the donation of electrons. The alteration of a functional group, particularly within a motif forming the active site, could significantly impact the function of the RdRp. Coupled with the proximity to the second zinc binding finger, this GSaa has the capability to affect the function of the RdRp in EA YFV strains.

**Figure 4.**
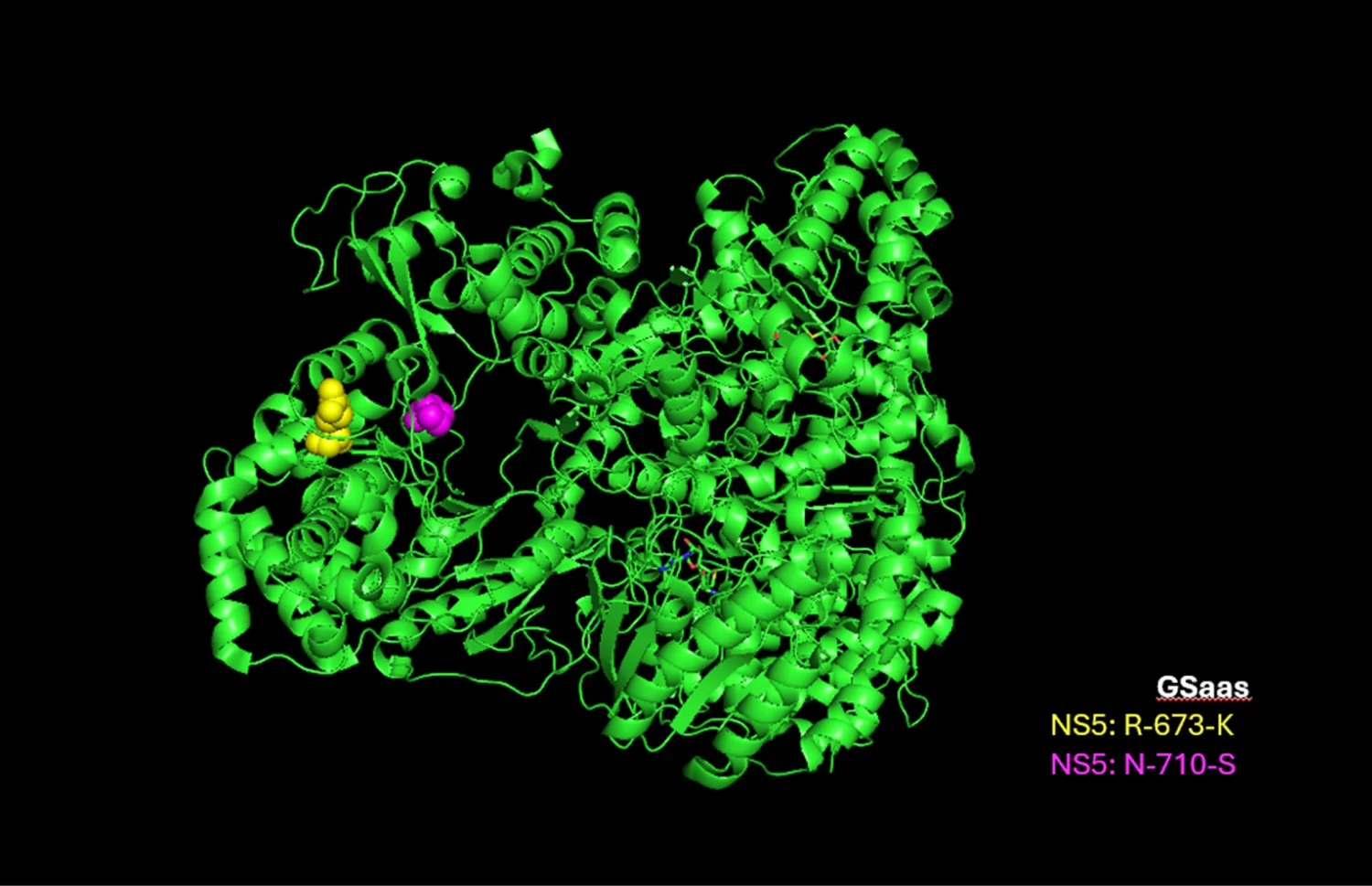
NS5 Structural modelling of NS5 EA GSaas. A 3D structure of the YFV NS5 protein (PDB: 6QSN) with the GSaa residues highlighted as spheres. EA GSaa R-673-K is shown in yellow, and N-710-S is depicted in yellow.

Overall, our studies provide important new information regarding variation in genetic diversity between the seven genotypes of WT YFV and identify GSaas that distinguish each genotype from the others. We showed that genetic diversity does not correlate with the occurrence of GSaas, as the ECA contains the greatest amount of genetic diversity, but the second fewest number of GSaas. Furthermore, we expand upon previous knowledge regarding the spread of YFV through the incorporation of the entire ORF in our analysis. Interestingly, our data reveal that there is selective pressure occurring in the South American YFV viruses for alterations in the E protein. We postulate this indicates increased involvement/importance of the E protein in the adaptation of YFV in South America. Taken together with previous literature, we believe this is at least in part due to different NHP hosts and mosquito vectors in different geographic areas. Thus, our data provides the most comprehensive look at YFV genetic diversity to date and provides key insight into the evolution of YFV as it spread across the African continent and into South America.

## Nucleotide sequence data

Nucleotide sequences can be found in GenBank with accession numbers PX246671 to PX246691. Next Generation Sequencing data can be found at BioProject ID: PRJNA1312863.

## Supplementary Material

Lee et al Supplementary Data 6 9 25.pdf

## References

[1] Simon LV, Hashmi MF, Torp KD. Yellow fever. Treasure Island: StatPearls Publishing. 29262028

[2] Barros MC, Galasso TG, Chaib AJ, et al. Yellow fever virus envelope protein expressed in insect cells is capable of syncytium formation in lepidopteran cells and could be used for immunodetection of YFV in human sera. Virol J. 2011;8:261. doi:10.1186/1743-422x-8-261. Epub 20110527. PubMed PMID: 21619598; PubMed Central PMCID: PMC3118360. PMC3118360 21619598

[3] Barrett AD, Higgs S. Yellow fever: a disease that has yet to be conquered. Annu Rev Entomol. 2007;52:209–229. doi:10.1146/annurev.ento.52.110405.091454. PubMed PMID: 16913829. 16913829

[4] Gianchecchi E, Cianchi V, Torelli A, et al. Yellow fever: origin, epidemiology, preventive strategies and future prospects. Vaccines. 2022;10:3. doi:10.3390/vaccines10030372. PubMed PMID: 35335004; PubMed Central PMCID: PMC8955180. PMC8955180 35335004

[5] Beck A, Guzman H, Li L, et al. Phylogeographic reconstruction of African yellow fever virus isolates indicates recent simultaneous dispersal into east and west Africa. PLoS Negl Trop Dis. 2013;7(3):e1910. doi:10.1371/journal.pntd.0001910. PubMed PMID: 23516640; PubMed Central PMCID: PMC3597480. PMC3597480 23516640

[6] Beck A, Tesh RB, Wood TG, et al. Comparison of the live attenuated yellow fever vaccine 17D-204 strain to its virulent parental strain Asibi by deep sequencing. J Infect Dis. 2014;209(3):334–344. doi:10.1093/infdis/jit546. PubMed PMID: 24141982; PubMed Central PMCID: PMC3883177. PMC3883177 24141982

[7] Bryant JE, Barrett AD. Comparative phylogenies of yellow fever isolates from Peru and Brazil. FEMS Immunol Med Microbiol. 2003;39(2):103–118. doi:10.1016/s0928-8244(03)00238-4. PubMed PMID: 14625093. 14625093

[8] Bryant JE, Holmes EC, Barrett AD. Out of Africa: a molecular perspective on the introduction of yellow fever virus into the Americas. PLoS Pathog. 2007;3(5):e75. doi:10.1371/journal.ppat.0030075. PubMed PMID: 17511518; PubMed Central PMCID: PMC1868956. PMC1868956 17511518

[9] Davis EH, Beck AS, Strother AE, et al. Attenuation of live-attenuated yellow fever 17D vaccine virus is localized to a high-fidelity replication complex. mBio. 2019;10(5):e02294–19. doi:10.1128/mBio.02294-19 PMC6805994 31641088

[10] Strother AE, Thompson JK, Widen SG, et al. Genetic diversity does not contribute to attenuation of HeLa passaged wild-type yellow fever virus strain french viscerotropic virus. Viruses. 2022;14(3):527. doi:10.3390/v14030527. PMC8949127 35336933

[11] Chen Z, Liu L, Lv Y Z, et al. A fatal yellow fever virus infection in China: description and lessons. Emerging Microbes Infect. 2016;5(1):1–8. doi:10.1038/emi.2016.89 PMC5141266 27406389

[12] McMullan LK, Frace M, Sammons SA, et al. Using next generation sequencing to identify yellow fever virus in Uganda. Virology. 2012;422(1):1–5. doi:10.1016/j.virol.2011.08.024 21962764

[13] Chen C, Jiang D, Ni M, et al. Phylogenomic analysis unravels evolution of yellow fever virus within hosts. PLoS Negl Trop Dis. 2018;12(9):e0006738. doi:10.1371/journal.pntd.0006738. PubMed PMID: 30188905; PubMed Central PMCID: PMC6143276. PMC6143276 30188905

[14] Mutebi JP, Wang H, Li L, et al. Phylogenetic and evolutionary relationships among yellow fever virus isolates in Africa. J Virol. 2001;75(15):6999–7008. doi:10.1128/jvi.75.15.6999-7008.2001. PubMed PMID: 11435580; PubMed Central PMCID: PMC114428. PMC114428 11435580

[15] von Lindern JJ, Aroner S, Barrett ND, et al. Genome analysis and phylogenetic relationships between east, central and west African isolates of Yellow fever virus. J Gen Virol. 2006;87(4):895–907. doi:10.1099/vir.0.81236-0. PubMed PMID: 16528039. 16528039

[16] Wang E, Weaver SC, Shope RE, et al. Genetic variation in yellow fever virus: duplication in the 3’ noncoding region of strains from Africa. Virology. 1996;225(2): 274–281. doi:10.1006/viro.1996.0601. PubMed PMID: 8918913. 8918913

[17] da Fonseca NJ, Lima Afonso MQ, Pedersolli NG, et al. Sequence, structure and function relationships in flaviviruses as assessed by evolutive aspects of its conserved non-structural protein domains. Biochem Biophys Res Commun. 2017;492(4):565–571. 10.1016j.bbrc.2017.01.041. 10.1016/j.bbrc.2017.01.041 28087275

[18] Klitting R, Riziki T, Moureau G, et al. Exploratory re-encoding of yellow fever virus genome: new insights for the design of live-attenuated viruses. Virus Evol. 2018;4(2):vey021. doi:10.1093/ve/vey021. PubMed PMID: 30057792; PubMed Central PMCID: PMC6057501. PMC6057501 30057792

[19] Lozada-Chávez AN, Lozada-Chávez I, Alfano N, et al. Adaptive genomic signatures of globally invasive populations of the yellow fever mosquito Aedes aegypti. Nat Ecol Evol. 2025;9(4):652–671. doi:10.1038/s41559-025-02643-5 PMC11976285 40155778

[20] Mutebi J-P, Barrett AD. The epidemiology of yellow fever in Africa. Microbes Infect. 2002;4(14):1459–1468. doi:10.1016/S1286-4579(02)00028-X 12475636

[21] Davis EH, Thompson JK, Widen SG, et al. Genome characterization of yellow fever virus wild-type strain asibi, parent to live-attenuated 17D vaccine, from three different sources. Viruses. 2021;13(7):1383. doi:10.3390/v13071383. PubMed PMID: 34372589; PubMed Central PMCID: PMC8310116. PMC8310116 34372589

[22] Ellis BR, Barrett ADT. The enigma of yellow fever in East Africa. Rev Med Virol. 2008;18(5):331–346. doi:10.1002/rmv.584 18615782

[23] Hinman EH. Yellow Fever. Science. 1951;114(2951):77–78. doi:10.1126/science.114.2951.77.b

[24] Vasconcelos PF, Bryant JE, Rosa TP, et al. Genetic divergence and dispersal of yellow fever virus, Brazil. Emerg Infect Dis. 2004;10(9):1578–1584. doi:10.3201/eid1009.040197 PMC3320275 15498159

[25] Sardanyés J, Perales C, Domingo E, et al. Quasispecies theory and emerging viruses: challenges and applications. NPJ Viruses. 2024;2(1):54. doi:10.1038/s44298-024-00066-w. PMC11721110 40295874

[26] Singh K, Mehta D, Dumka S, et al. Quasispecies nature of RNA viruses: lessons from the past. Vaccines. 2023;11(2):308. doi:10.3390/vaccines11020308. PubMed PMID: 36851186; PubMed Central PMCID: PMC9963406. PMC9963406 36851186

[27] Stock NK, Laraway H, Faye O, et al. Biological and phylogenetic characteristics of yellow fever virus lineages from West Africa. J Virol. 2013;87(5):2895–2907. doi:10.1128/jvi.01116-12. PubMed PMID: 23269797; PubMed Central PMCID: PMC3571399. PMC3571399 23269797

[28] Leung JY, Pijlman GP, Kondratieva N, et al. Role of nonstructural protein NS2A in flavivirus assembly. J Virol. 2008;82(10):4731–4741. doi:10.1128/jvi.00002-08. PubMed PMID: 18337583; PubMed Central PMCID: PMC2346727. PMC2346727 18337583

[29] Tian L, Shen X, Murphy RW, et al. The adaptation of codon usage of + ssRNA viruses to their hosts. Infect Genet Evol. 2018;63:175–179. doi:10.1016/j.meegid.2018.05.034 PMC7106036 29864509

[30] Yao H, Chen M, Tang Z. Analysis of synonymous codon usage bias in flaviviridae virus. BioMed Res Int. 2019;2019(1):5857285. doi:10.11552019/5857285. PMC6620835 31346520

[31] Krejčová K, Krafcikova P, Klima M, et al. Structural and functional insights in flavivirus NS5 proteins gained by the structure of Ntaya virus polymerase and methyltransferase. Structure. 2024;32(8):1099–109.e3. doi:10.1016/j.str.2024.04.020. PubMed PMID: 38781970. 38781970

[32] Duan Y, Zeng M, Jiang B, et al. Flavivirus RNA-dependent RNA polymerase interacts with genome UTRs and viral proteins to facilitate flavivirus RNA replication. Viruses. 2019;11(10):929. doi:10.3390/v11100929. PubMed PMID: 31658680; PubMed Central PMCID: PMC6832647. PMC6832647 31658680

[33] Lu G, Gong P. Crystal Structure of the full-length Japanese encephalitis virus NS5 reveals a conserved methyltransferase-polymerase interface. PLoS Pathog. 2013;9(8):e1003549. doi:10.1371/journal.ppat.1003549. PubMed PMID: 23950717; PubMed Central PMCID: PMC3738499. PMC3738499 23950717

