# Supplementary material for: Differences in genetic diversity between early isolates of yellow fever virus genotypes
Source: Emerg Microbes Infect. 2025 Dec 15;15(1):2601371. doi: 10.1080/22221751.2025.2601371 (PMC13726306; doi:10.1080/22221751.2025.2601371)
Supplement: Supplementary file 1 [file TEMI_A_2601371_SM9015.pdf]

Supplementary Data

Supplementary Figure 1: SE graphs for each virus preparation used in our study.

A)

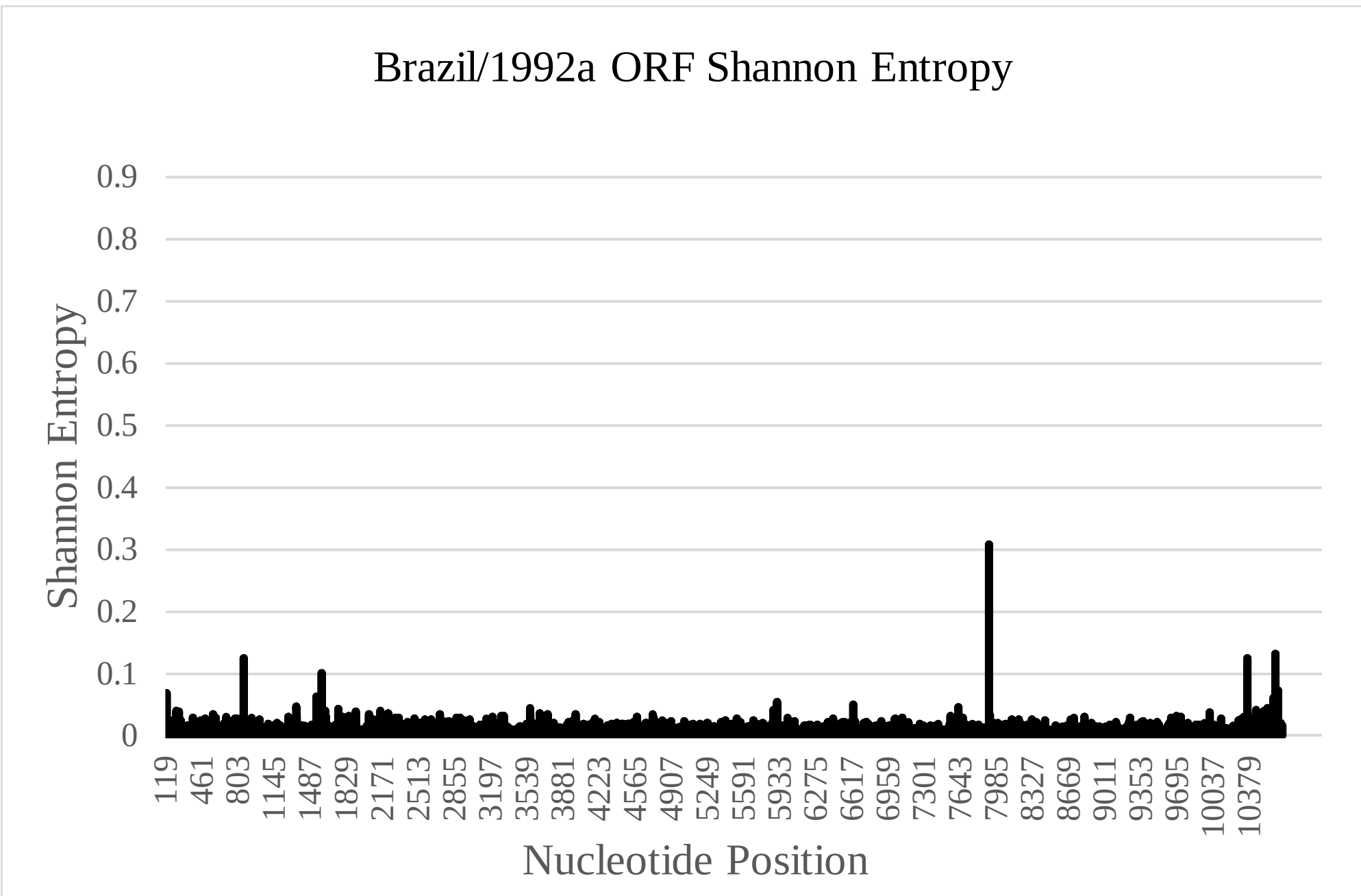

B)

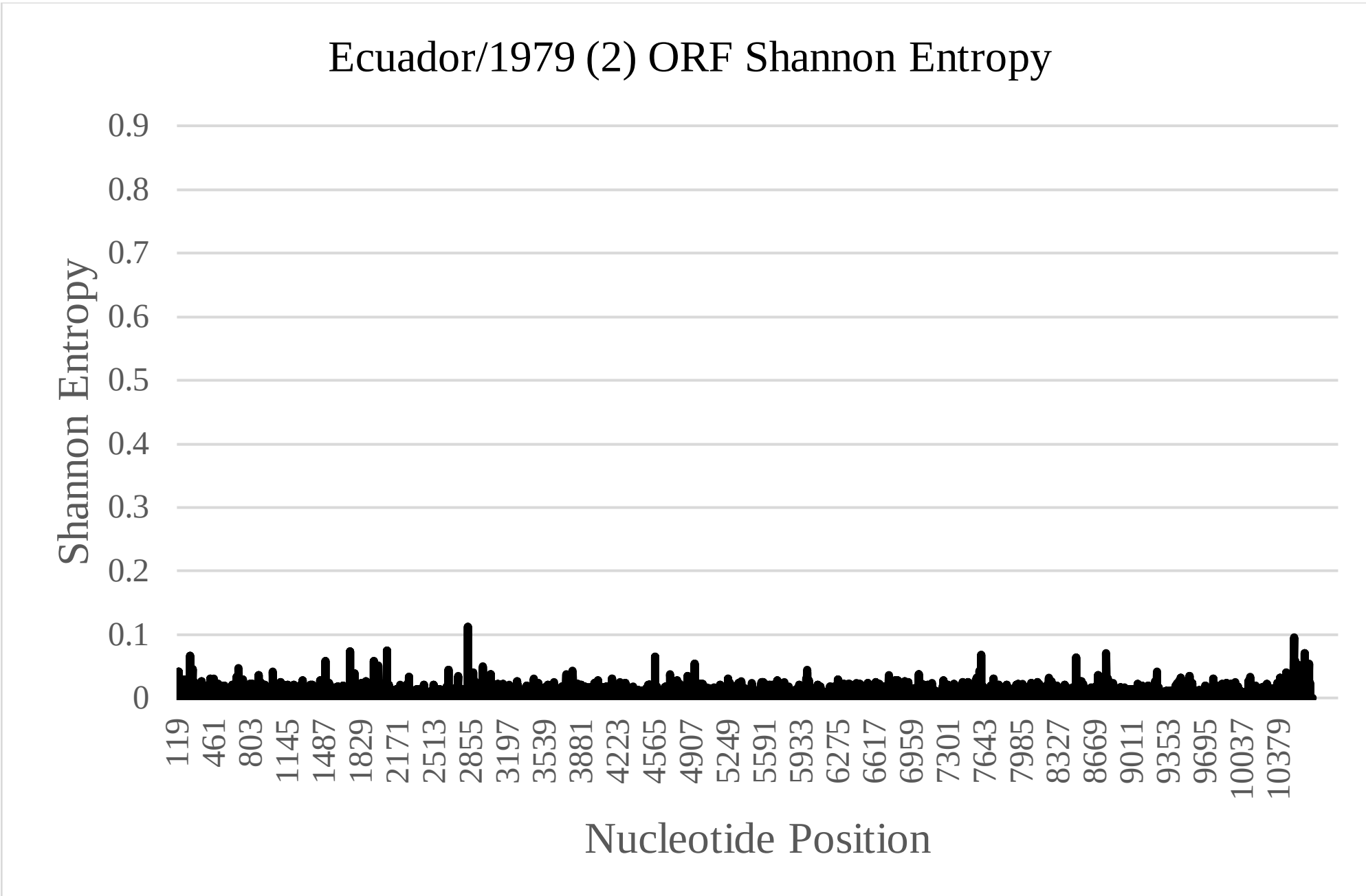
Ecuador/1979 (2) ORF Shannon Entropy
Shannon Entropy
0.9
0.8
0.7
0.6
0.5
0.4
0.3
0.2
0.1
0
119
461
803
1145
1487
1829
2171
2513
2855
3197
3539
3881
4223
4565
4907
5249
5591
5933
6275
6617
6959
7301
7643
7985
8327
8669
9011
9353
9695
10037
10379
Nucleotide Position

C)

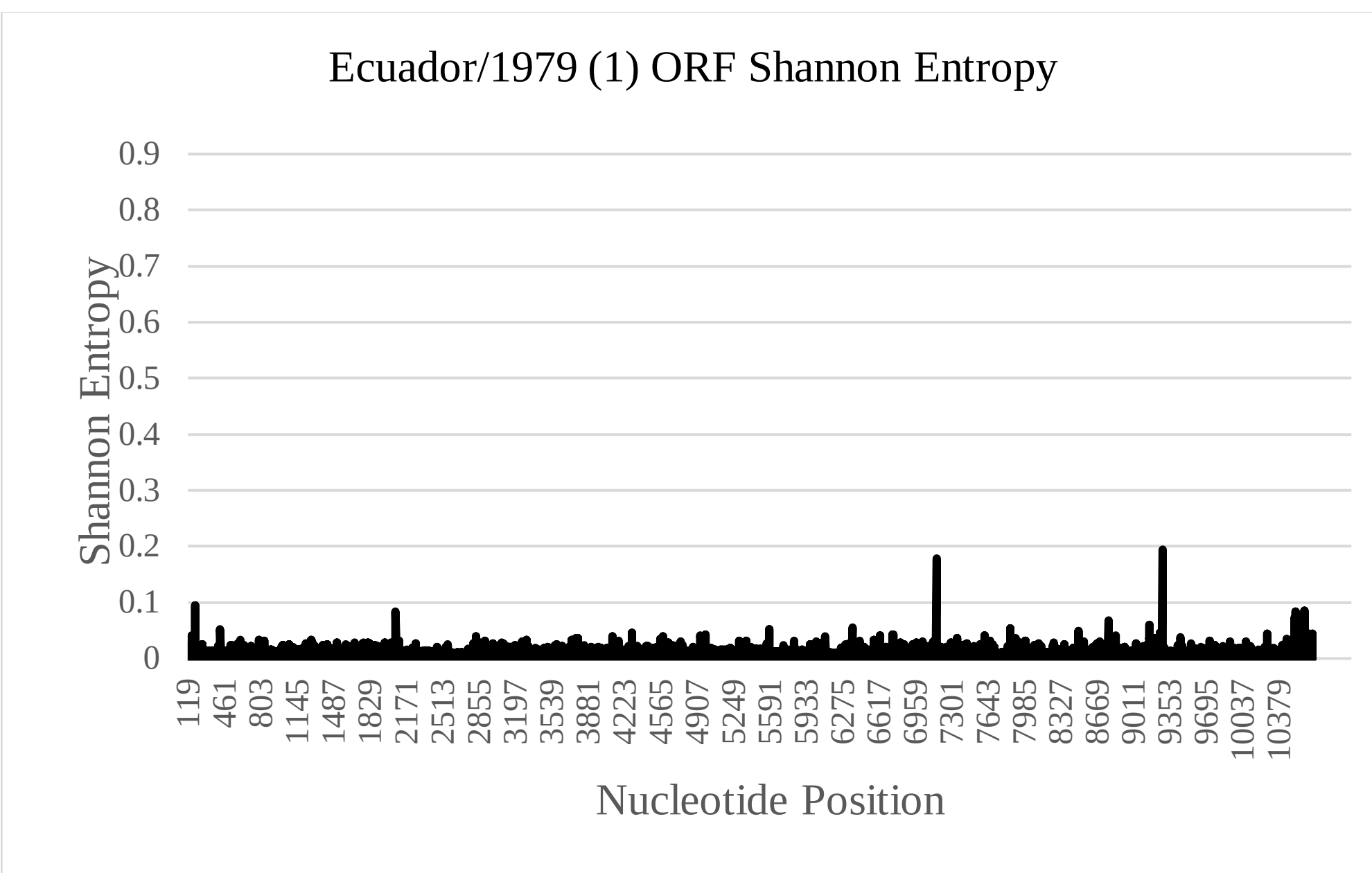
Ecuador/1979 (1) ORF Shannon Entropy
Shannon Entropy
0.9
0.8
0.7
0.6
0.5
0.4
0.3
0.2
0.1
0
119
461
803
1145
1487
1829
2171
2513
2855
3197
3539
3881
4223
4565
4907
5249
5591
5933
6275
6617
6959
7301
7643
7985
8327
8669
9011
9353
9695
10037
10379
Nucleotide Position

D)

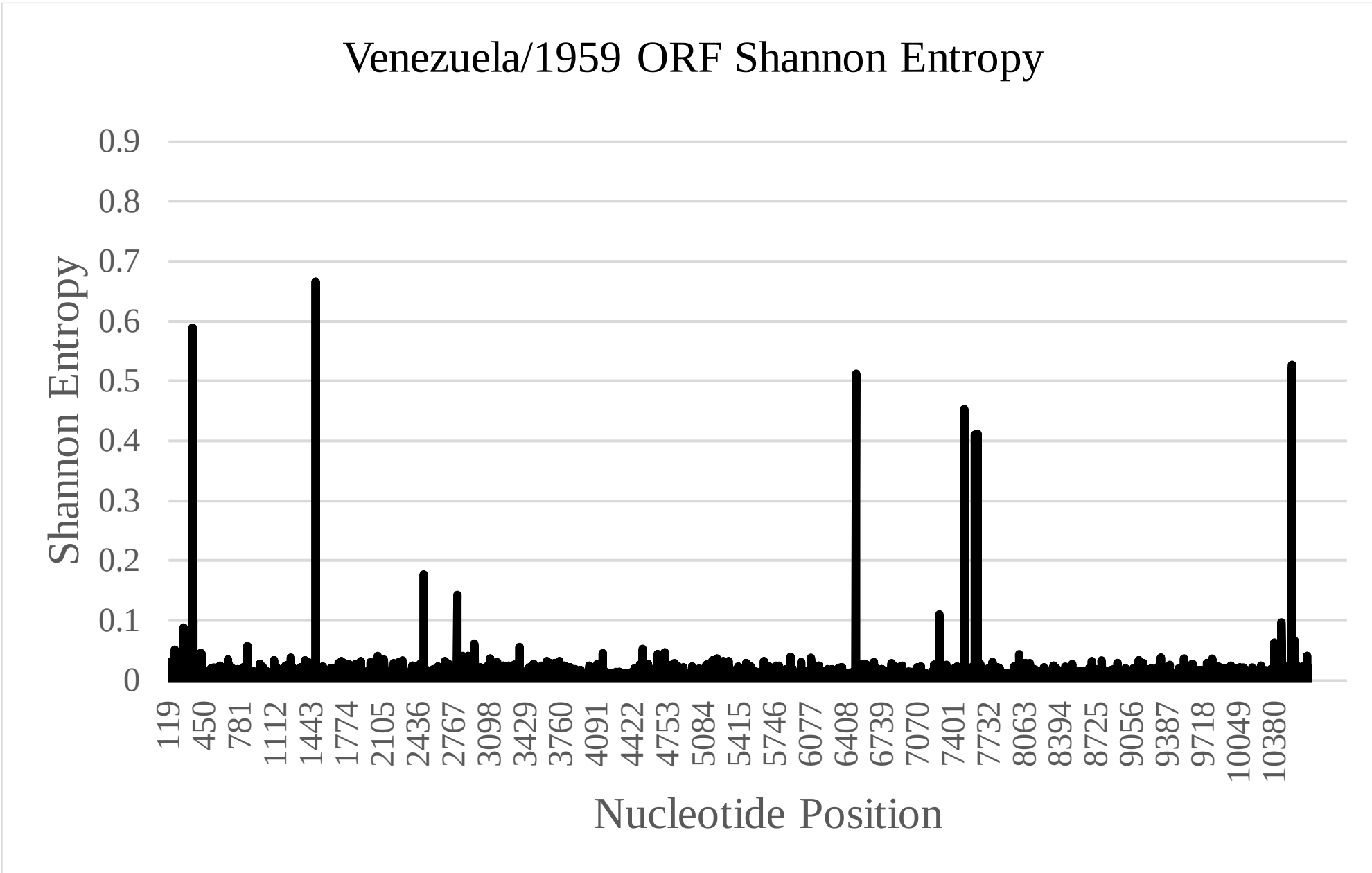

Venezuela/1959 ORF Shannon Entropy
Shannon Entropy
0.9
0.8
0.7
0.6
0.5
0.4
0.3
0.2
0.1
0
119
450
781
1112
1443
1774
2105
2436
2767
3098
3429
3760
4091
4422
4753
5084
5415
5746
6077
6408
6739
7070
7401
7732
8063
8394
8725
9056
9387
9718
10049
10380
Nucleotide Position

E)

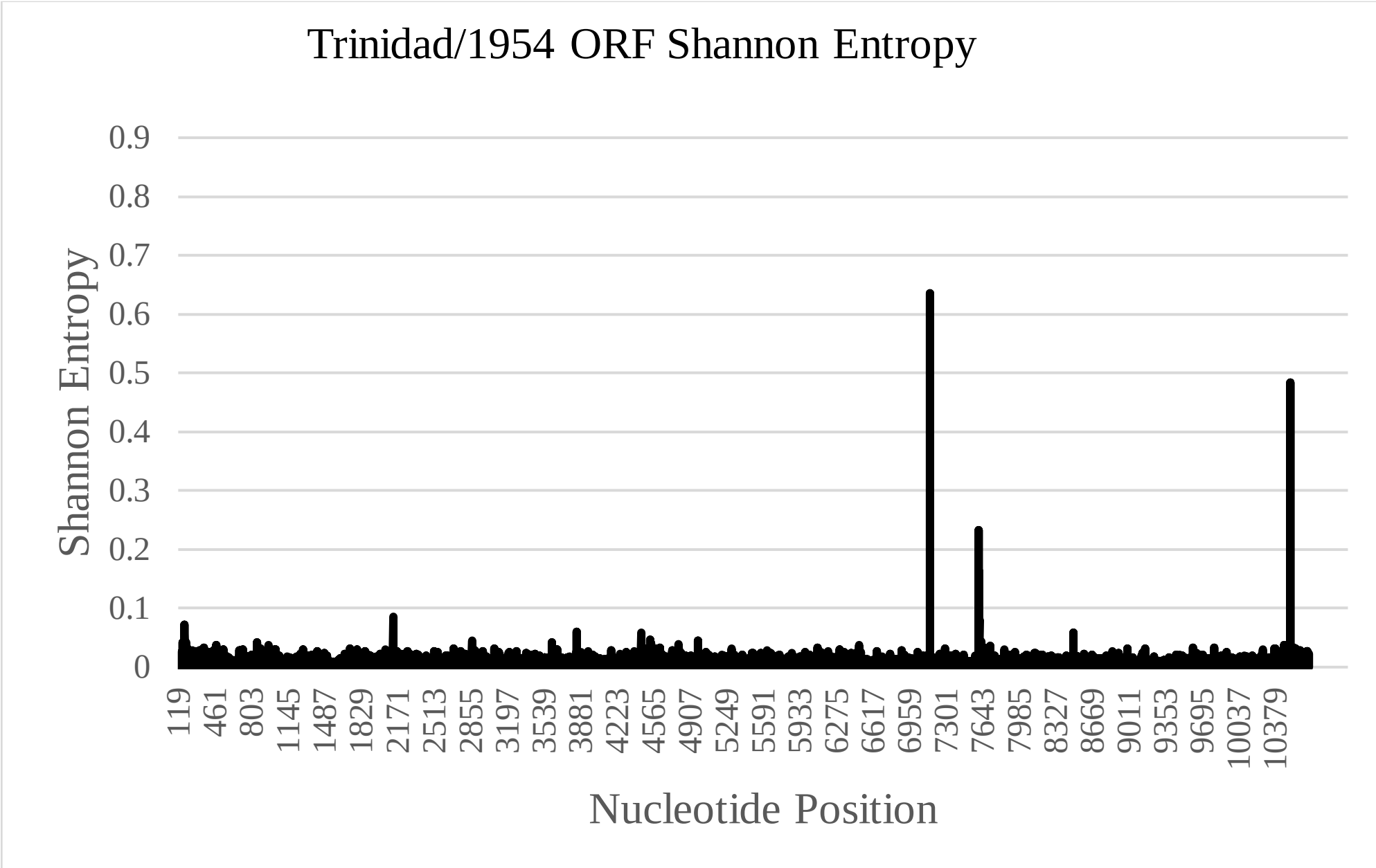
Trinidad/1954 ORF Shannon Entropy
Shannon Entropy
0.9
0.8
0.7
0.6
0.5
0.4
0.3
0.2
0.1
0
119
461
803
1145
1487
1829
2171
2513
2855
3197
3539
3881
4223
4565
4907
5249
5591
5933
6275
6617
6959
7301
7643
7985
8327
8669
9011
9353
9695
10037
10379
Nucleotide Position

F)

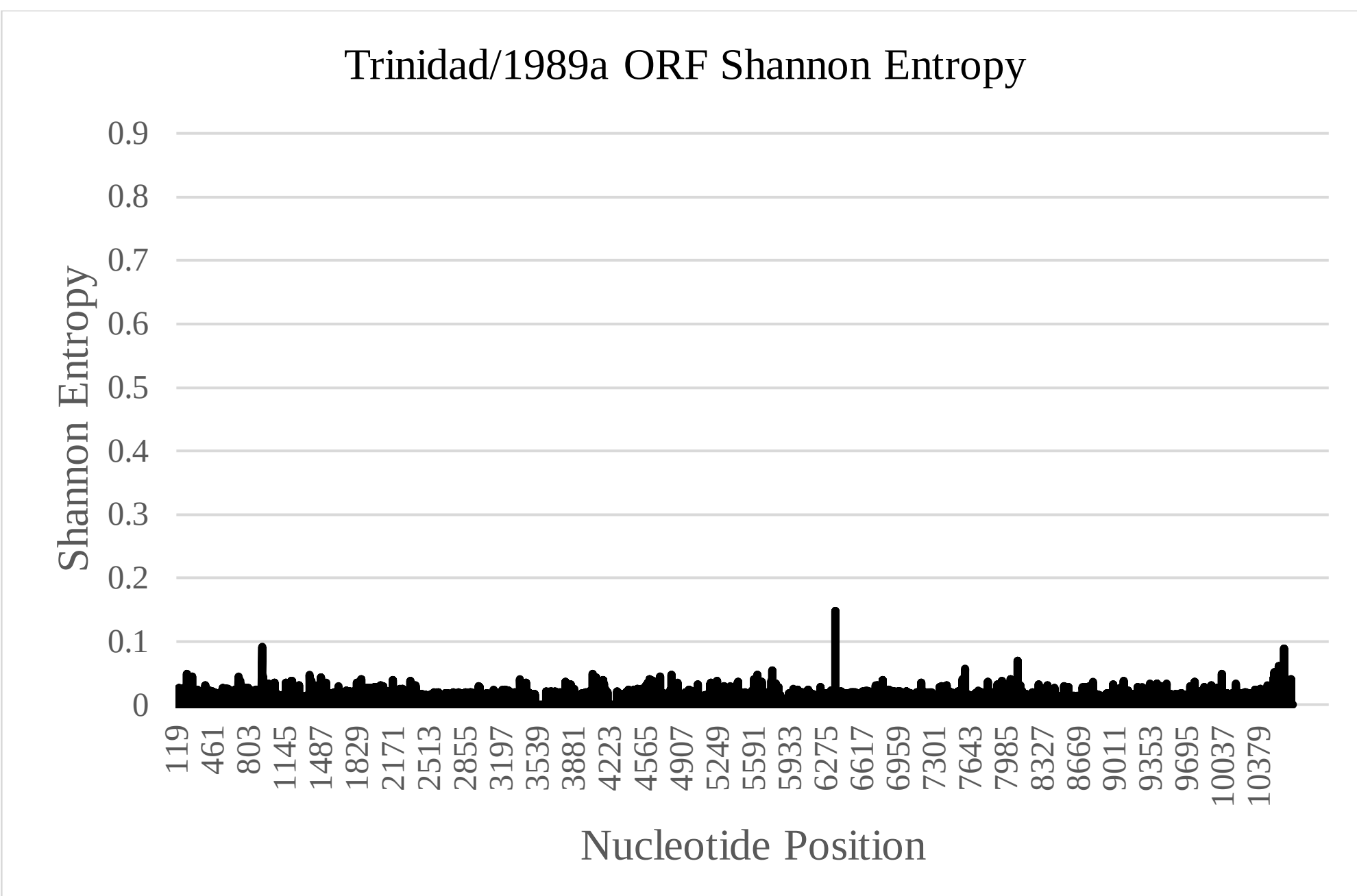
Trinidad/1989a ORF Shannon Entropy
Shannon Entropy
0.9
0.8
0.7
0.6
0.5
0.4
0.3
0.2
0.1
0
119
461
803
1145
1487
1829
2171
2513
2855
3197
3539
3881
4223
4565
4907
5249
5591
5933
6275
6617
6959
7301
7643
7985
8327
8669
9011
9353
9695
10037
10379
Nucleotide Position

G)

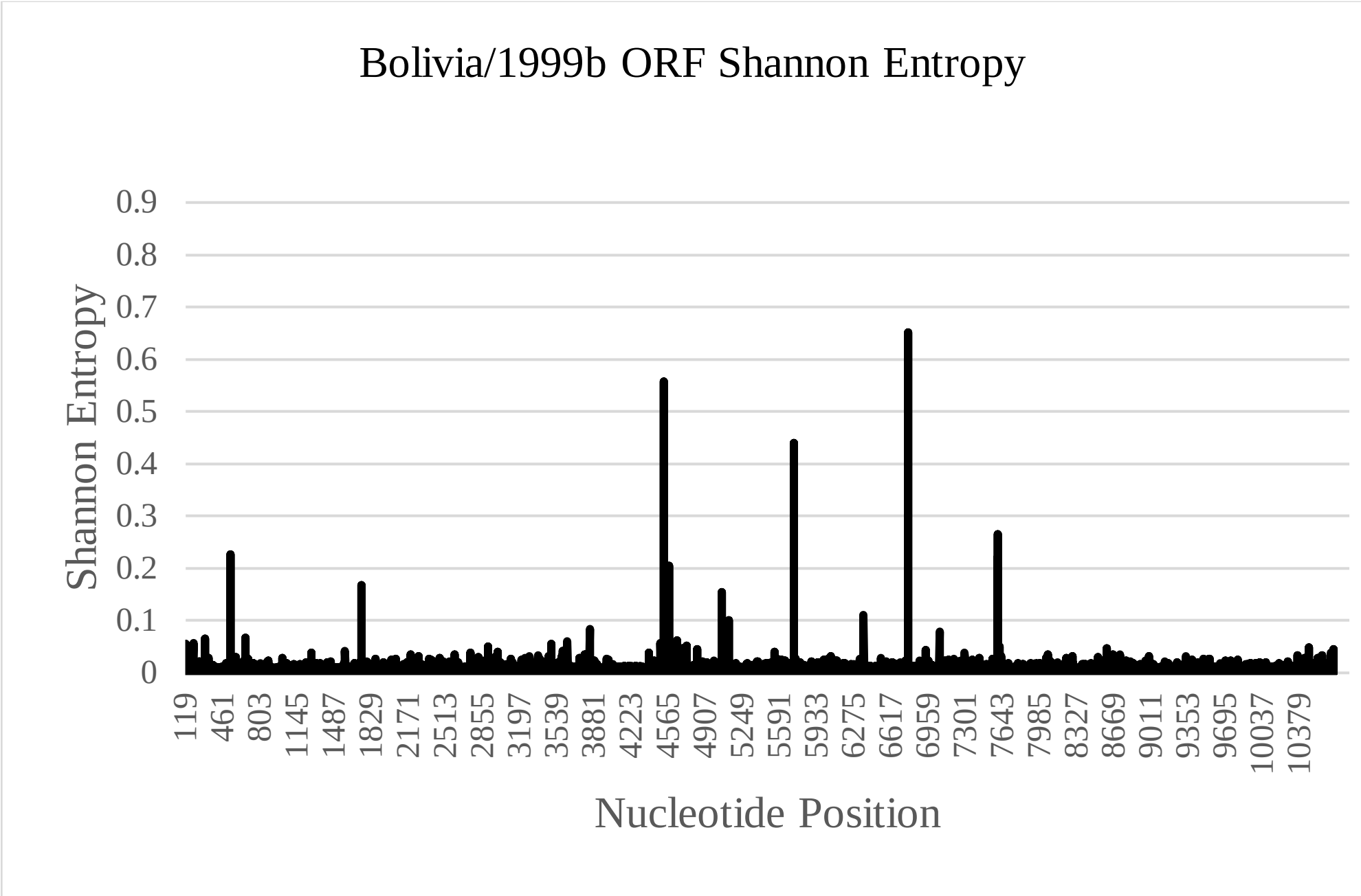
Bolivia/1999b ORF Shannon Entropy
Shannon Entropy
0.9
0.8
0.7
0.6
0.5
0.4
0.3
0.2
0.1
0
119
461
803
1145
1487
1829
2171
2513
2855
3197
3539
3881
4223
4565
4907
5249
5591
5933
6275
6617
6959
7301
7643
7985
8327
8669
9011
9353
9695
10037
10379
Nucleotide Position

H)

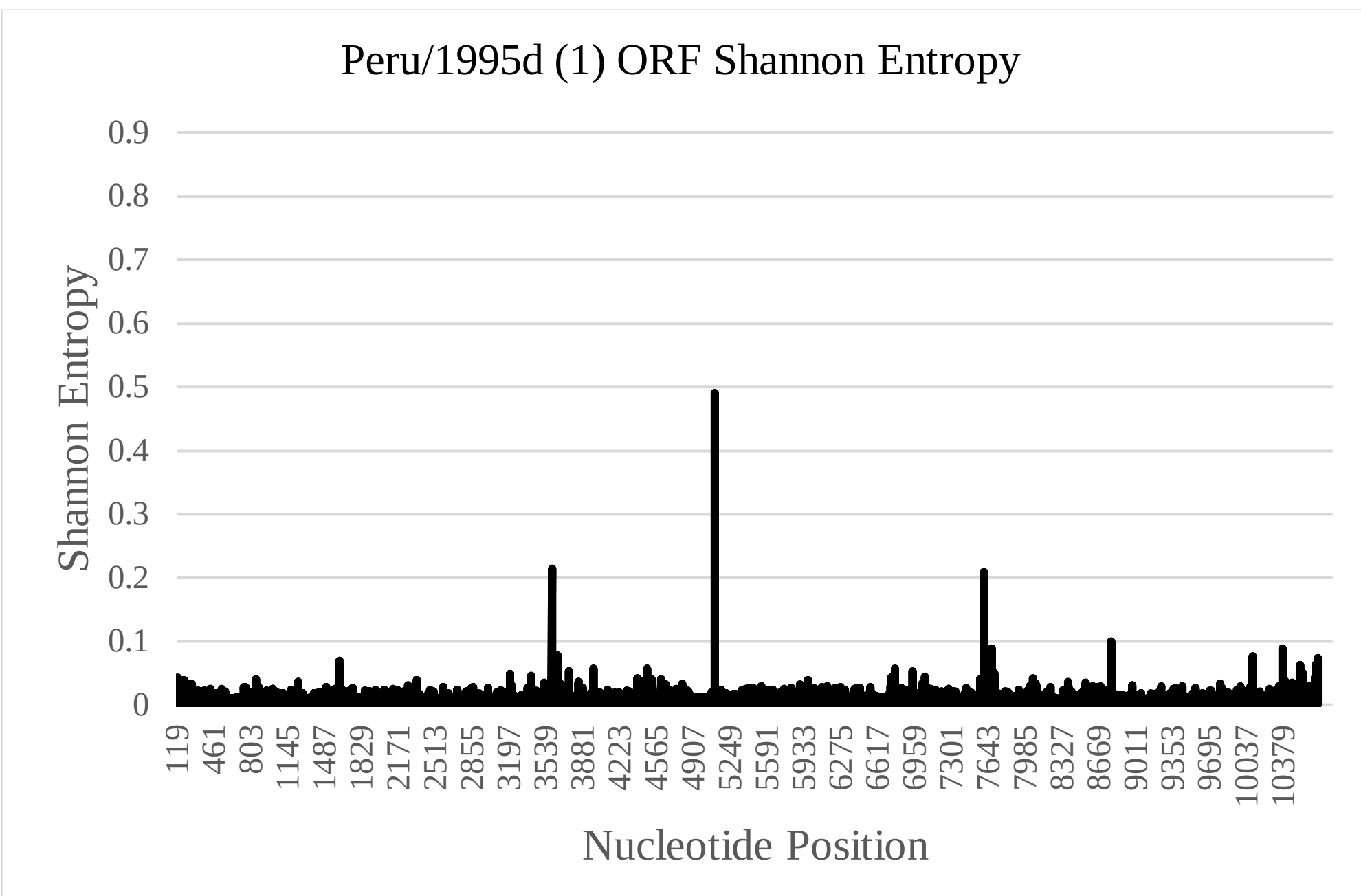
Peru/1995d (1) ORF Shannon Entropy
Shannon Entropy
0.9
0.8
0.7
0.6
0.5
0.4
0.3
0.2
0.1
0
119
461
803
1145
1487
1829
2171
2513
2855
3197
3539
3881
4223
4565
4907
5249
5591
5933
6275
6617
6959
7301
7643
7985
8327
8669
9011
9353
9695
10037
10379
Nucleotide Position

I)

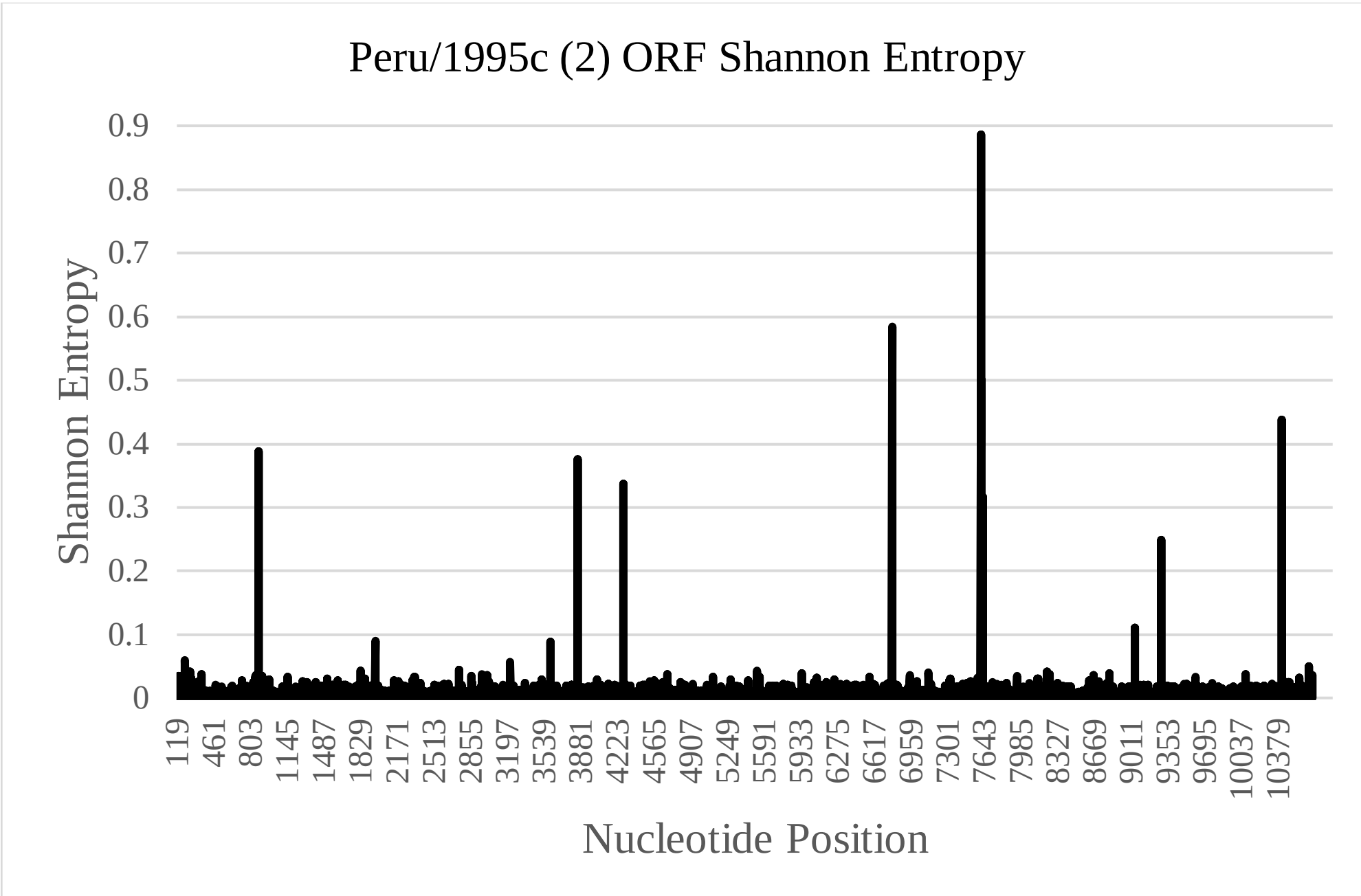

Peru/1995c (2) ORF Shannon Entropy
0.9
0.8
0.7
0.6
0.5
0.4
0.3
0.2
0.1
0
Shannon Entropy
119
461
803
1145
1487
1829
2171
2513
2855
3197
3539
3881
4223
4565
4907
5249
5591
5933
6275
6617
6959
7301
7643
7985
8327
8669
9011
9353
9695
10037
10379
Nucleotide Position

J)

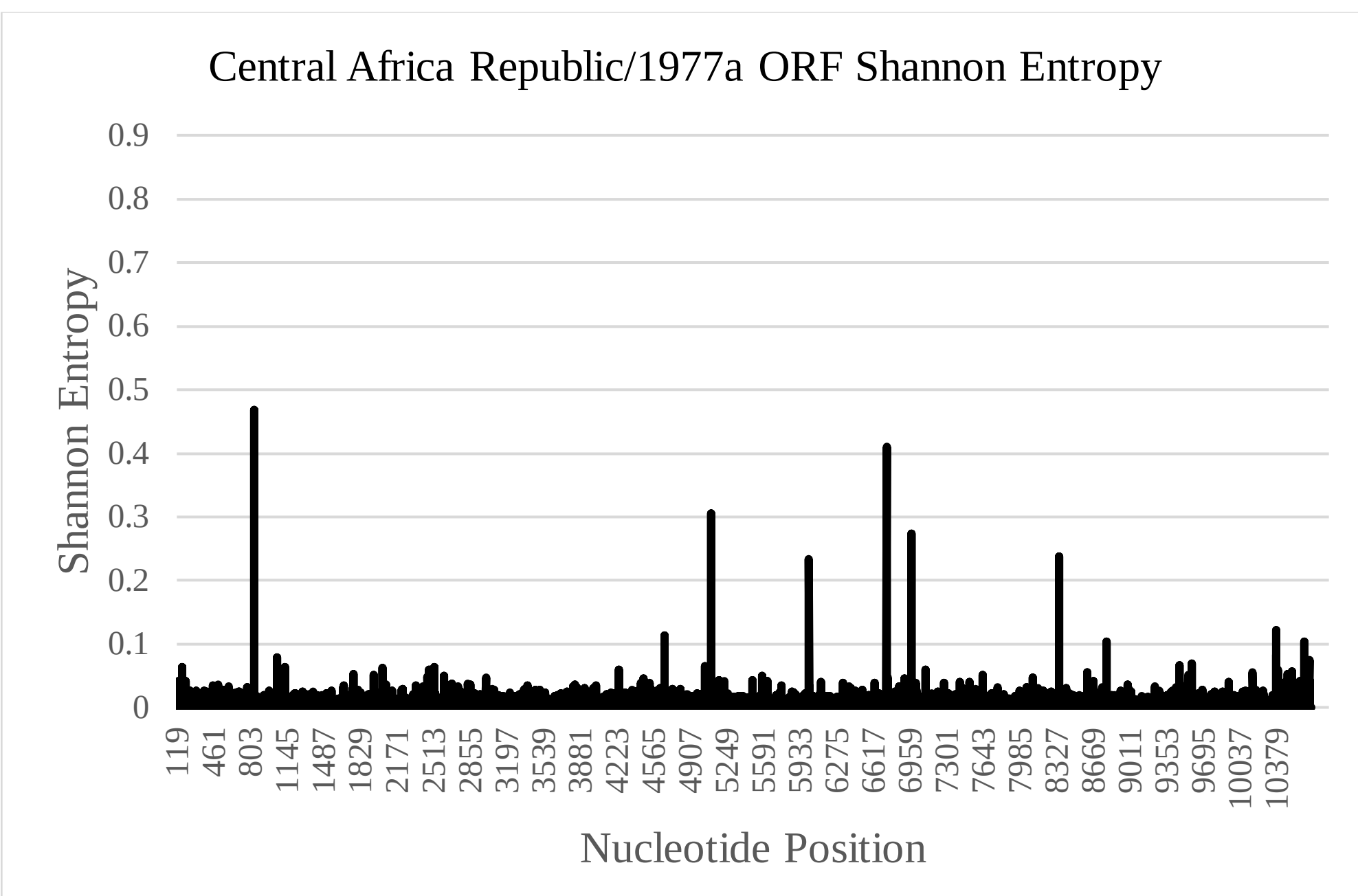

Central Africa Republic/1977a ORF Shannon Entropy
0.9
0.8
0.7
0.6
0.5
0.4
0.3
0.2
0.1
0
Shannon Entropy
119
461
803
1145
1487
1829
2171
2513
2855
3197
3539
3881
4223
4565
4907
5249
5591
5933
6275
6617
6959
7301
7643
7985
8327
8669
9011
9353
9695
10037
10379
Nucleotide Position

K)

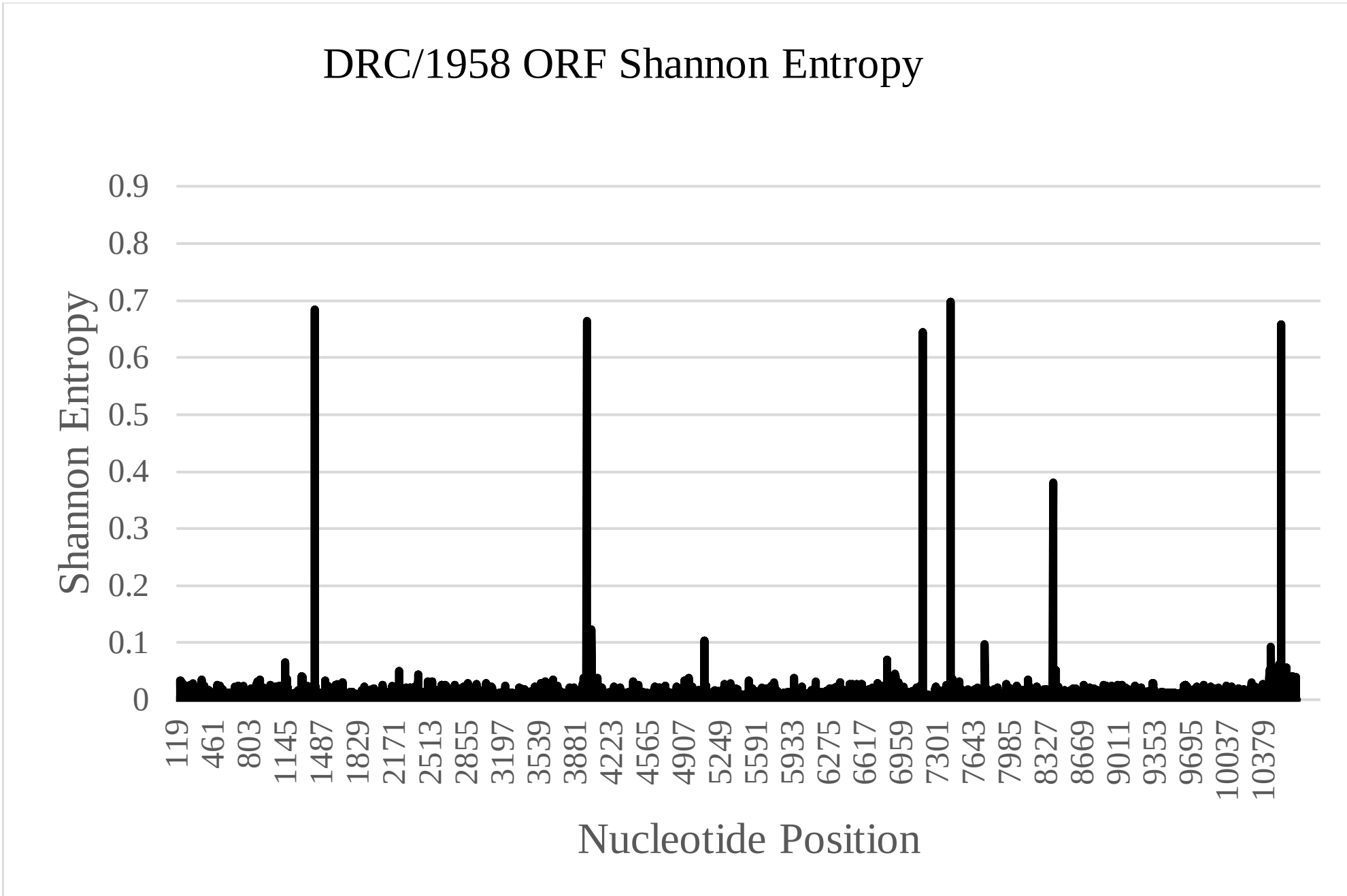
DRC/1958 ORF Shannon Entropy
Shannon Entropy
0.9
0.8
0.7
0.6
0.5
0.4
0.3
0.2
0.1
0
119
461
803
1145
1487
1829
2171
2513
2855
3197
3539
3881
4223
4565
4907
5249
5591
5933
6275
6617
6959
7301
7643
7985
8327
8669
9011
9353
9695
10037
10379
Nucleotide Position

L)

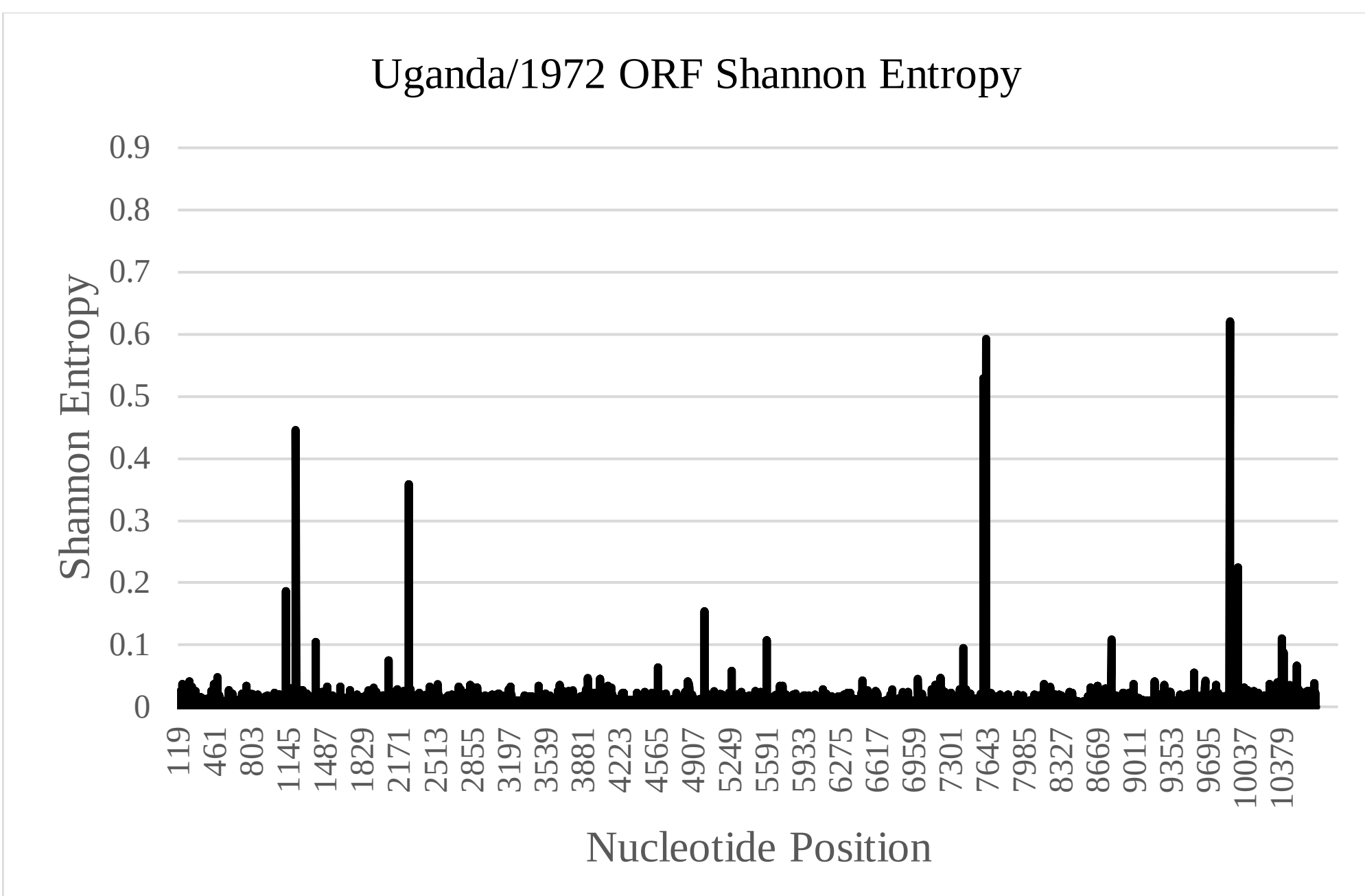
Uganda/1972 ORF Shannon Entropy
Shannon Entropy
0.9
0.8
0.7
0.6
0.5
0.4
0.3
0.2
0.1
0
119
461
803
1145
1487
1829
2171
2513
2855
3197
3539
3881
4223
4565
4907
5249
5591
5933
6275
6617
6959
7301
7643
7985
8327
8669
9011
9353
9695
10037
10379
Nucleotide Position

M)

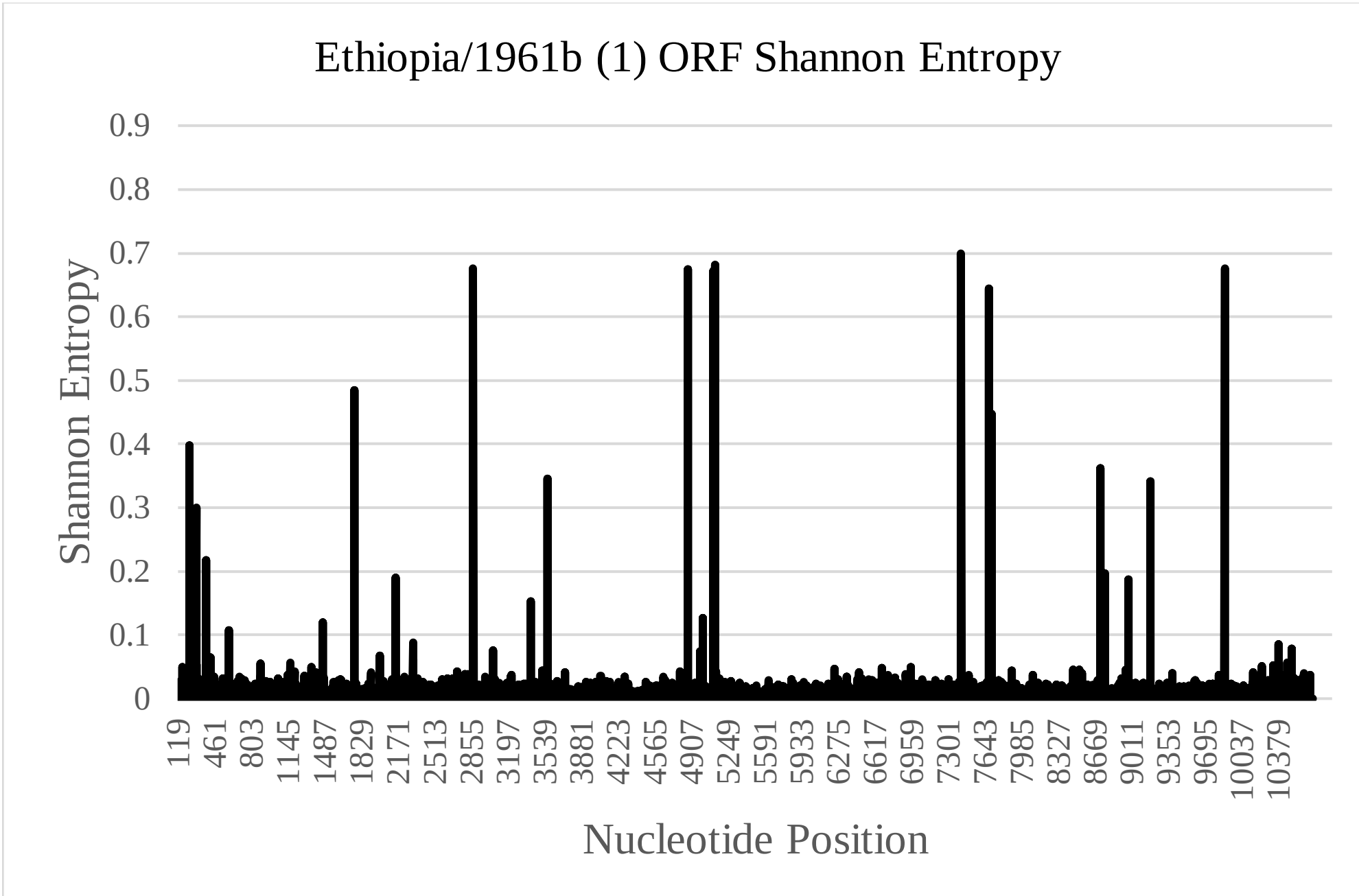

Ethiopia/1961b (1) ORF Shannon Entropy
Shannon Entropy
0.9
0.8
0.7
0.6
0.5
0.4
0.3
0.2
0.1
0
119
461
803
1145
1487
1829
2171
2513
2855
3197
3539
3881
4223
4565
4907
5249
5591
5933
6275
6617
6959
7301
7643
7985
8327
8669
9011
9353
9695
10037
10379
Nucleotide Position

N)

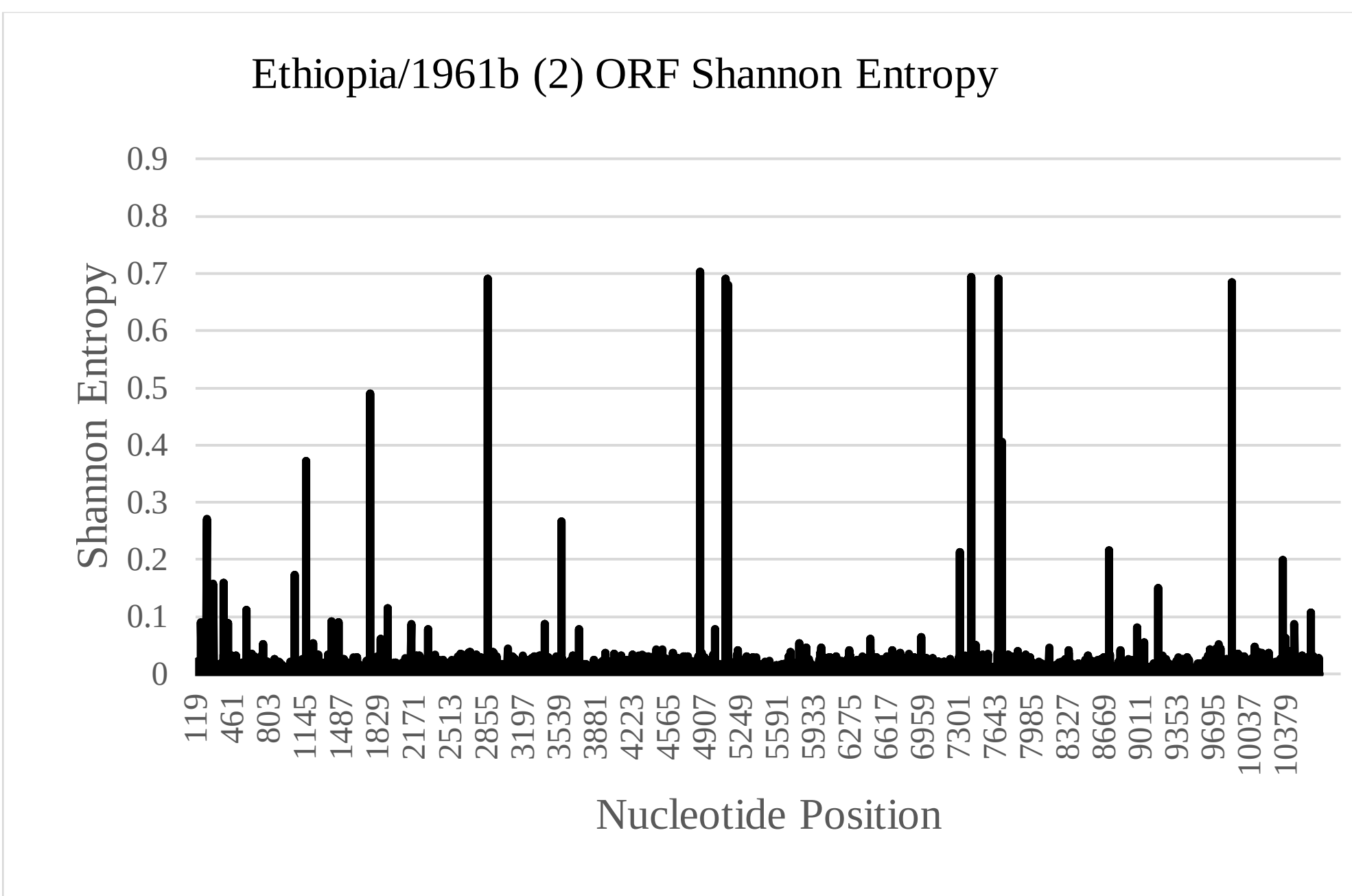

Ethiopia/1961b (2) ORF Shannon Entropy
Shannon Entropy
0.9
0.8
0.7
0.6
0.5
0.4
0.3
0.2
0.1
0
119
461
803
1145
1487
1829
2171
2513
2855
3197
3539
3881
4223
4565
4907
5249
5591
5933
6275
6617
6959
7301
7643
7985
8327
8669
9011
9353
9695
10037
10379
Nucleotide Position

O)

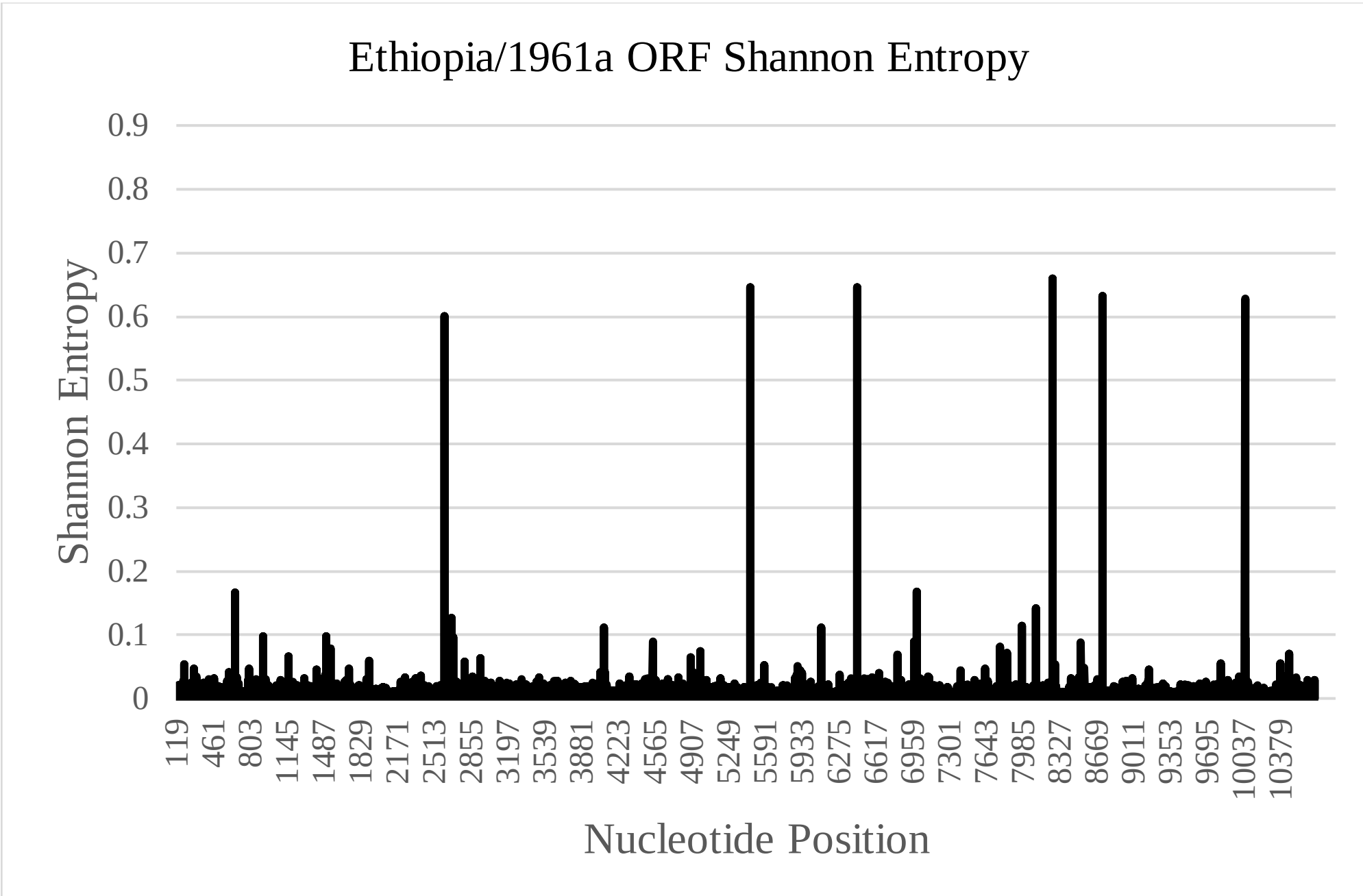

Ethiopia/1961a ORF Shannon Entropy
Shannon Entropy
0.9
0.8
0.7
0.6
0.5
0.4
0.3
0.2
0.1
0
119
461
803
1145
1487
1829
2171
2513
2855
3197
3539
3881
4223
4565
4907
5249
5591
5933
6275
6617
6959
7301
7643
7985
8327
8669
9011
9353
9695
10037
10379
Nucleotide Position

P)

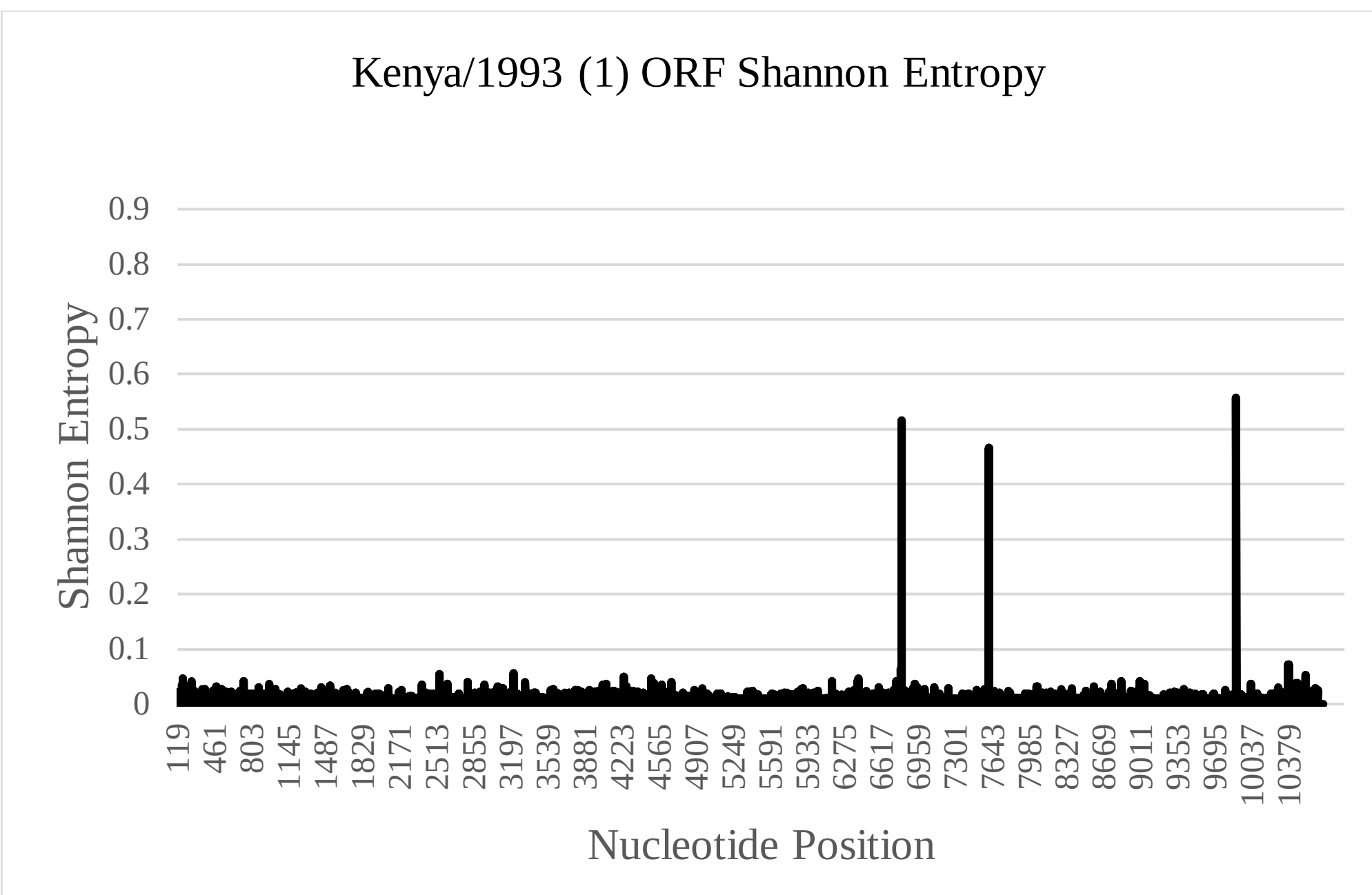

Kenya/1993 (1) ORF Shannon Entropy
Shannon Entropy
0.9
0.8
0.7
0.6
0.5
0.4
0.3
0.2
0.1
0
119
461
803
1145
1487
1829
2171
2513
2855
3197
3539
3881
4223
4565
4907
5249
5591
5933
6275
6617
6959
7301
7643
7985
8327
8669
9011
9353
9695
10037
10379
Nucleotide Position

Q)

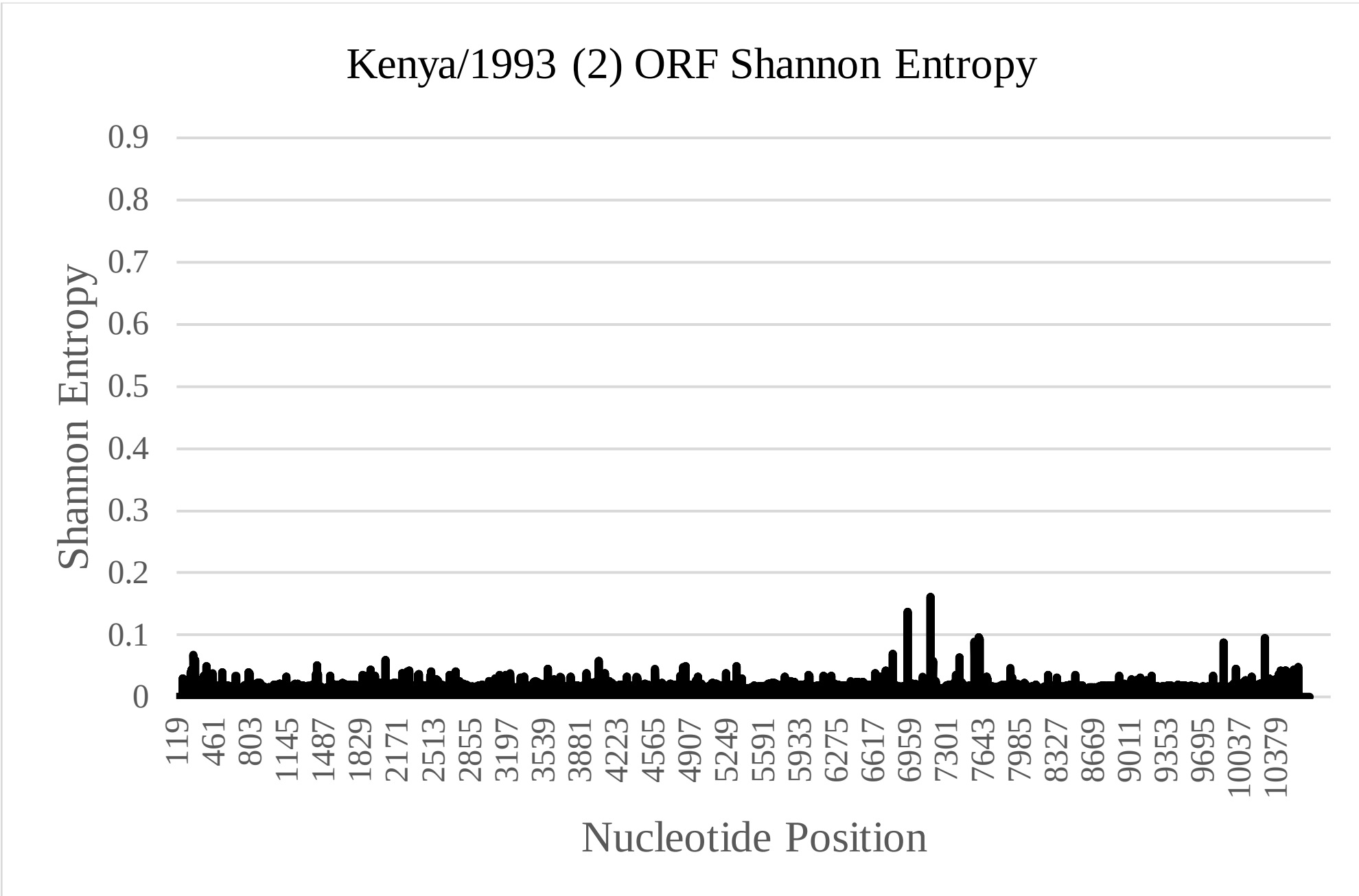
Kenya/1993 (2) ORF Shannon Entropy
Shannon Entropy
0.9
0.8
0.7
0.6
0.5
0.4
0.3
0.2
0.1
0
119
461
803
1145
1487
1829
2171
2513
2855
3197
3539
3881
4223
4565
4907
5249
5591
5933
6275
6617
6959
7301
7643
7985
8327
8669
9011
9353
9695
10037
10379
Nucleotide Position

R)

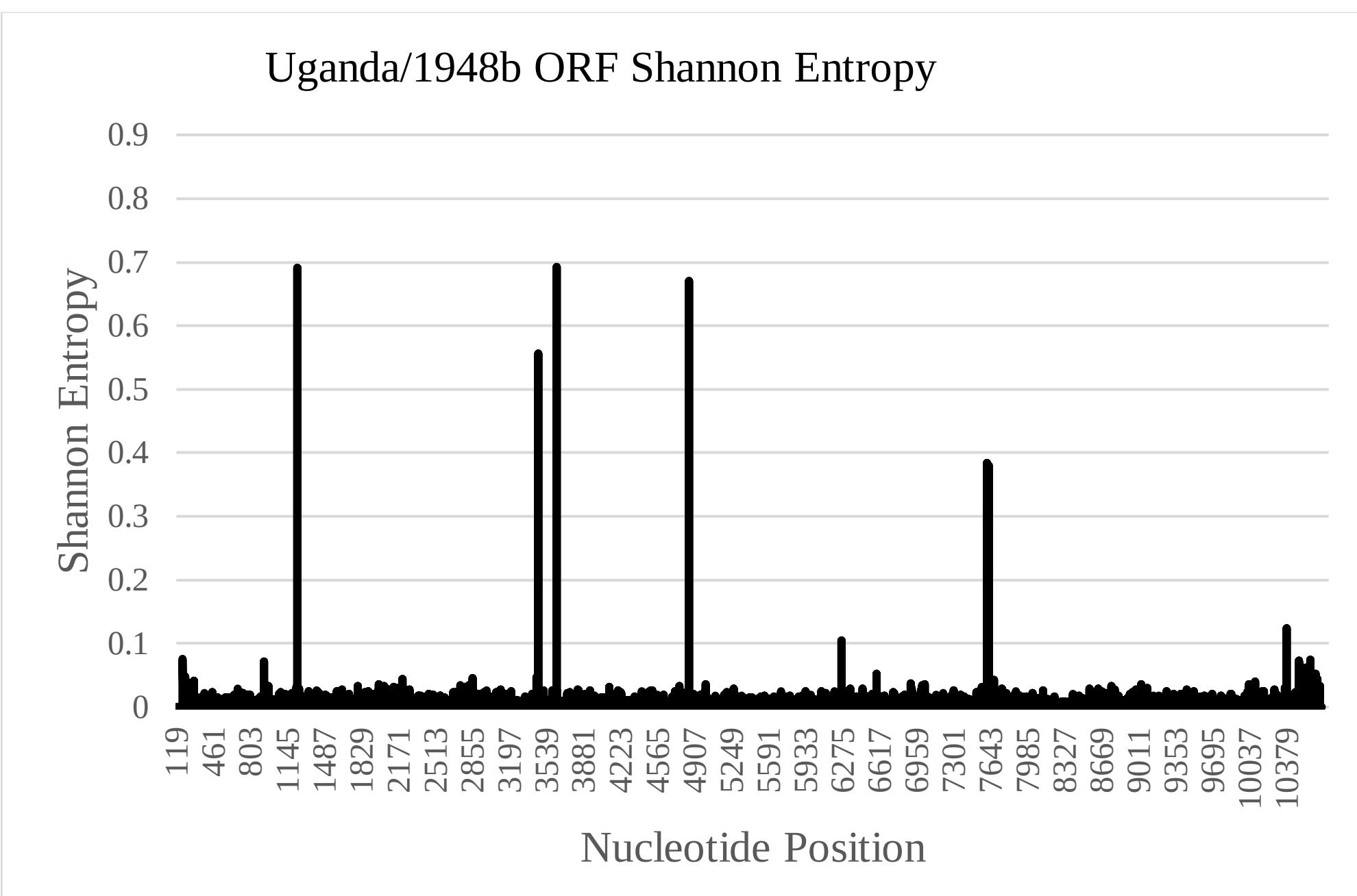
Uganda/1948b ORF Shannon Entropy
Shannon Entropy
0.9
0.8
0.7
0.6
0.5
0.4
0.3
0.2
0.1
0
119
461
803
1145
1487
1829
2171
2513
2855
3197
3539
3881
4223
4565
4907
5249
5591
5933
6275
6617
6959
7301
7643
7985
8327
8669
9011
9353
9695
10037
10379
Nucleotide Position

S)

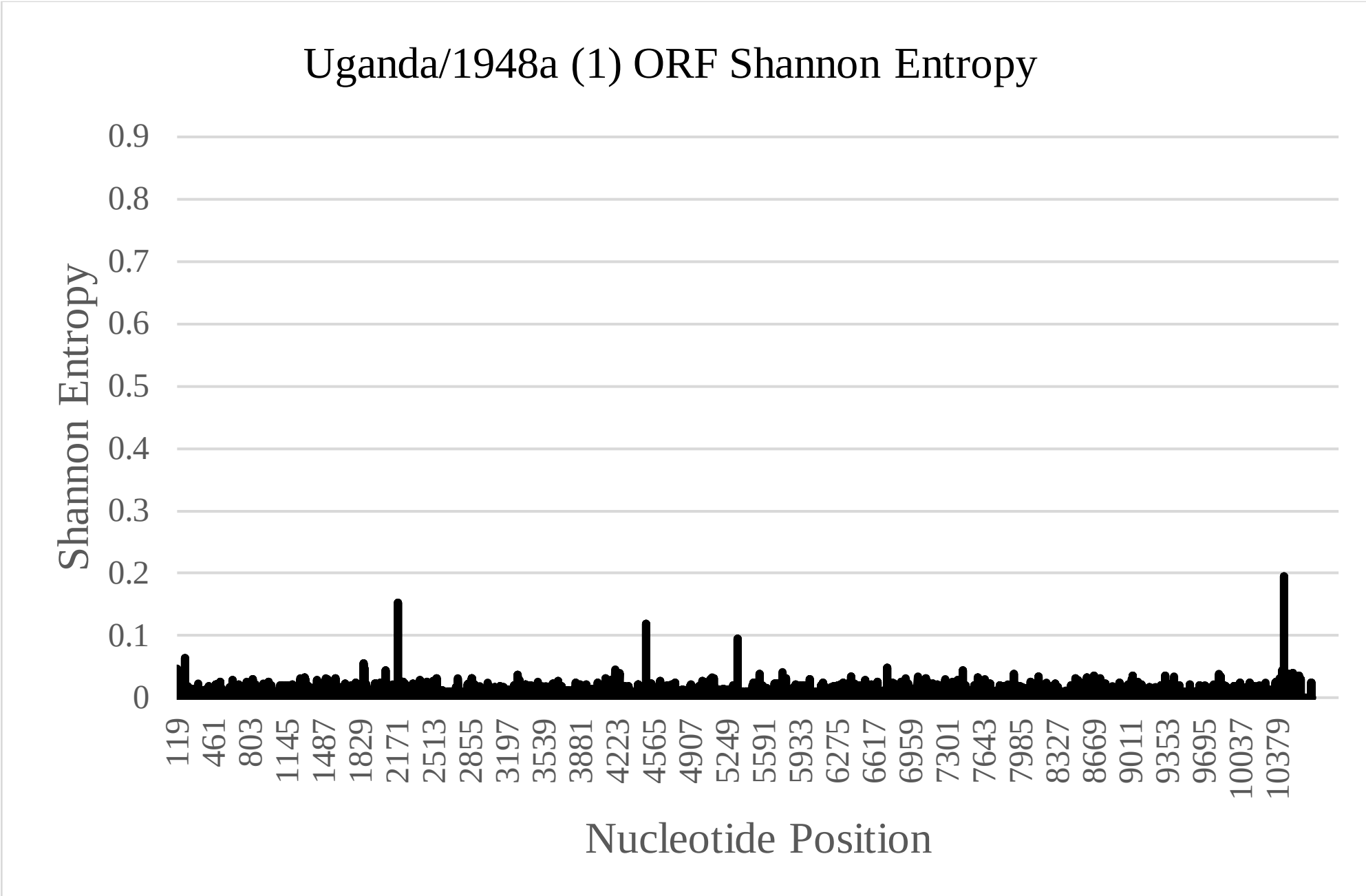

Uganda/1948a (1) ORF Shannon Entropy
0.9
0.8
0.7
0.6
0.5
0.4
0.3
0.2
0.1
0
Shannon Entropy
119
461
803
1145
1487
1829
2171
2513
2855
3197
3539
3881
4223
4565
4907
5249
5591
5933
6275
6617
6959
7301
7643
7985
8327
8669
9011
9353
9695
10037
10379
Nucleotide Position

T)

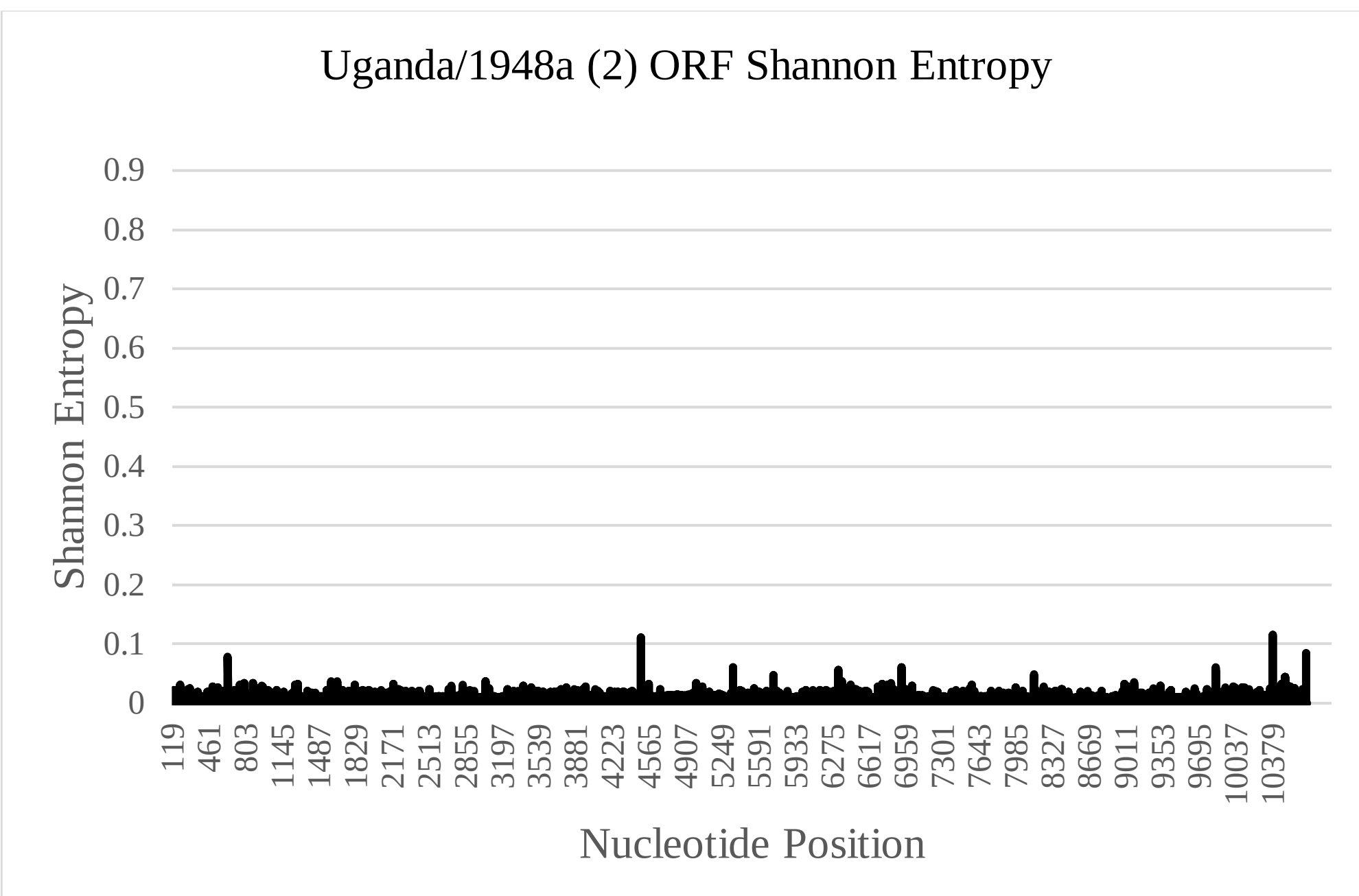

Uganda/1948a (2) ORF Shannon Entropy
0.9
0.8
0.7
0.6
0.5
0.4
0.3
0.2
0.1
0
Shannon Entropy
119
461
803
1145
1487
1829
2171
2513
2855
3197
3539
3881
4223
4565
4907
5249
5591
5933
6275
6617
6959
7301
7643
7985
8327
8669
9011
9353
9695
10037
10379
Nucleotide Position

U)

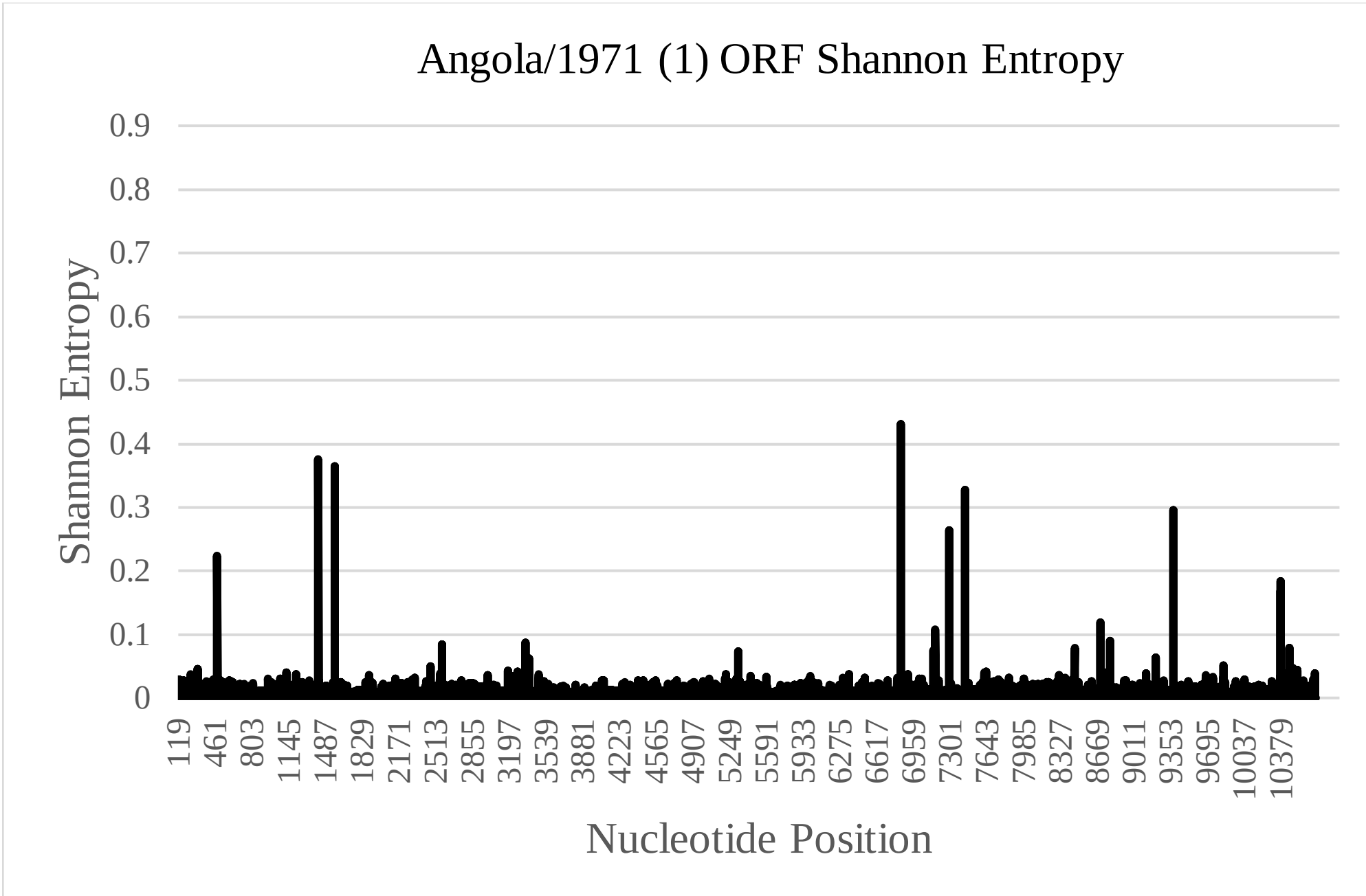

Angola/1971 (1) ORF Shannon Entropy
Shannon Entropy
0.9
0.8
0.7
0.6
0.5
0.4
0.3
0.2
0.1
0
119
461
803
1145
1487
1829
2171
2513
2855
3197
3539
3881
4223
4565
4907
5249
5591
5933
6275
6617
6959
7301
7643
7985
8327
8669
9011
9353
9695
10037
10379
Nucleotide Position

V)

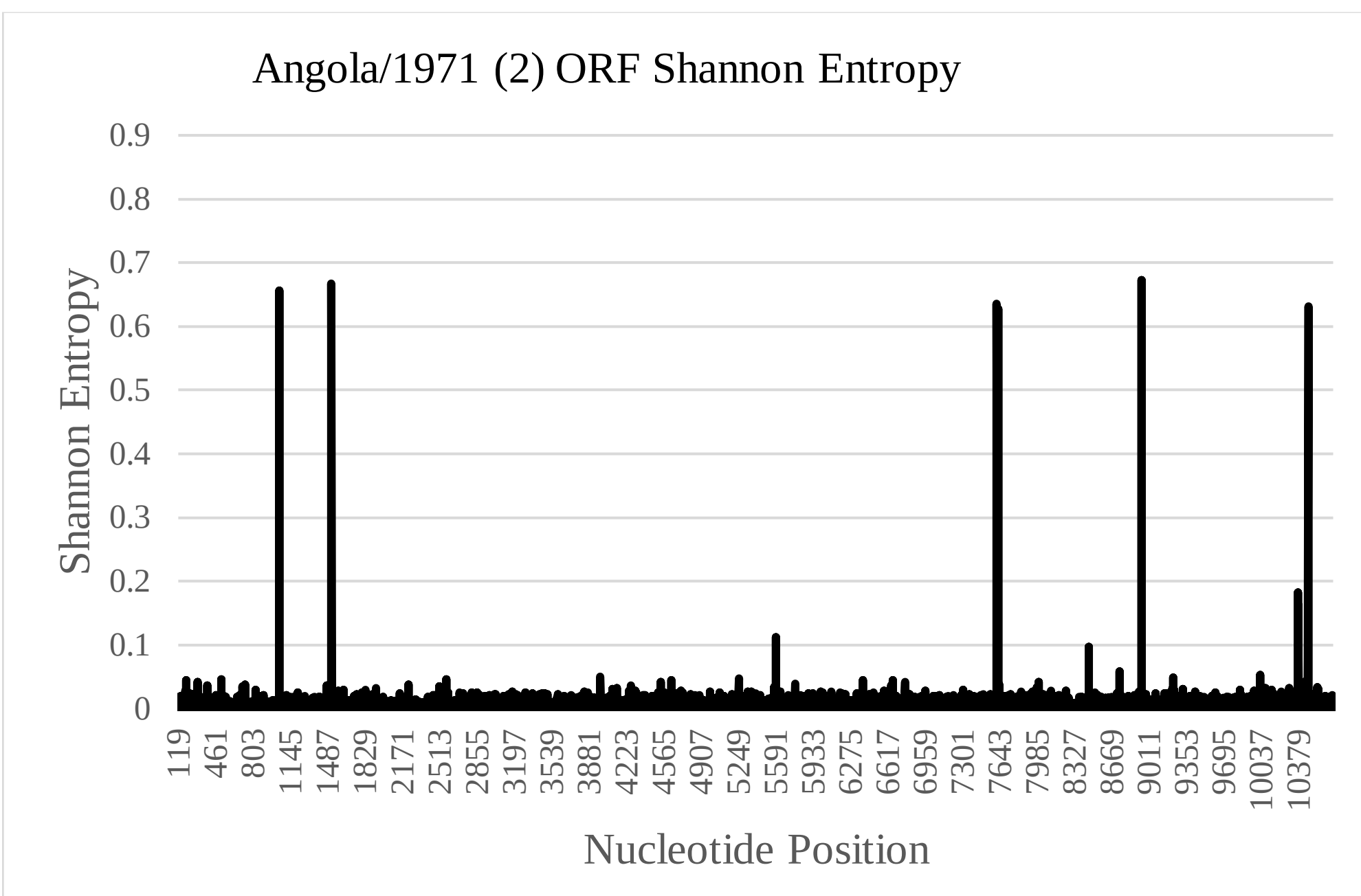

Angola/1971 (2) ORF Shannon Entropy
Shannon Entropy
0.9
0.8
0.7
0.6
0.5
0.4
0.3
0.2
0.1
0
119
461
803
1145
1487
1829
2171
2513
2855
3197
3539
3881
4223
4565
4907
5249
5591
5933
6275
6617
6959
7301
7643
7985
8327
8669
9011
9353
9695
10037
10379
Nucleotide Position

W)

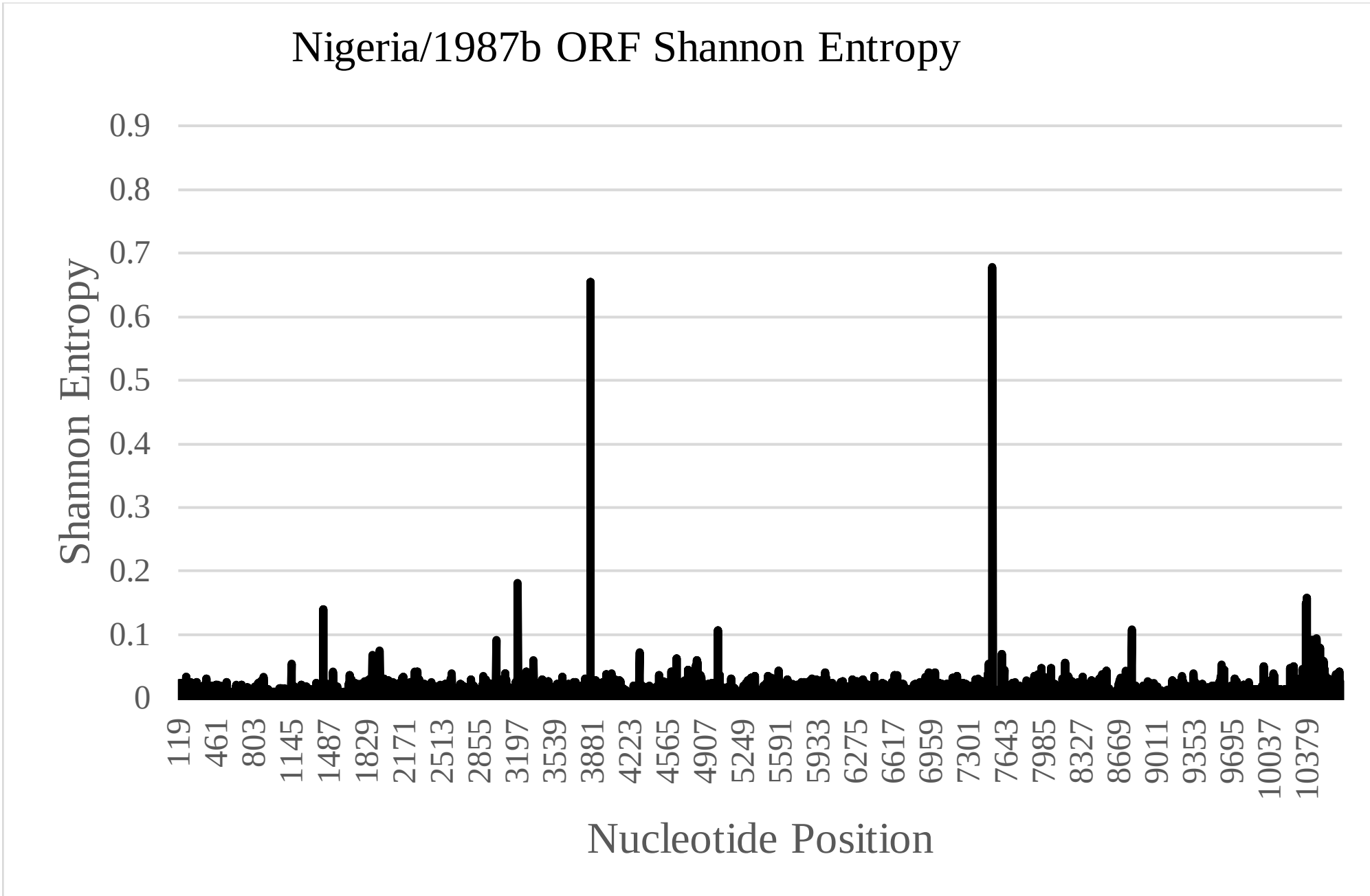
Nigeria/1987b ORF Shannon Entropy
Shannon Entropy
0.9
0.8
0.7
0.6
0.5
0.4
0.3
0.2
0.1
0
119
461
803
1145
1487
1829
2171
2513
2855
3197
3539
3881
4223
4565
4907
5249
5591
5933
6275
6617
6959
7301
7643
7985
8327
8669
9011
9353
9695
10037
10379
Nucleotide Position

X)

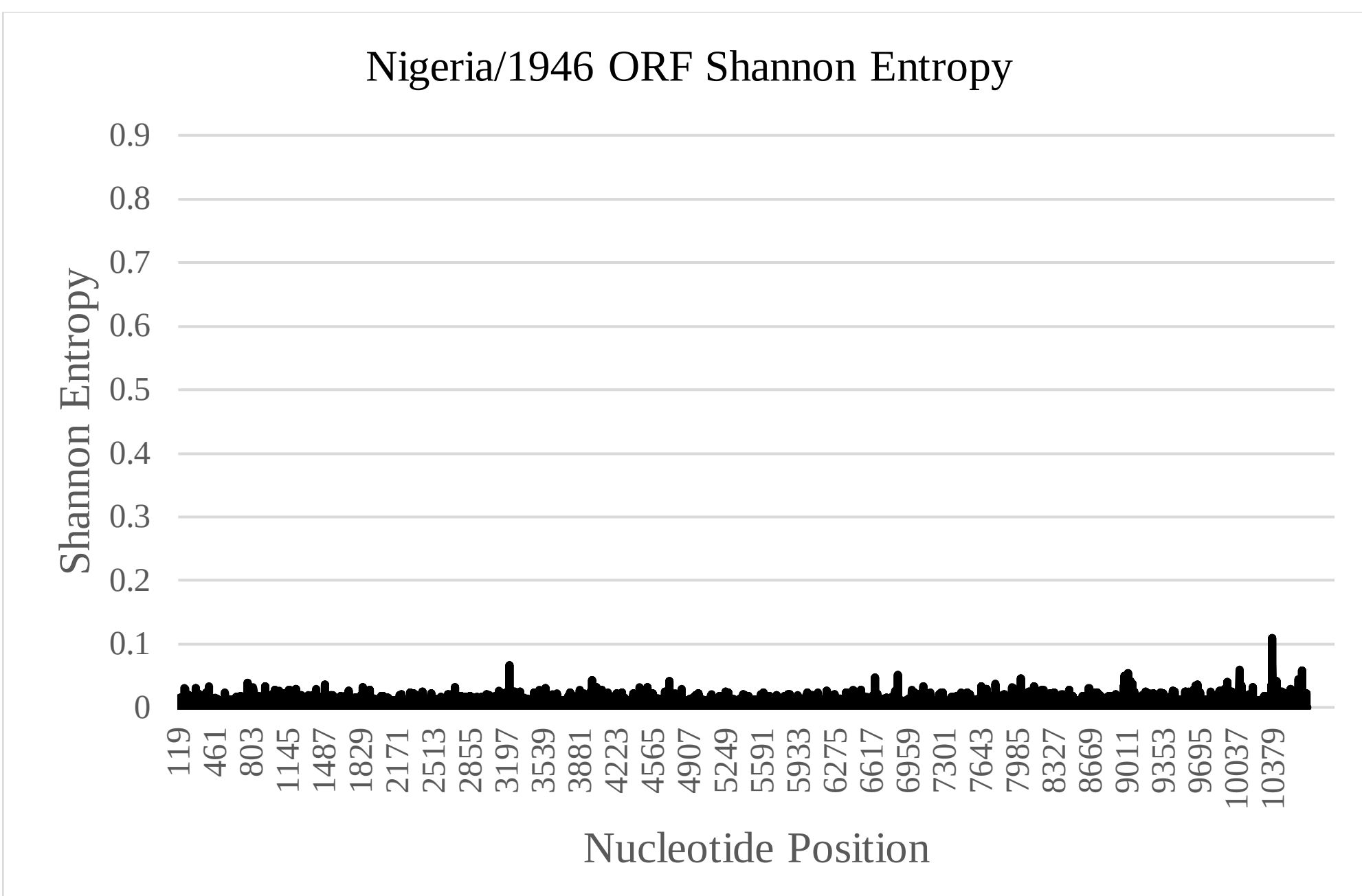
Nigeria/1946 ORF Shannon Entropy
Shannon Entropy
0.9
0.8
0.7
0.6
0.5
0.4
0.3
0.2
0.1
0
119
461
803
1145
1487
1829
2171
2513
2855
3197
3539
3881
4223
4565
4907
5249
5591
5933
6275
6617
6959
7301
7643
7985
8327
8669
9011
9353
9695
10037
10379
Nucleotide Position

Y)

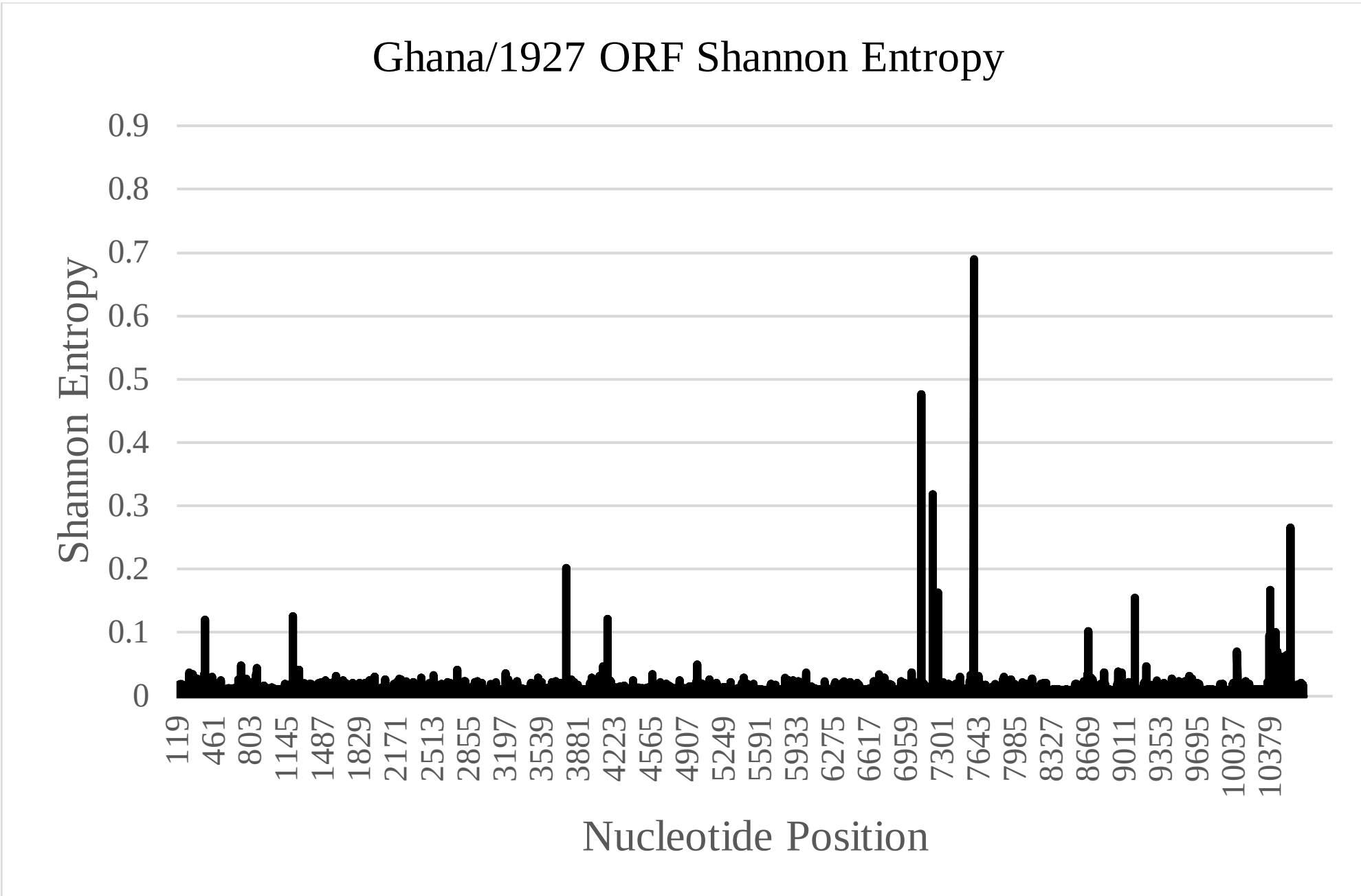

Ghana/1927 ORF Shannon Entropy
Shannon Entropy
0.9
0.8
0.7
0.6
0.5
0.4
0.3
0.2
0.1
0
119
461
803
1145
1487
1829
2171
2513
2855
3197
3539
3881
4223
4565
4907
5249
5591
5933
6275
6617
6959
7301
7643
7985
8327
8669
9011
9353
9695
10037
10379
Nucleotide Position

Supplementary Figure 2: SNV graphs for each virus preparation used in our study.

A)

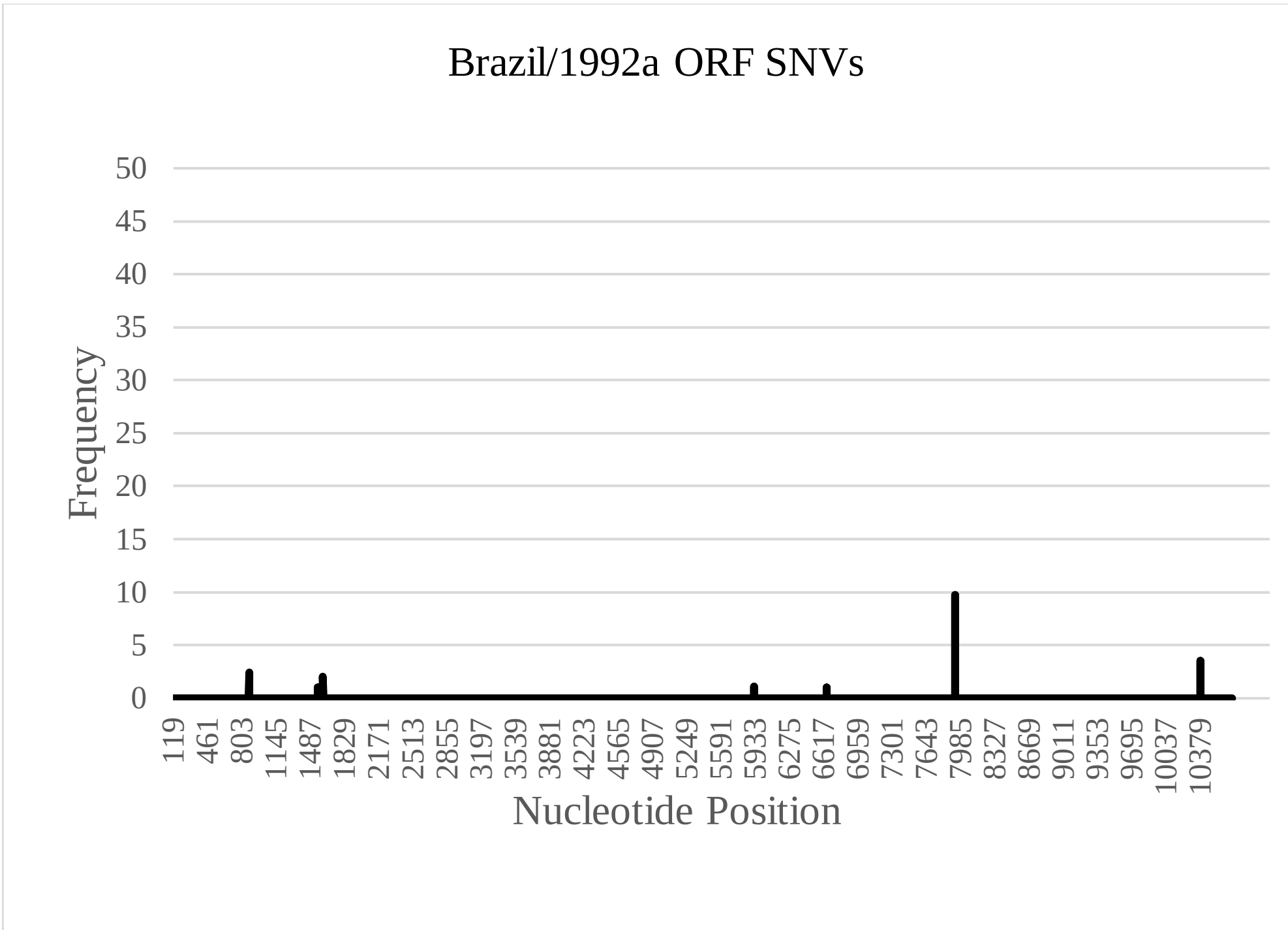

B)

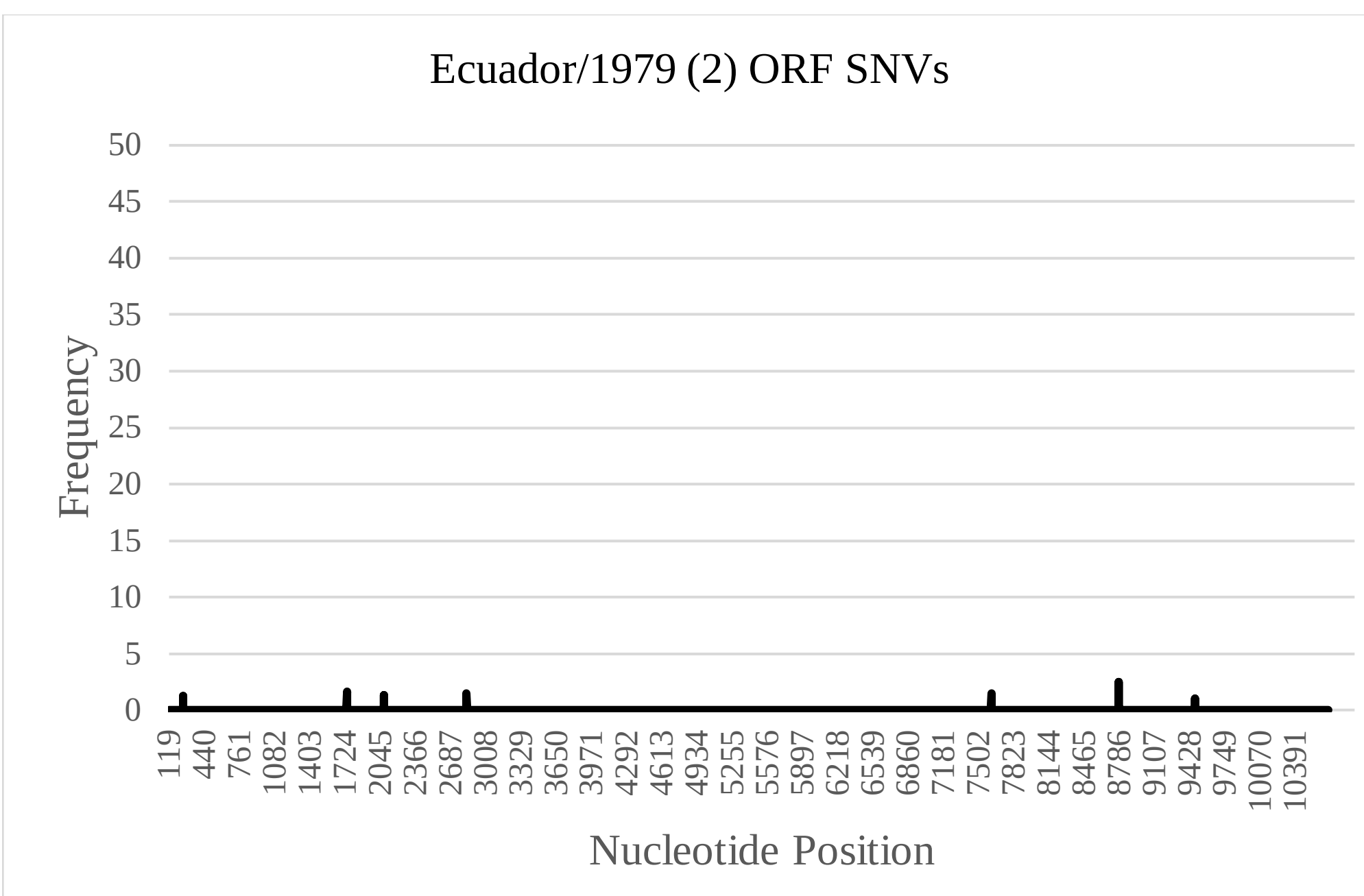

C)

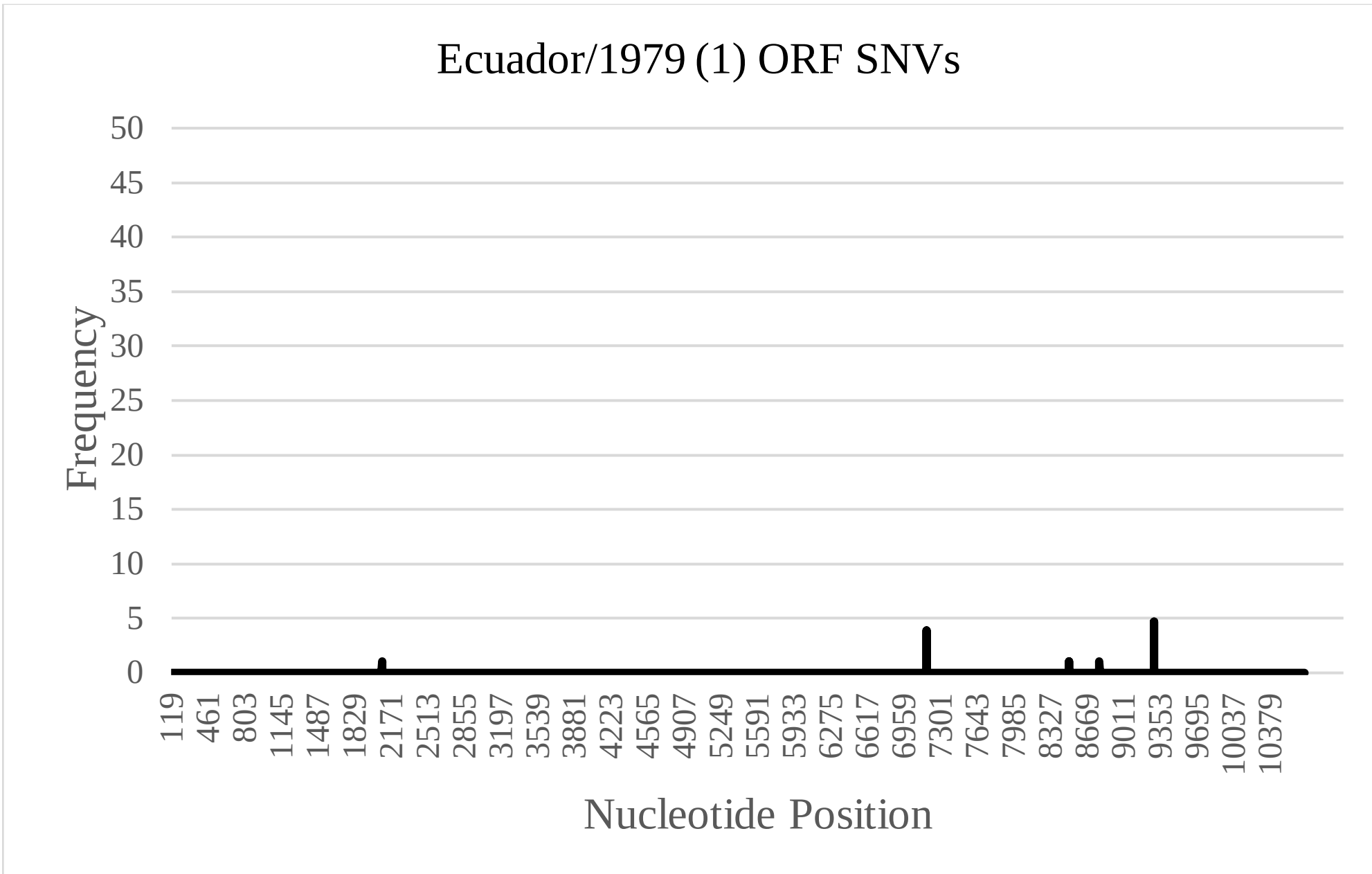
Ecuador/1979 (1) ORF SNVs
Frequency
50
45
40
35
30
25
20
15
10
5
0
119
461
803
1145
1487
1829
2171
2513
2855
3197
3539
3881
4223
4565
4907
5249
5591
5933
6275
6617
6959
7301
7643
7985
8327
8669
9011
9353
9695
10037
10379
Nucleotide Position

D)

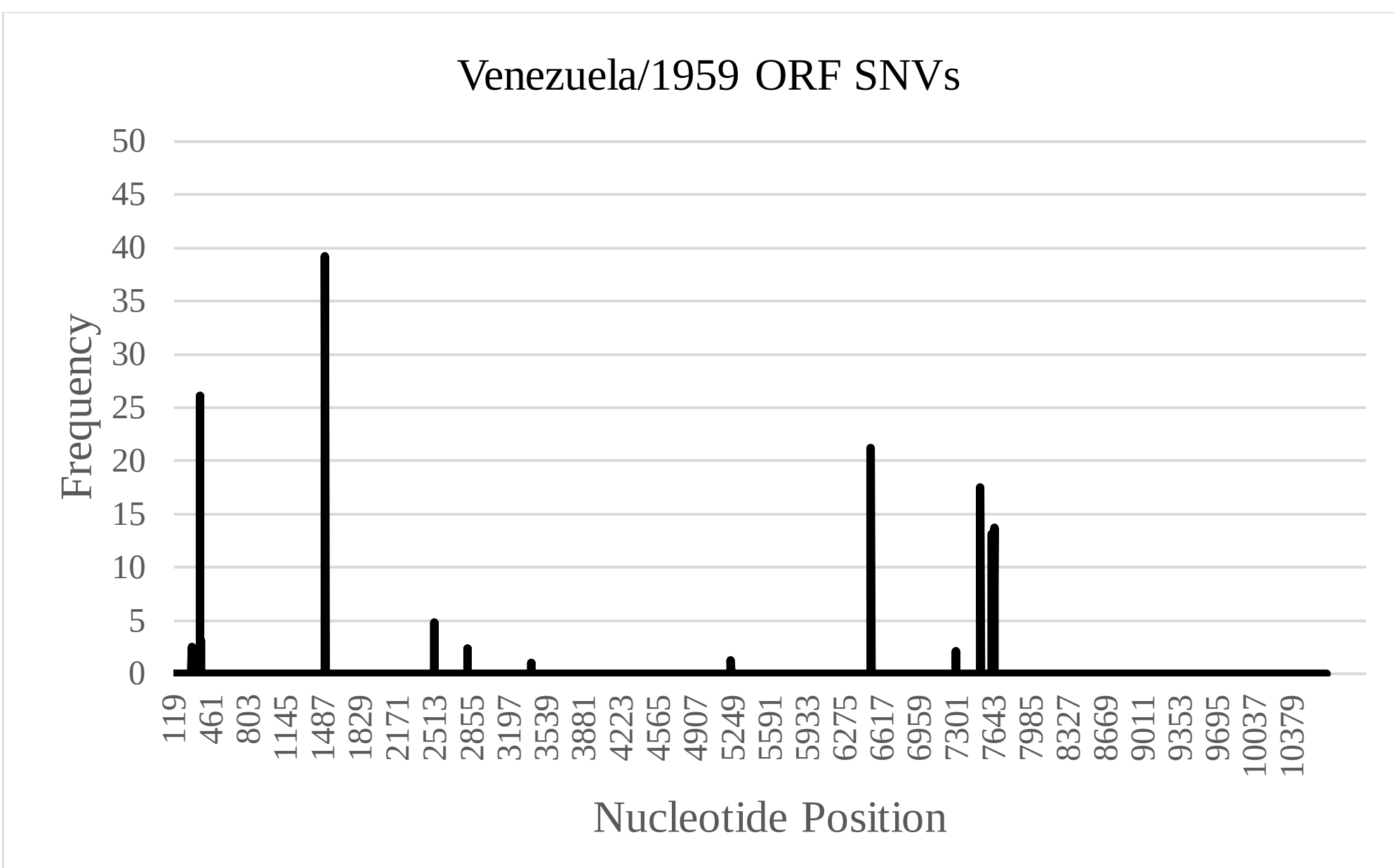
Venezuela/1959 ORF SNVs
Frequency
50
45
40
35
30
25
20
15
10
5
0
119
461
803
1145
1487
1829
2171
2513
2855
3197
3539
3881
4223
4565
4907
5249
5591
5933
6275
6617
6959
7301
7643
7985
8327
8669
9011
9353
9695
10037
10379
Nucleotide Position

E)

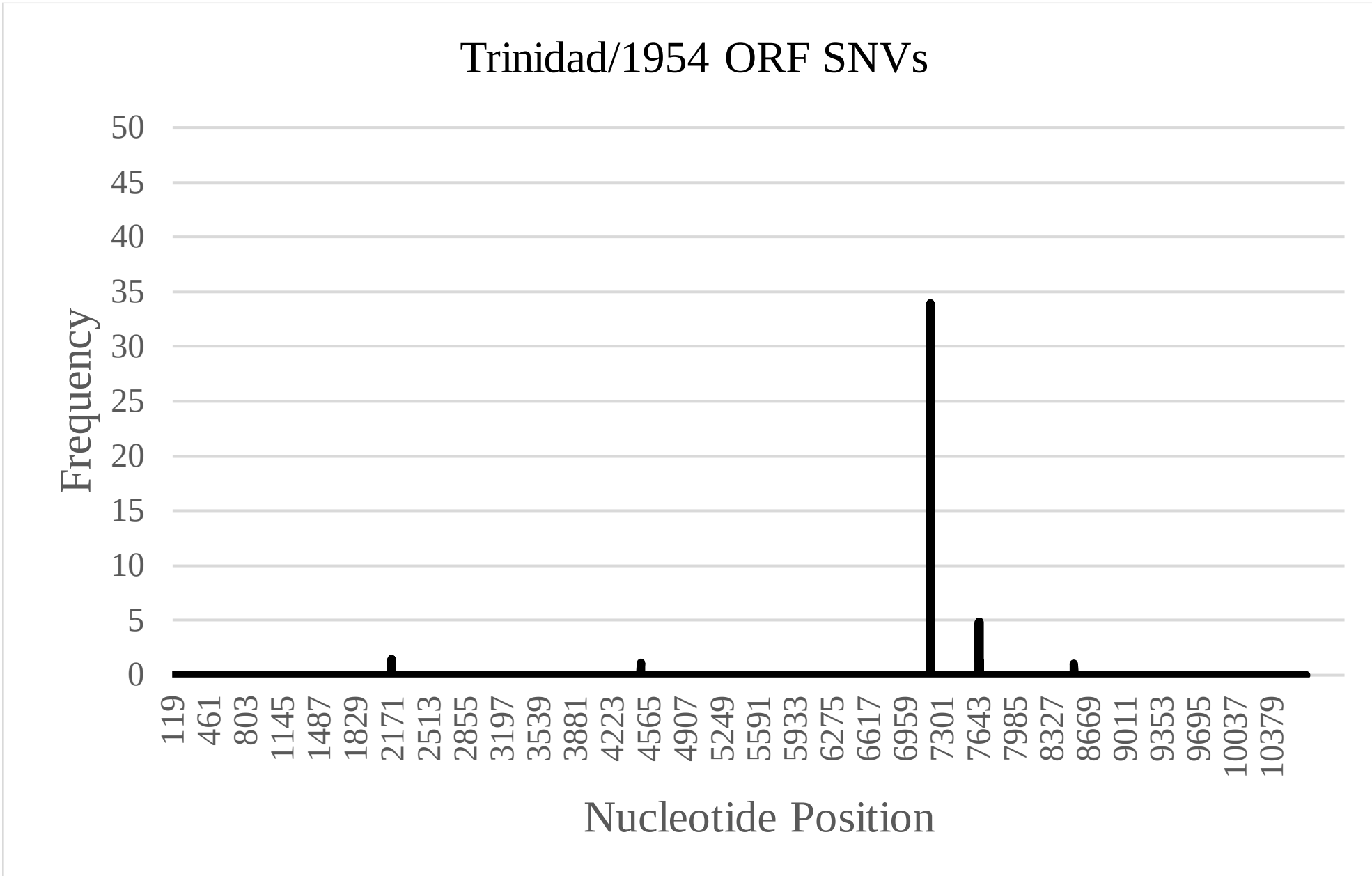
Trinidad/1954 ORF SNVs
Frequency
50
45
40
35
30
25
20
15
10
5
0
119
461
803
1145
1487
1829
2171
2513
2855
3197
3539
3881
4223
4565
4907
5249
5591
5933
6275
6617
6959
7301
7643
7985
8327
8669
9011
9353
9695
10037
10379
Nucleotide Position

F)

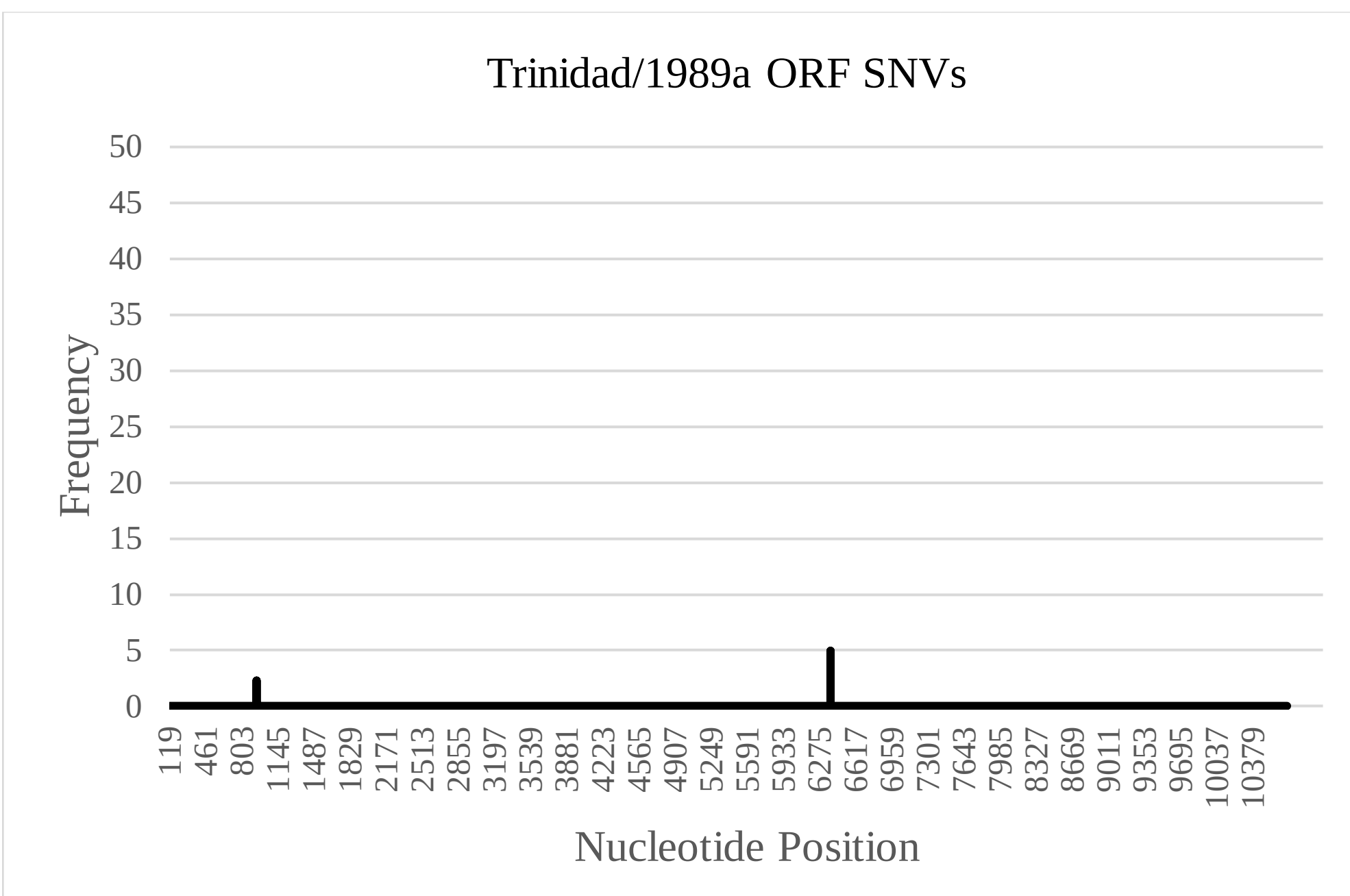
Trinidad/1989a ORF SNVs
Frequency
50
45
40
35
30
25
20
15
10
5
0
119
461
803
1145
1487
1829
2171
2513
2855
3197
3539
3881
4223
4565
4907
5249
5591
5933
6275
6617
6959
7301
7643
7985
8327
8669
9011
9353
9695
10037
10379
Nucleotide Position

G)

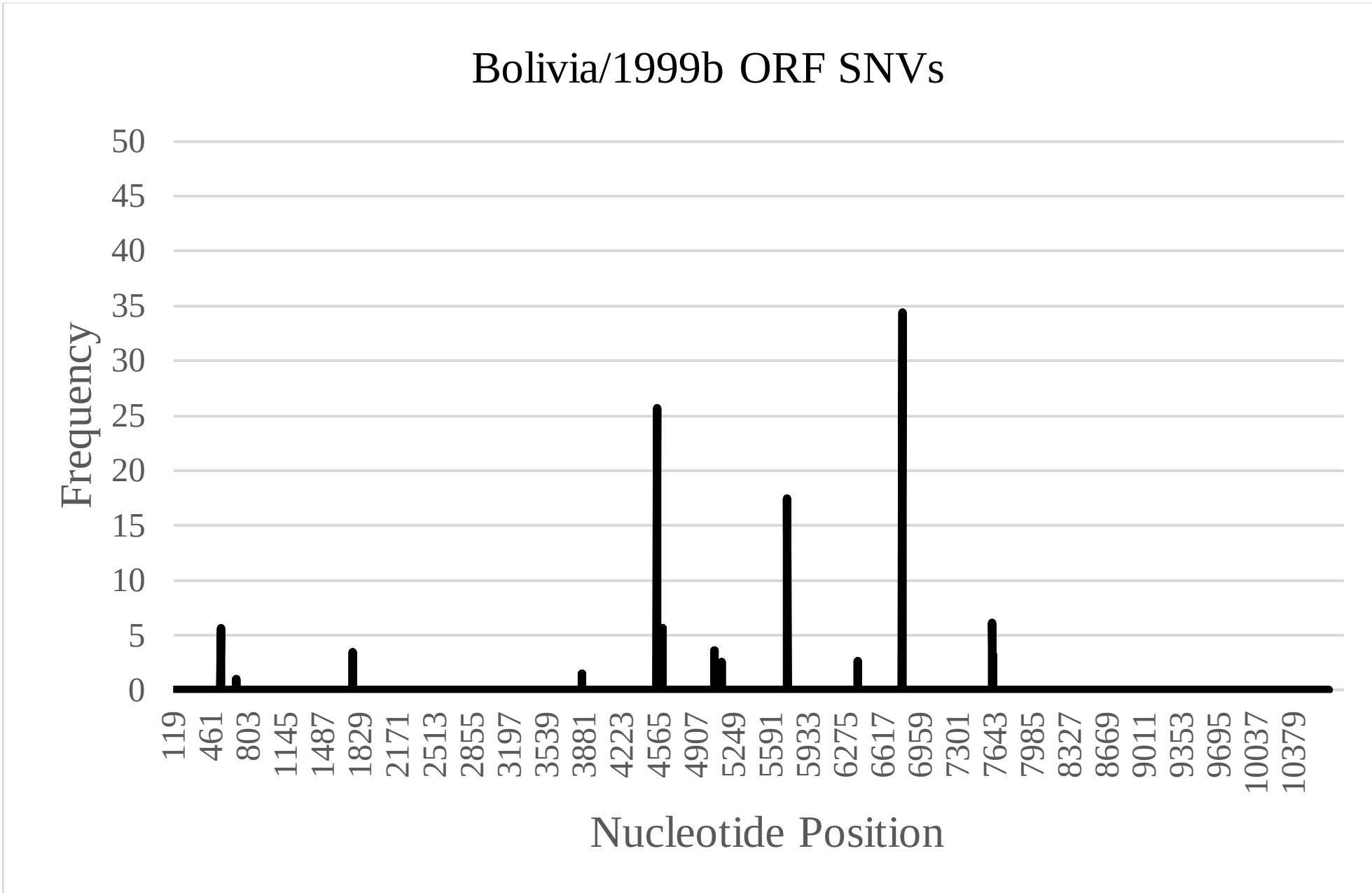
Bolivia/1999b ORF SNVs
50
45
40
35
30
25
20
15
10
5
0
Frequency
119
461
803
1145
1487
1829
2171
2513
2855
3197
3539
3881
4223
4565
4907
5249
5591
5933
6275
6617
6959
7301
7643
7985
8327
8669
9011
9353
9695
10037
10379
Nucleotide Position

H)

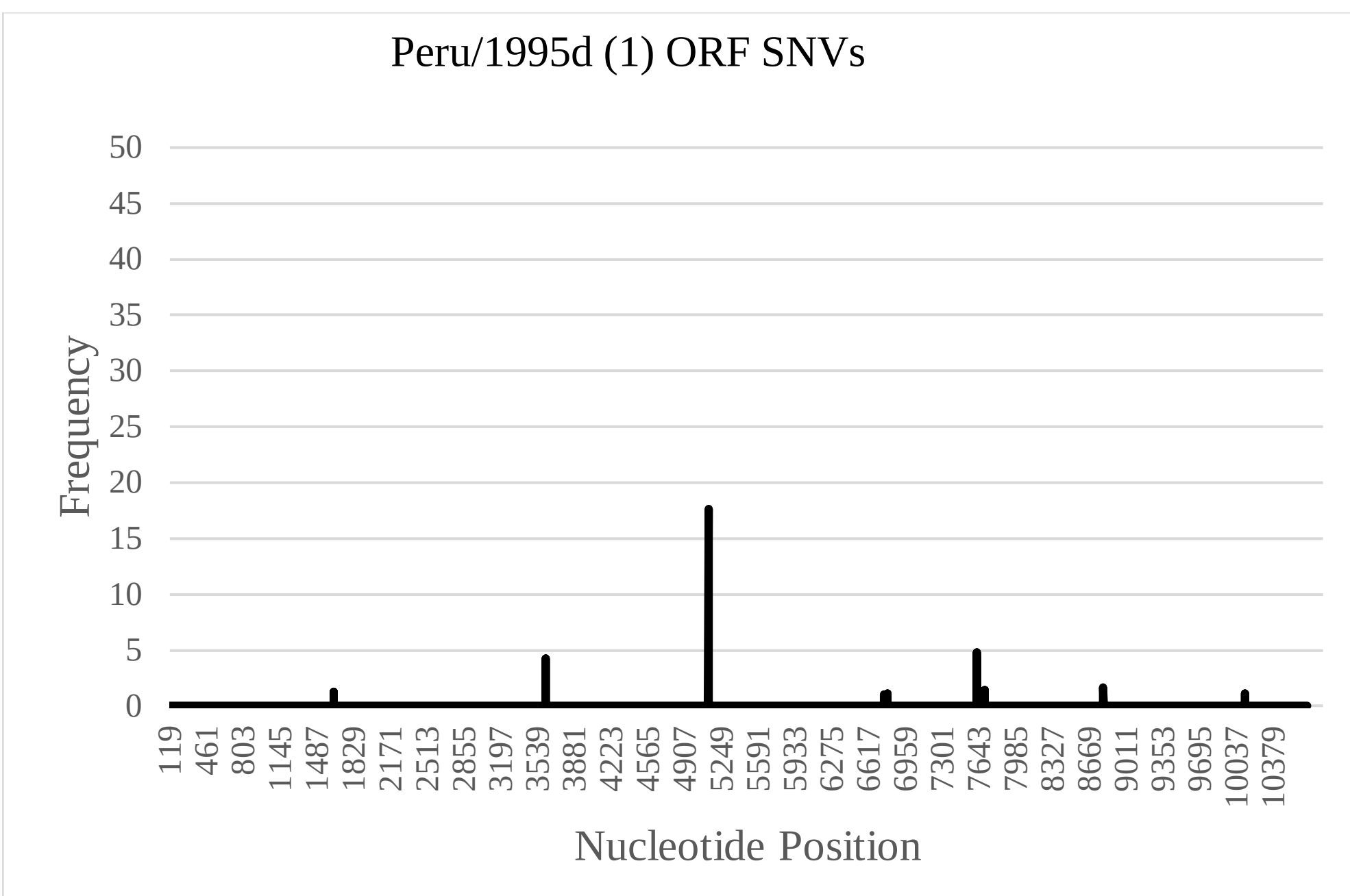
Peru/1995d (1) ORF SNVs
50
45
40
35
30
25
20
15
10
5
0
Frequency
119
461
803
1145
1487
1829
2171
2513
2855
3197
3539
3881
4223
4565
4907
5249
5591
5933
6275
6617
6959
7301
7643
7985
8327
8669
9011
9353
9695
10037
10379
Nucleotide Position

I)

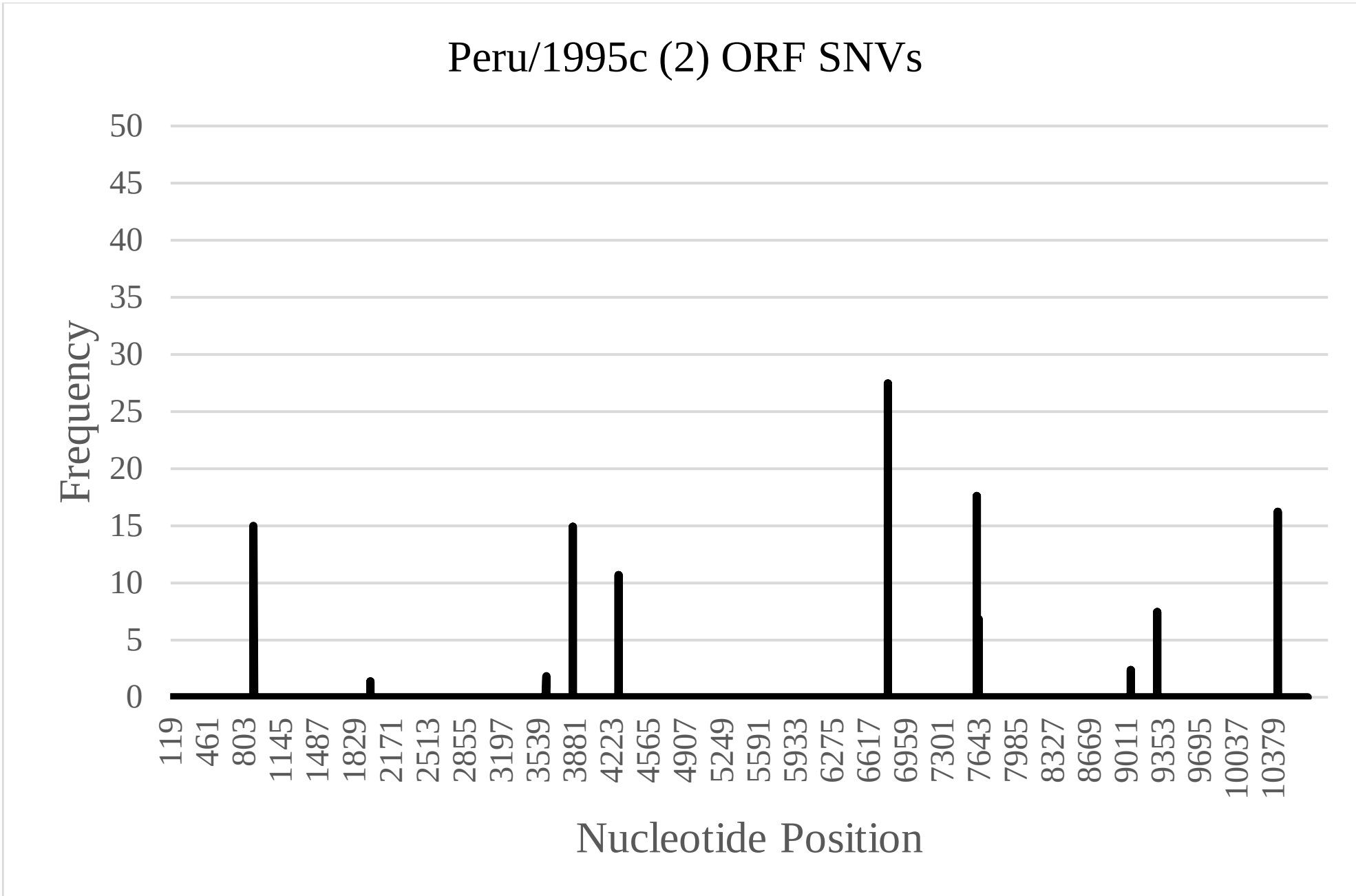

Peru/1995c (2) ORF SNVs
50
45
40
35
30
25
20
15
10
5
0
Frequency
119
461
803
1145
1487
1829
2171
2513
2855
3197
3539
3881
4223
4565
4907
5249
5591
5933
6275
6617
6959
7301
7643
7985
8327
8669
9011
9353
9695
10037
10379
Nucleotide Position

J)

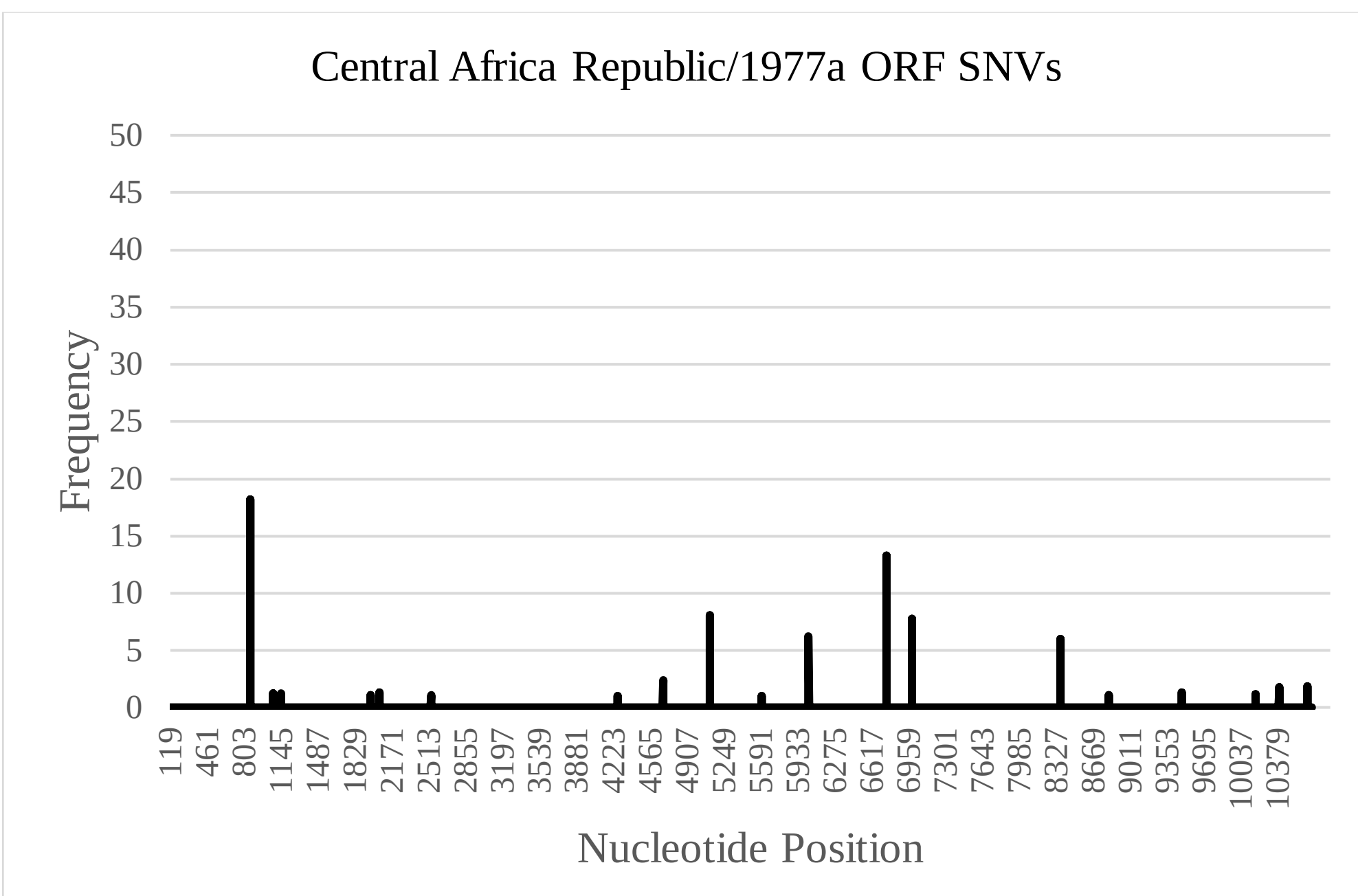

Central Africa Republic/1977a ORF SNVs
50
45
40
35
30
25
20
15
10
5
0
Frequency
119
461
803
1145
1487
1829
2171
2513
2855
3197
3539
3881
4223
4565
4907
5249
5591
5933
6275
6617
6959
7301
7643
7985
8327
8669
9011
9353
9695
10037
10379
Nucleotide Position

K)

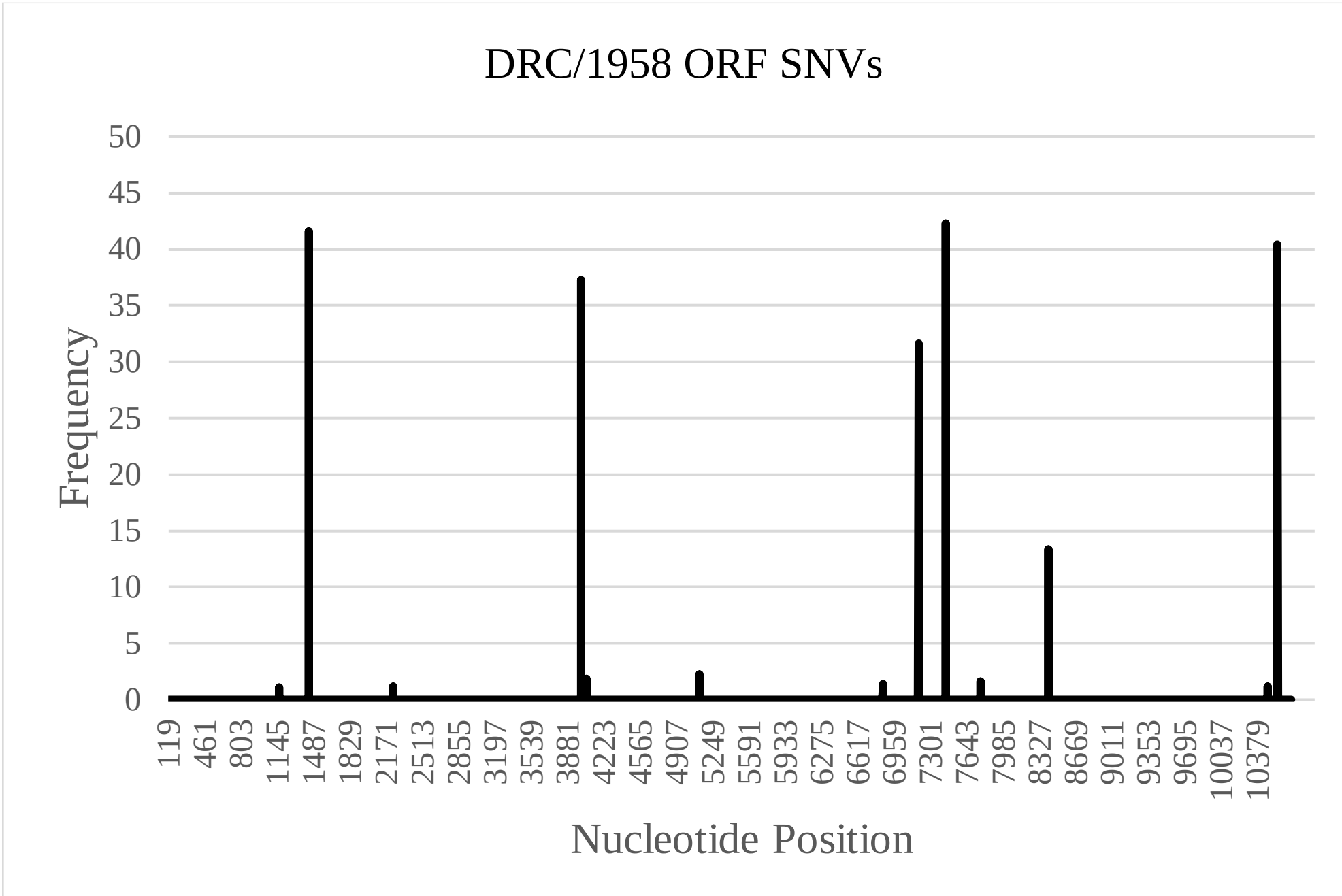
DRC/1958 ORF SNVs
Frequency
50
45
40
35
30
25
20
15
10
5
0
119
461
803
1145
1487
1829
2171
2513
2855
3197
3539
3881
4223
4565
4907
5249
5591
5933
6275
6617
6959
7301
7643
7985
8327
8669
9011
9353
9695
10037
10379
Nucleotide Position

L)

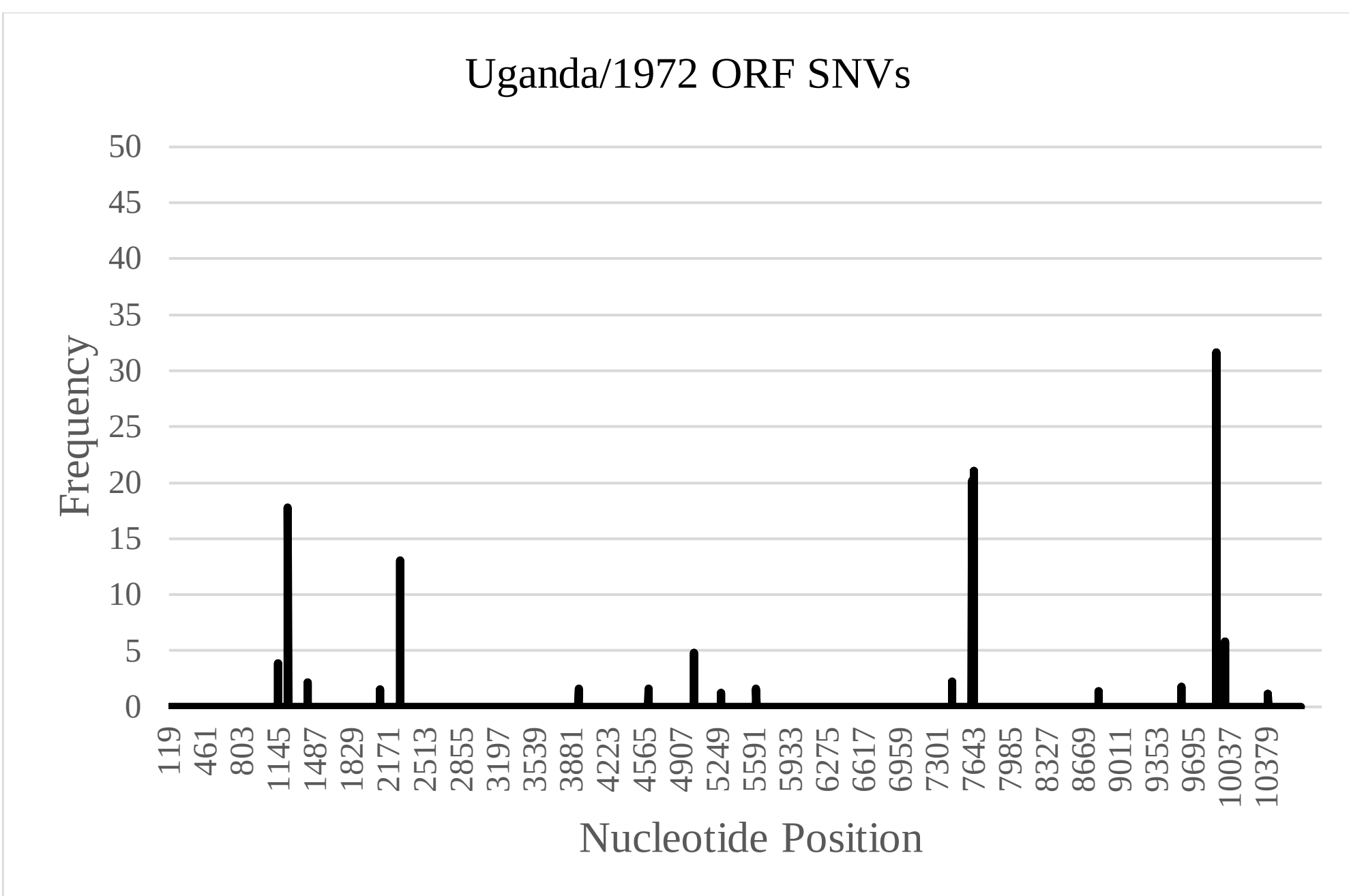
Uganda/1972 ORF SNVs
Frequency
50
45
40
35
30
25
20
15
10
5
0
119
461
803
1145
1487
1829
2171
2513
2855
3197
3539
3881
4223
4565
4907
5249
5591
5933
6275
6617
6959
7301
7643
7985
8327
8669
9011
9353
9695
10037
10379
Nucleotide Position

M)

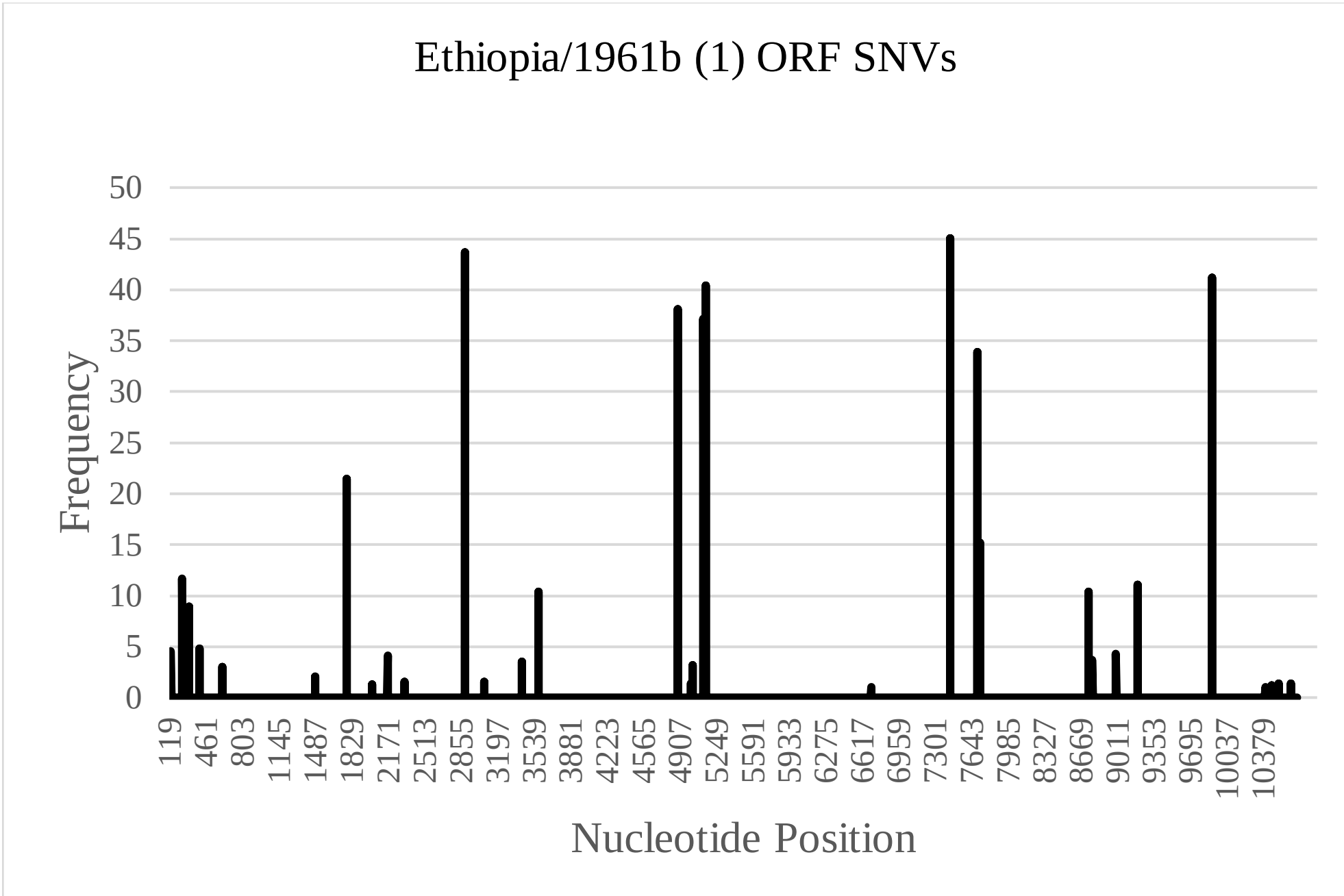
Ethiopia/1961b (1) ORF SNVs
Frequency
50
45
40
35
30
25
20
15
10
5
0
119
461
803
1145
1487
1829
2171
2513
2855
3197
3539
3881
4223
4565
4907
5249
5591
5933
6275
6617
6959
7301
7643
7985
8327
8669
9011
9353
9695
10037
10379
Nucleotide Position

N)

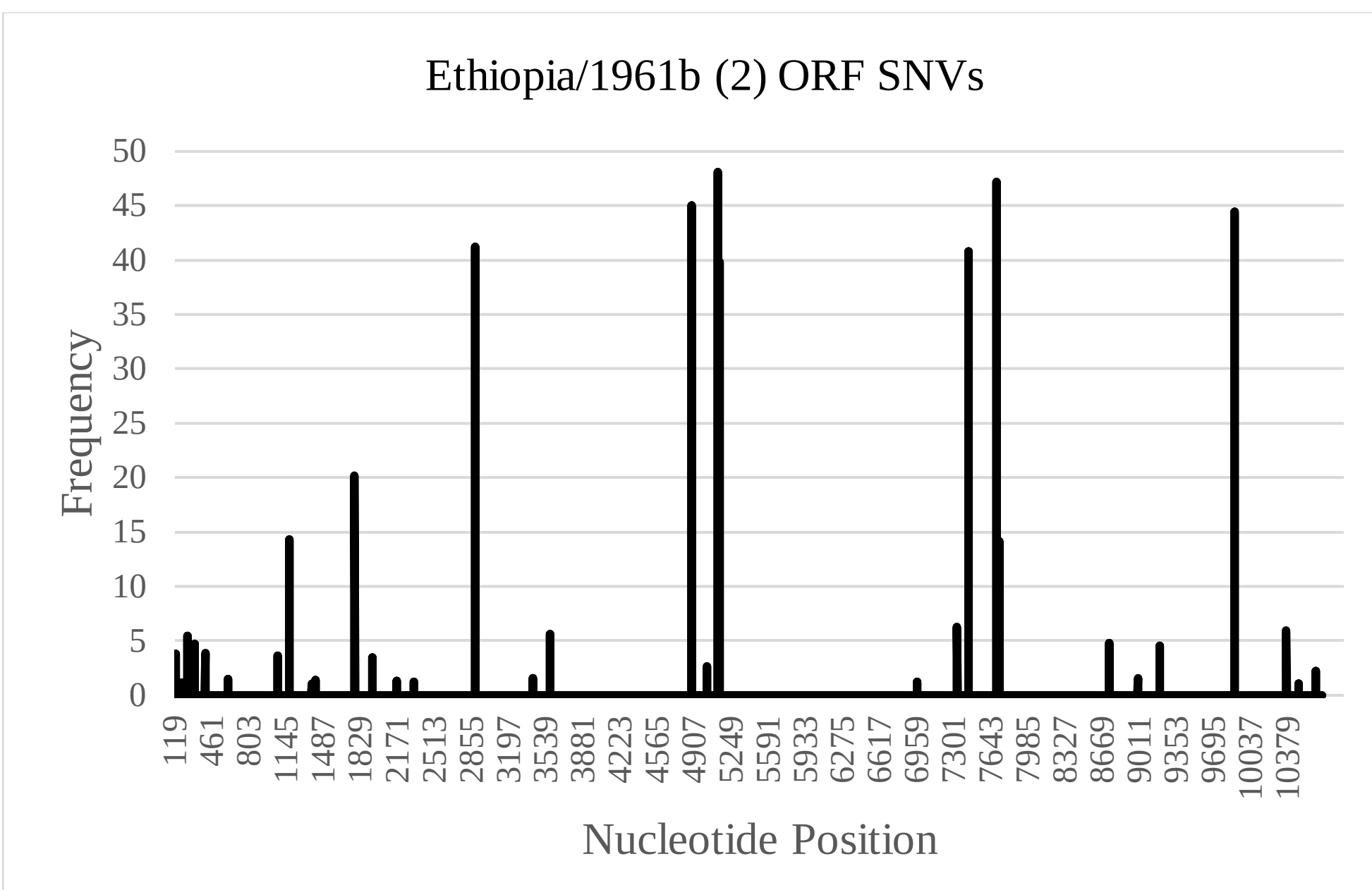
Ethiopia/1961b (2) ORF SNVs
Frequency
50
45
40
35
30
25
20
15
10
5
0
119
461
803
1145
1487
1829
2171
2513
2855
3197
3539
3881
4223
4565
4907
5249
5591
5933
6275
6617
6959
7301
7643
7985
8327
8669
9011
9353
9695
10037
10379
Nucleotide Position

O)

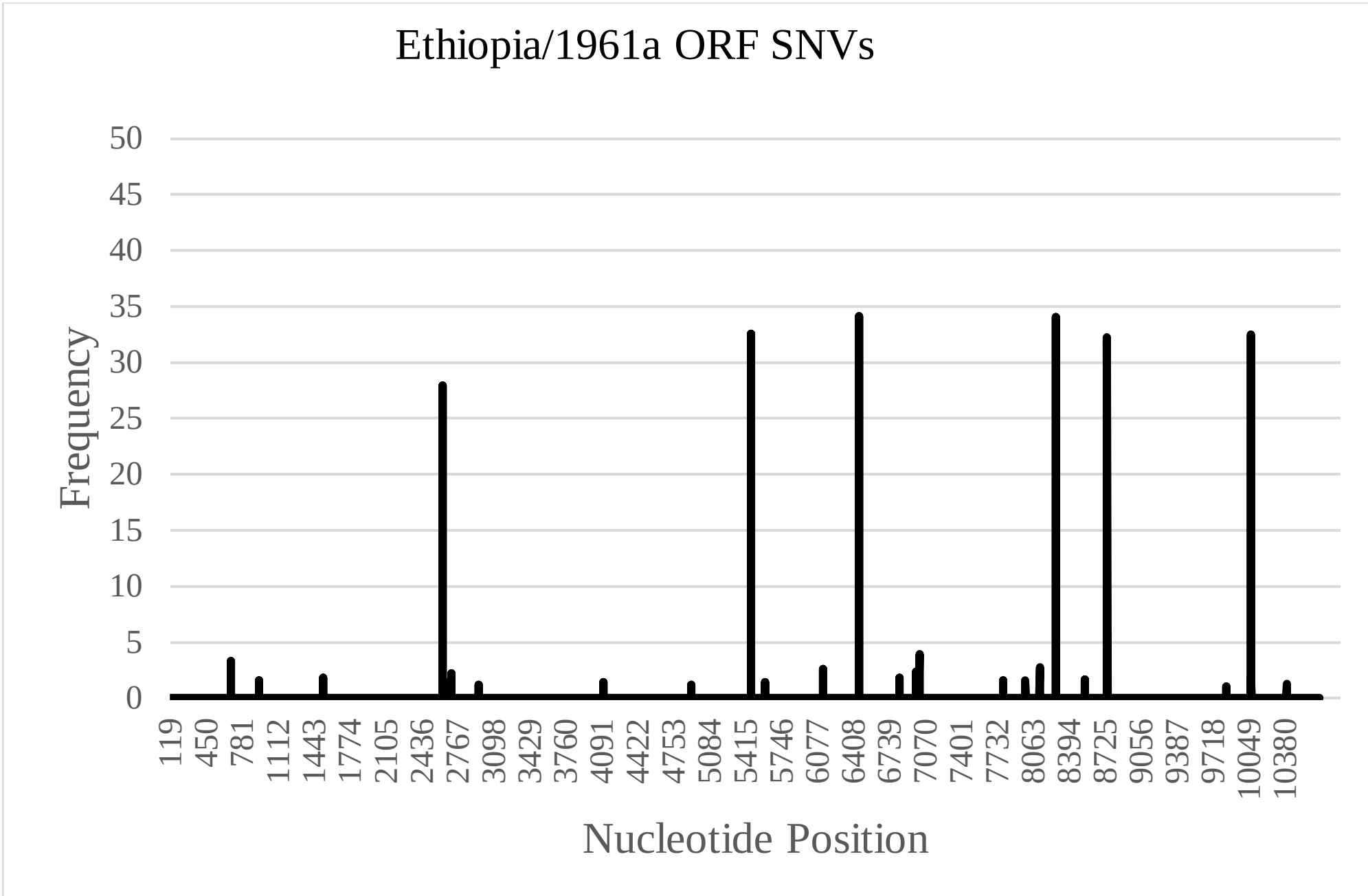

Ethiopia/1961a ORF SNVs
Frequency
50
45
40
35
30
25
20
15
10
5
0
119
450
781
1112
1443
1774
2105
2436
2767
3098
3429
3760
4091
4422
4753
5084
5415
5746
6077
6408
6739
7070
7401
7732
8063
8394
8725
9056
9387
9718
10049
10380
Nucleotide Position

P)

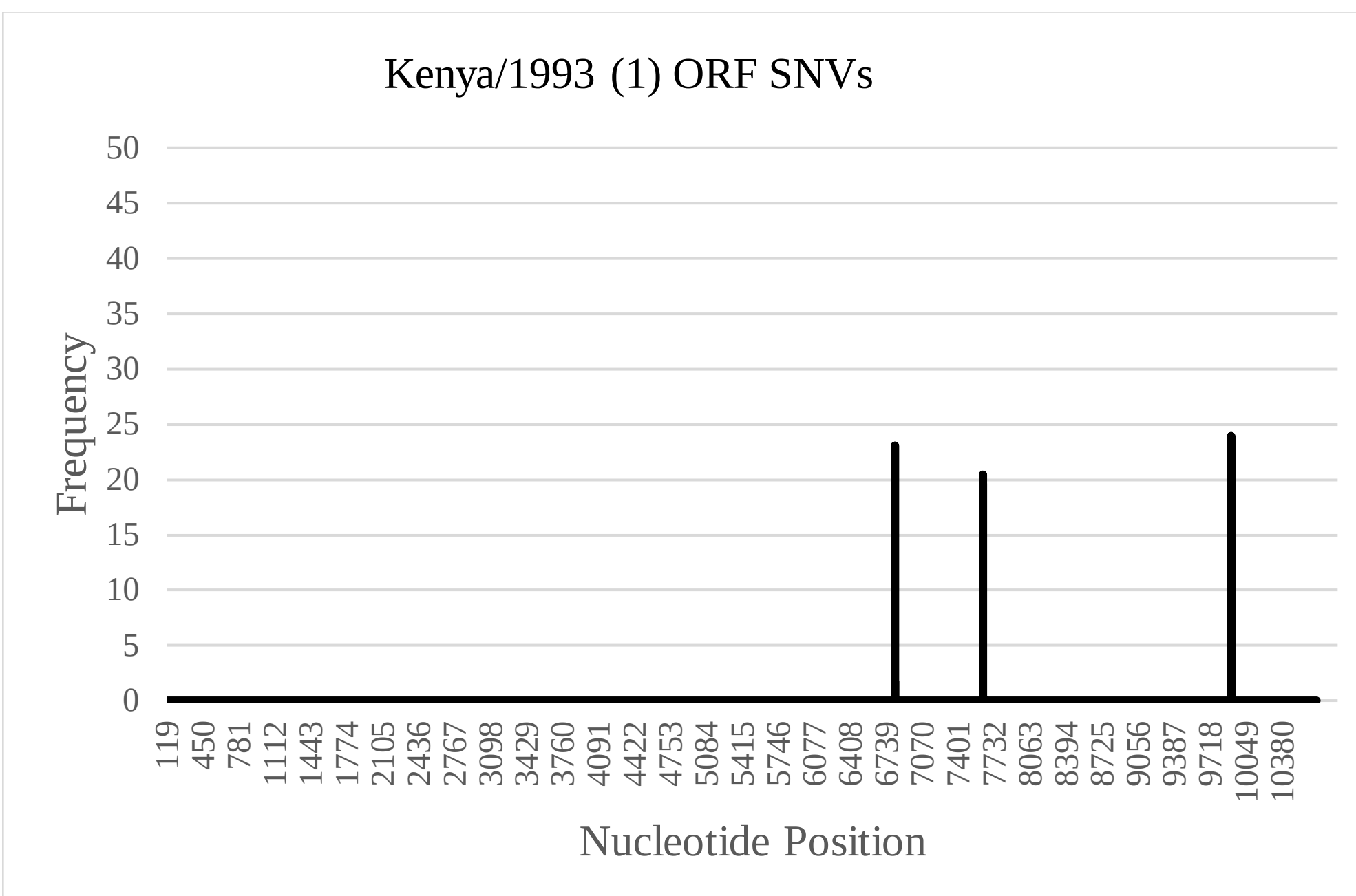

Kenya/1993 (1) ORF SNVs
Frequency
50
45
40
35
30
25
20
15
10
5
0
119
450
781
1112
1443
1774
2105
2436
2767
3098
3429
3760
4091
4422
4753
5084
5415
5746
6077
6408
6739
7070
7401
7732
8063
8394
8725
9056
9387
9718
10049
10380
Nucleotide Position

Q)

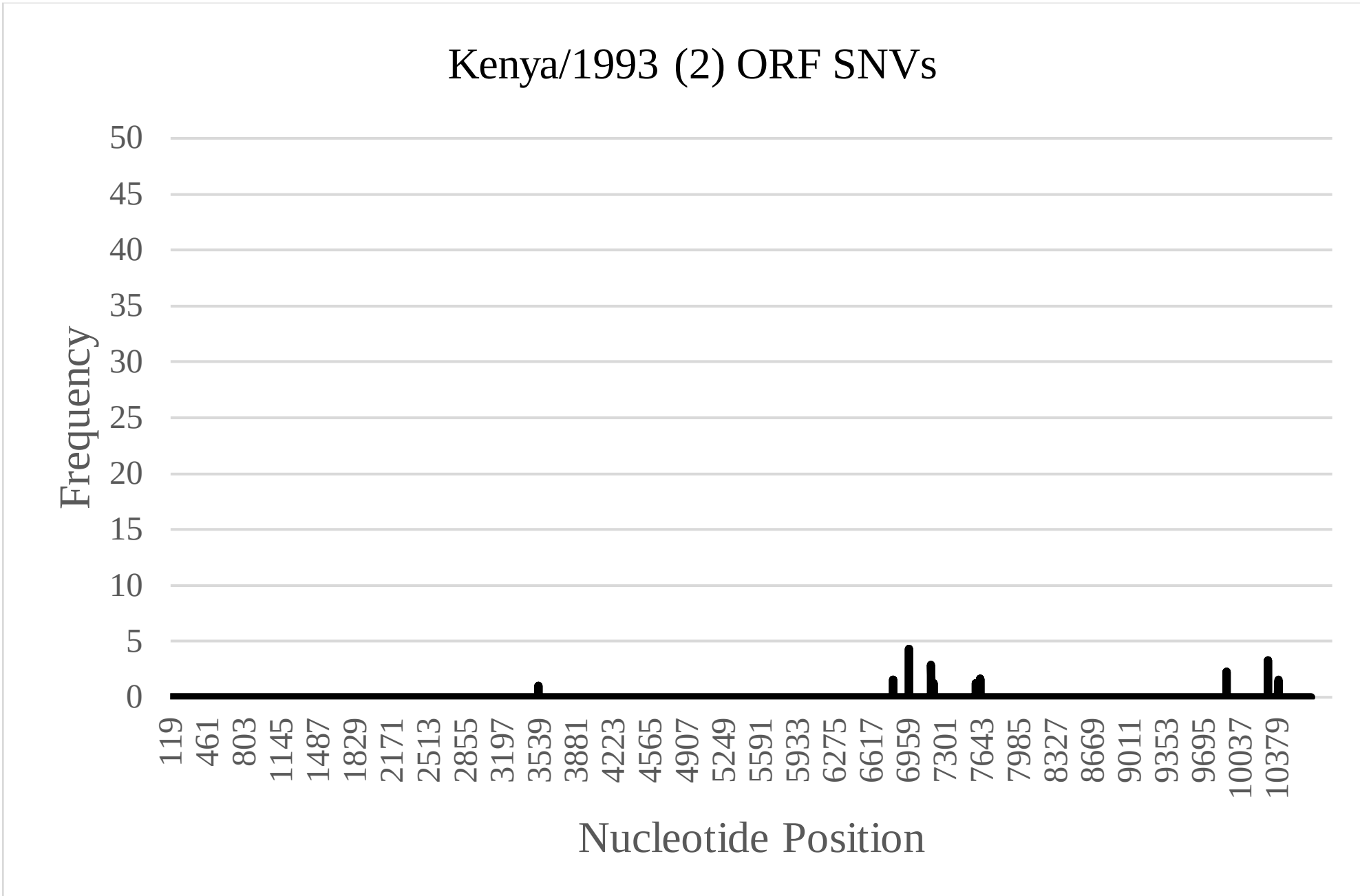

Kenya/1993 (2) ORF SNVs
Frequency
50
45
40
35
30
25
20
15
10
5
0
119
461
803
1145
1487
1829
2171
2513
2855
3197
3539
3881
4223
4565
4907
5249
5591
5933
6275
6617
6959
7301
7643
7985
8327
8669
9011
9353
9695
10037
10379
Nucleotide Position

R)

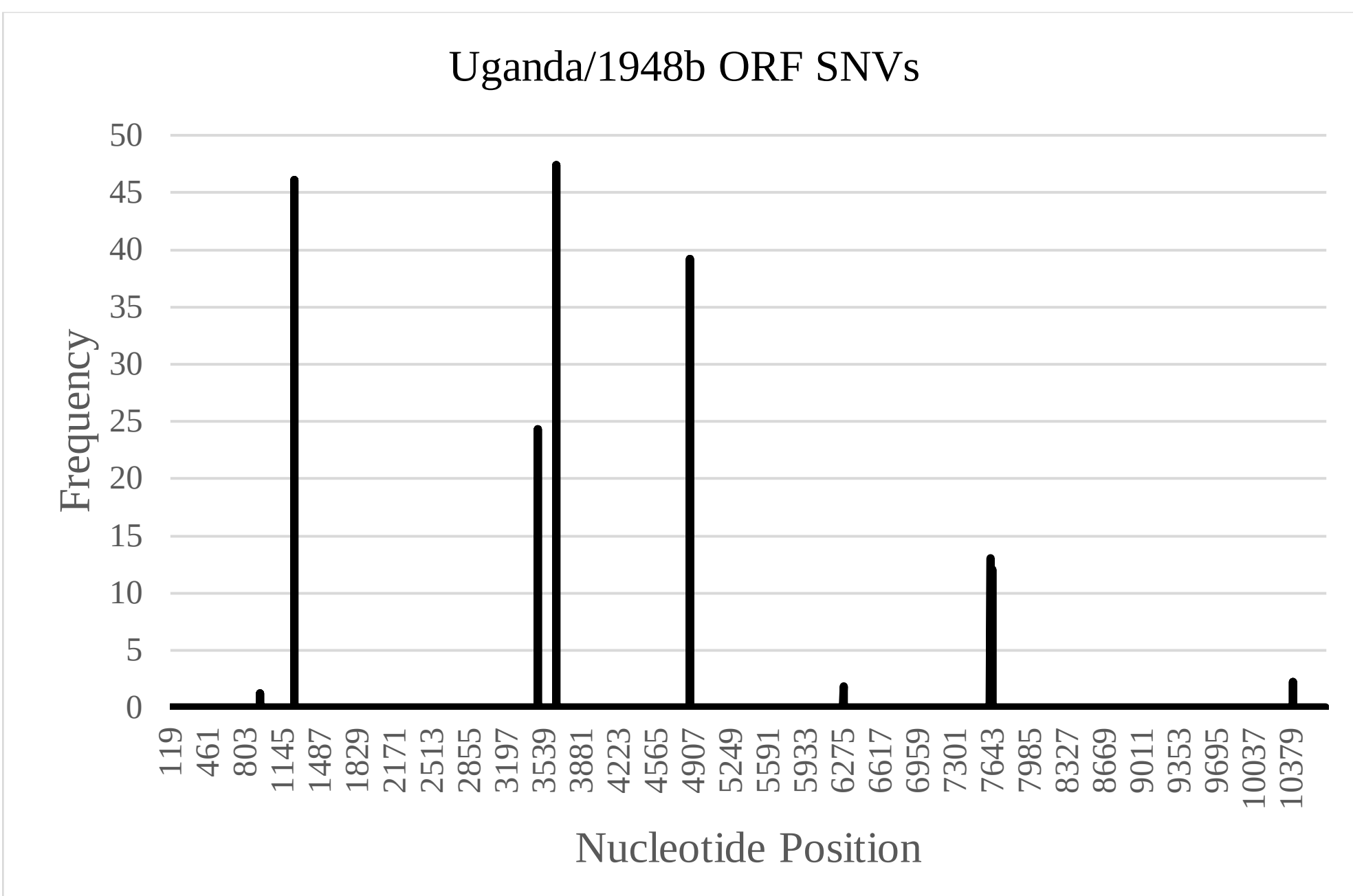

Uganda/1948b ORF SNVs
Frequency
50
45
40
35
30
25
20
15
10
5
0
119
461
803
1145
1487
1829
2171
2513
2855
3197
3539
3881
4223
4565
4907
5249
5591
5933
6275
6617
6959
7301
7643
7985
8327
8669
9011
9353
9695
10037
10379
Nucleotide Position

S)

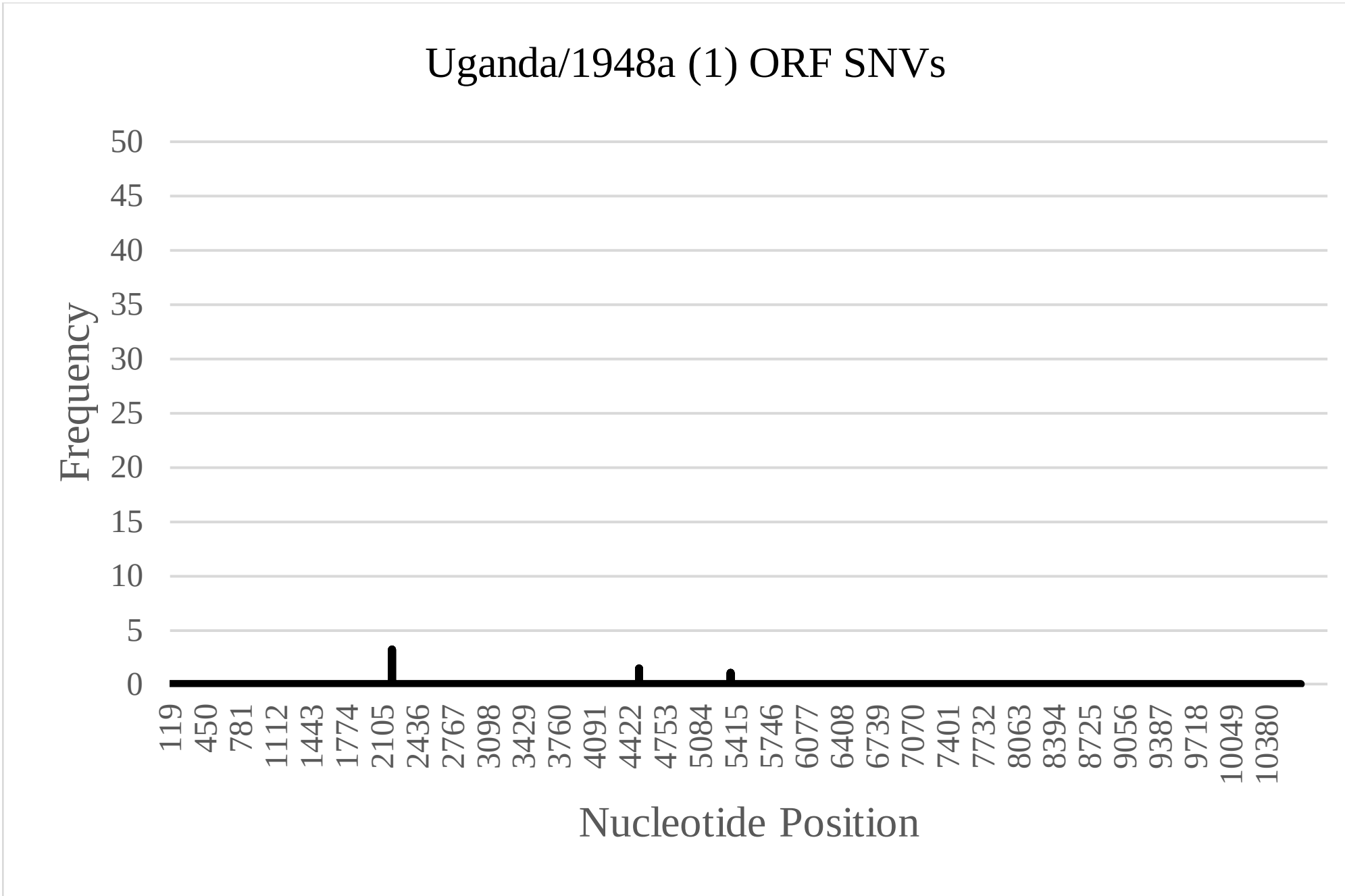
Uganda/1948a (1) ORF SNVs
Frequency
50
45
40
35
30
25
20
15
10
5
0
119
450
781
1112
1443
1774
2105
2436
2767
3098
3429
3760
4091
4422
4753
5084
5415
5746
6077
6408
6739
7070
7401
7732
8063
8394
8725
9056
9387
9718
10049
10380
Nucleotide Position

T)

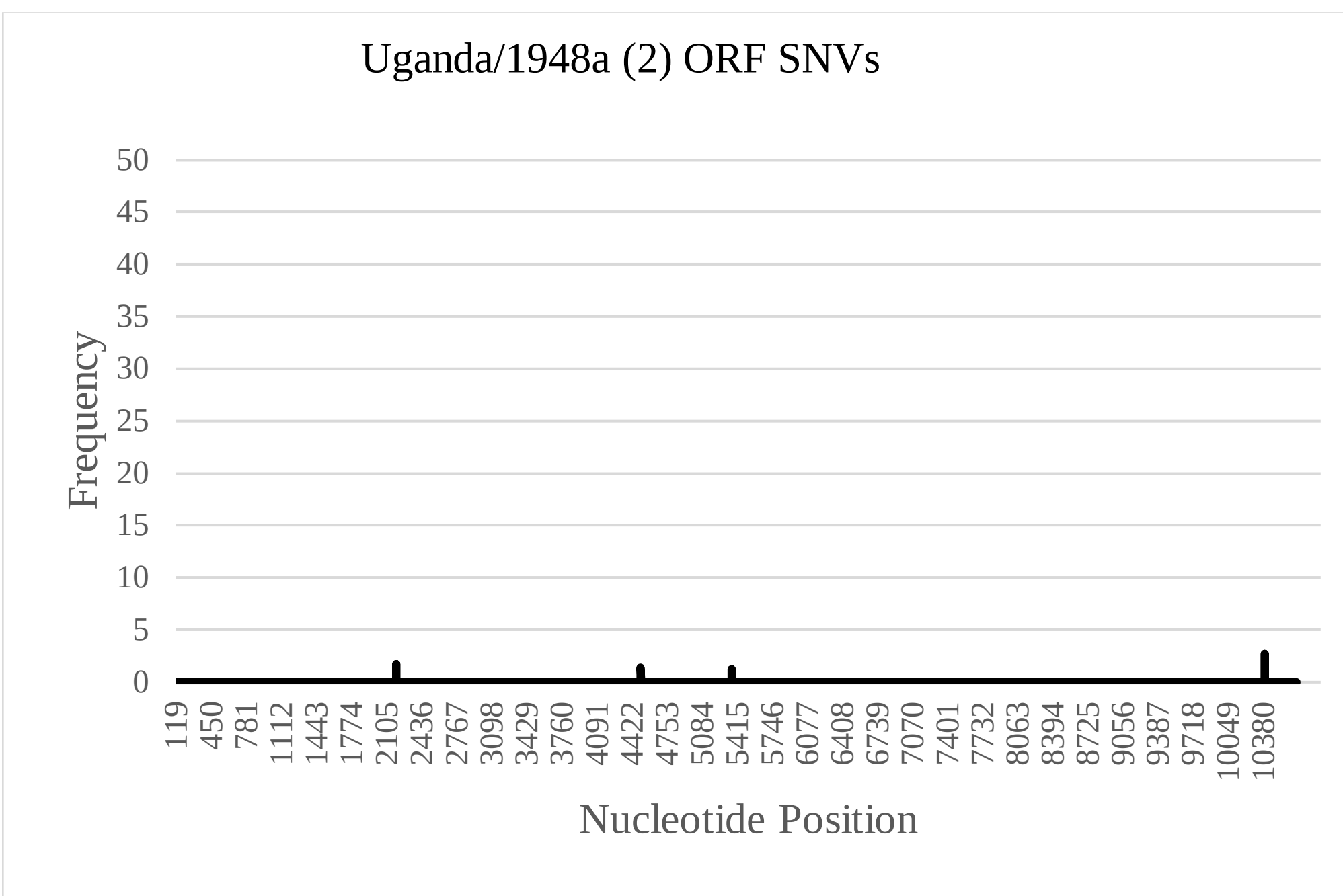
Uganda/1948a (2) ORF SNVs
Frequency
50
45
40
35
30
25
20
15
10
5
0
119
450
781
1112
1443
1774
2105
2436
2767
3098
3429
3760
4091
4422
4753
5084
5415
5746
6077
6408
6739
7070
7401
7732
8063
8394
8725
9056
9387
9718
10049
10380
Nucleotide Position

U)

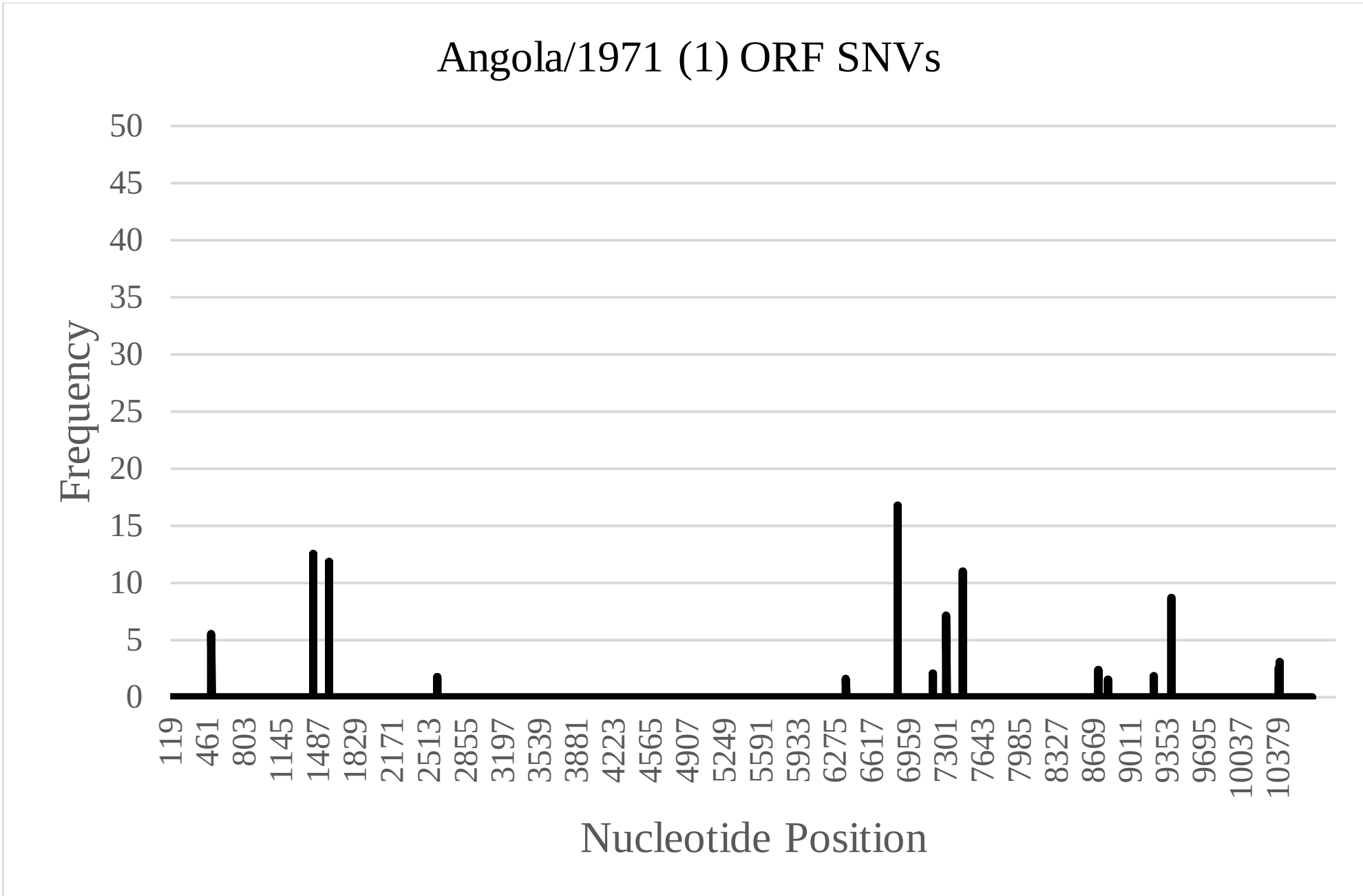
Angola/1971 (1) ORF SNVs
Frequency
50
45
40
35
30
25
20
15
10
5
0
119
461
803
1145
1487
1829
2171
2513
2855
3197
3539
3881
4223
4565
4907
5249
5591
5933
6275
6617
6959
7301
7643
7985
8327
8669
9011
9353
9695
10037
10379
Nucleotide Position

V)

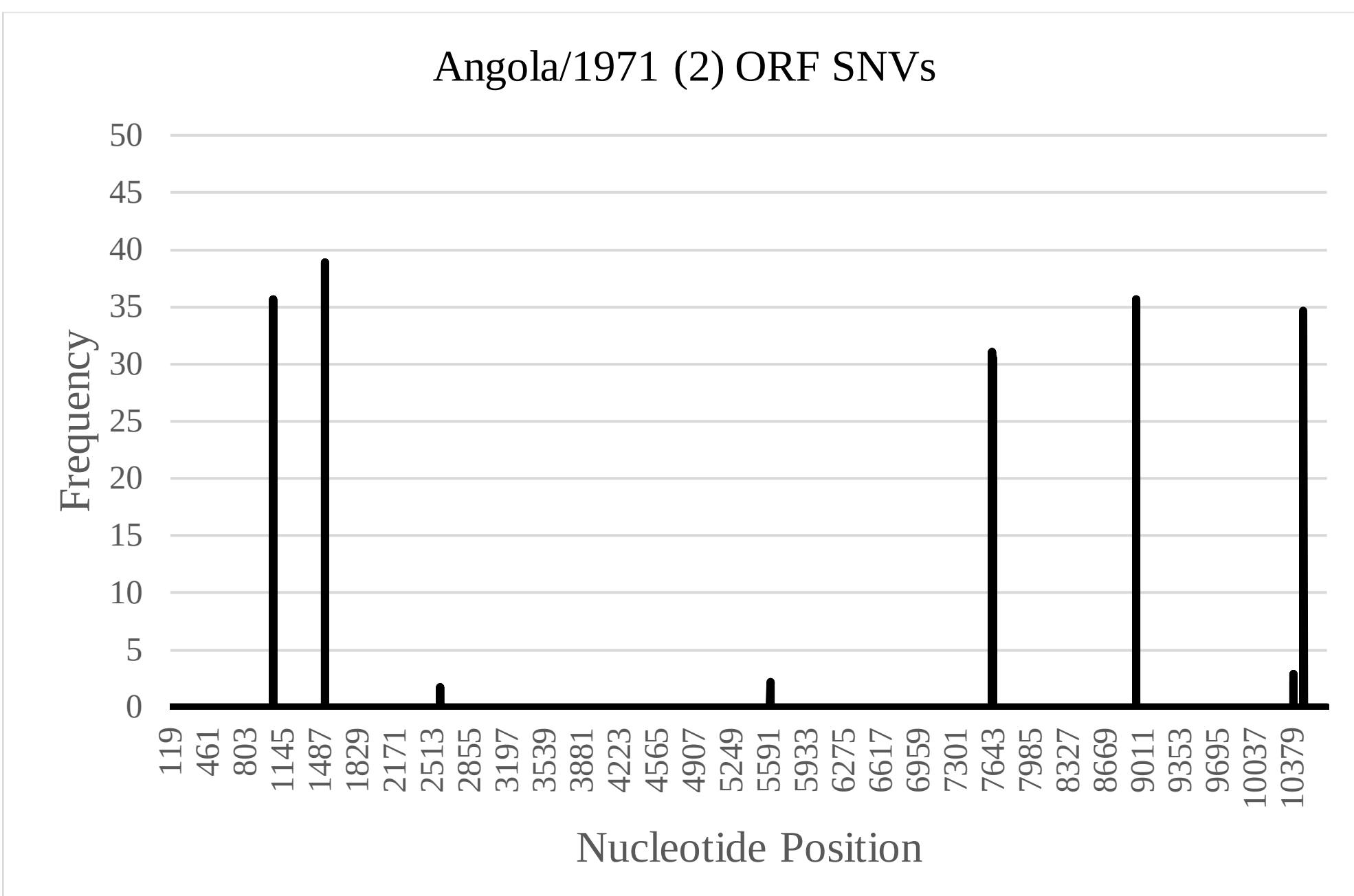
Angola/1971 (2) ORF SNVs
Frequency
50
45
40
35
30
25
20
15
10
5
0
119
461
803
1145
1487
1829
2171
2513
2855
3197
3539
3881
4223
4565
4907
5249
5591
5933
6275
6617
6959
7301
7643
7985
8327
8669
9011
9353
9695
10037
10379
Nucleotide Position

W)

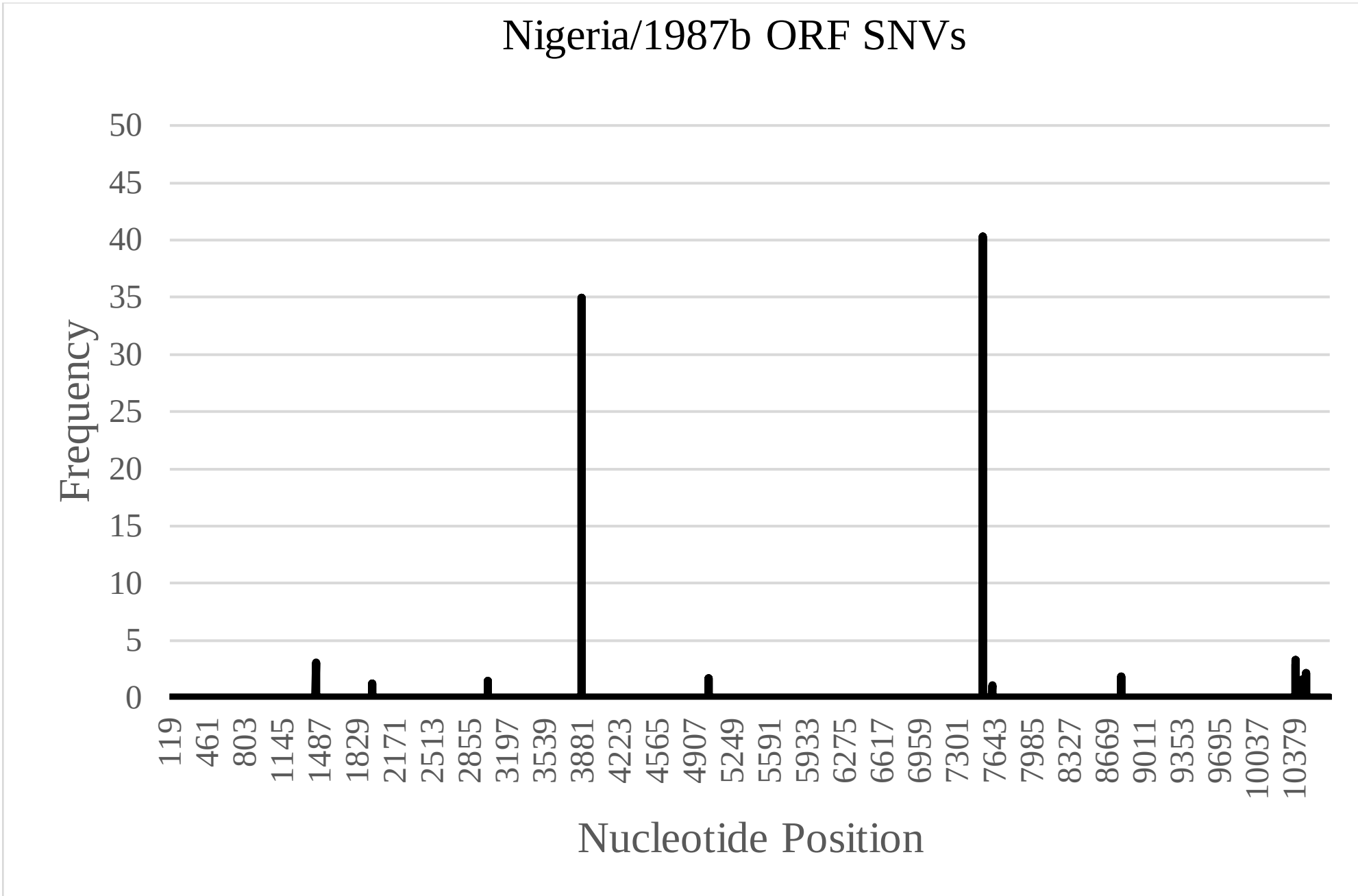
Nigeria/1987b ORF SNVs
Frequency
50
45
40
35
30
25
20
15
10
5
0
119
461
803
1145
1487
1829
2171
2513
2855
3197
3539
3881
4223
4565
4907
5249
5591
5933
6275
6617
6959
7301
7643
7985
8327
8669
9011
9353
9695
10037
10379
Nucleotide Position

X)

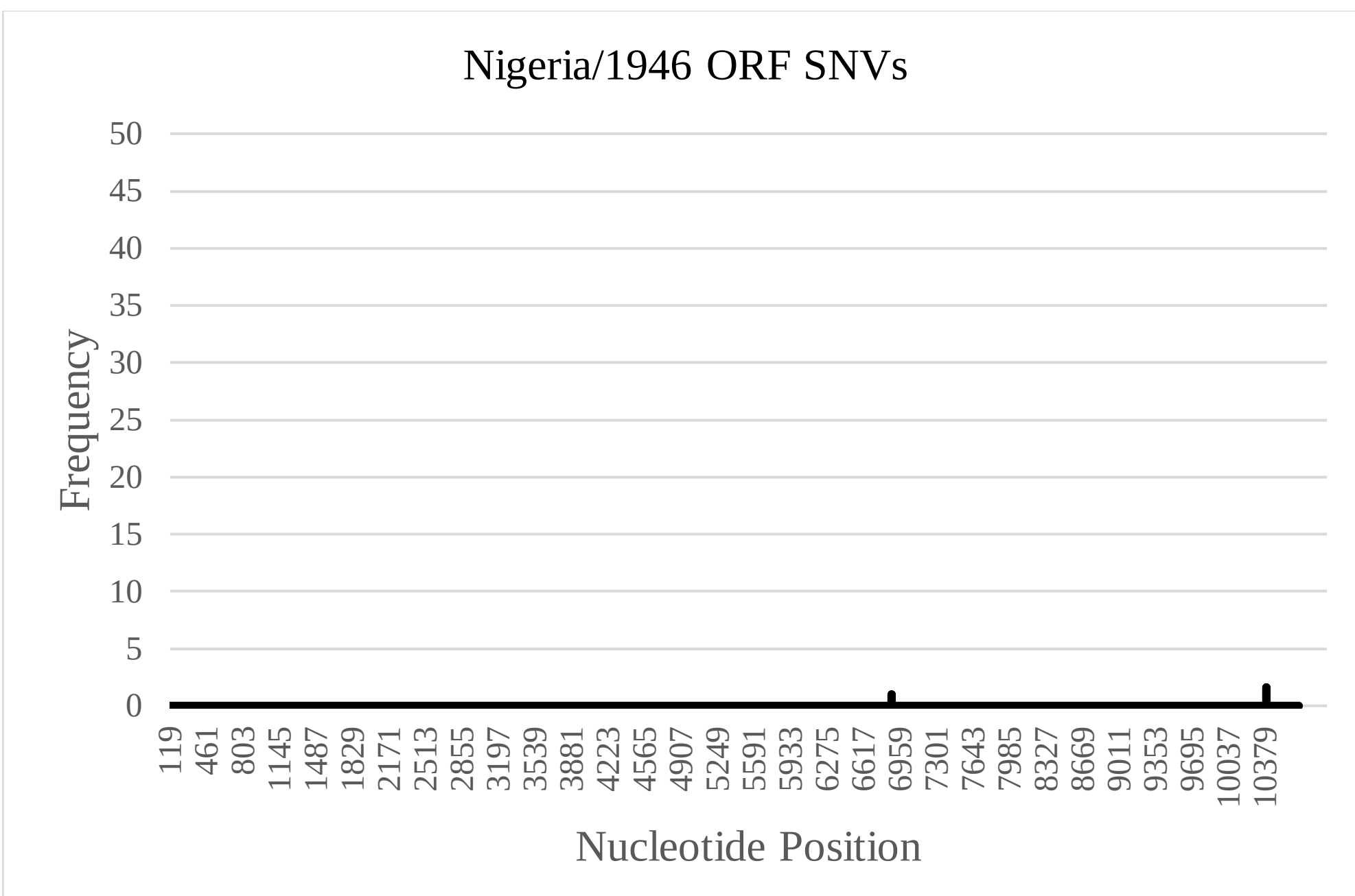
Nigeria/1946 ORF SNVs
Frequency
50
45
40
35
30
25
20
15
10
5
0
119
461
803
1145
1487
1829
2171
2513
2855
3197
3539
3881
4223
4565
4907
5249
5591
5933
6275
6617
6959
7301
7643
7985
8327
8669
9011
9353
9695
10037
10379
Nucleotide Position

Y)

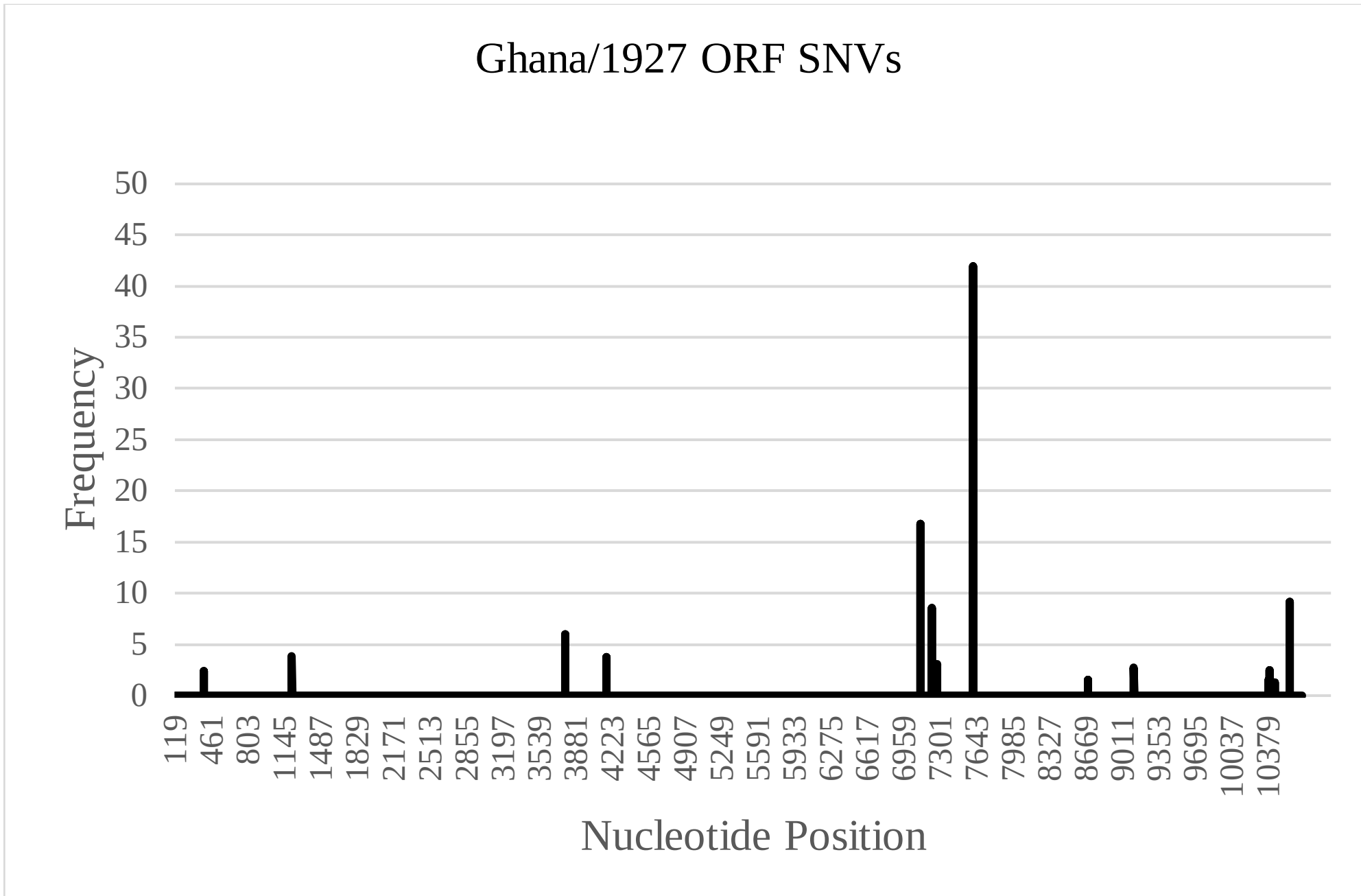

Ghana/1927 ORF SNVs
50
45
40
35
30
25
20
15
10
5
0
Frequency
119
461
803
1145
1487
1829
2171
2513
2855
3197
3539
3881
4223
4565
4907
5249
5591
5933
6275
6617
6959
7301
7643
7985
8327
8669
9011
9353
9695
10037
10379
Nucleotide Position

Supplementary Table 1: SE, SNV, and passage histories of viral strains with multiple preparations.

Genotype	Strain	Taxon	Total Passage History	Shannon Entropy	SNVs > 1.00	SNVs > 5.00%	SNVs > 10.00%	Coding SNVs
**Angola**	14FA	Angola/1971	SM 7, Mosq. 1, Vero 4	0.0032	15	7	4	2
	14FA	Angola/1971	SM 7, Mosq. 1, Vero 1	0.0035	10	6	6	2
**South America I**	1337	Ecuador/1979	Vero 1	0.0039	5	0	0	0
	1337	Ecuador/1979	SM 2, Vero 1	0.0032	7	0	0	0
**East Africa**	BC7914	Kenya/1993	SM 1, C6/36 1, Vero 1	0.0029	5	4	4	3
	BC7914	Kenya/1993	SM 1, C6/36 1, Vero 3	0.0026	13	1	0	0
	A709-4-A2	Uganda/1948a	C6/36, Vero 1	0.0031	3	0	0	0
	A709-4-A2	Uganda/1948a	C6/36, Vero 2	0.0025	4	0	0	0
**East and Central Africa**	Couma	Ethiopia/1961b	SM 46, Mosq. 2, Vero 1	0.0045	35	14	13	12
	Couma	Ethiopia/1961b	SM 46, Mosq. 1, Vero 3	0.005	33	14	10	10

Supplementary Table 2: ANOVA for substitution per site values of each genotype

ANOVA

Source of Variation	SS	df	MS	F	P-value	F crit
Between Groups	0.0036	6	0.0006	65535	X	X
Within Groups	0	0	65535			
Total	0.003558994	6				

Supplementary Table 3: ANOVA for SE values of each genotype

ANOVA

Source of Variation	SS	df	MS	F	P-value	F crit
Between Groups	2.61933E-06	6	4.366E-07	2.3806	0.0850	2.8477
Within Groups	2.56733E-06	14	1.834E-07			
Total	5.18667E-06	20				

Supplementary Table 4: ANOVA for SNVs >1.00% values of each genotype

ANOVA

Source of Variation	SS	df	MS	F	P-value	F crit
Between Groups	894	6	149	4	0.0099	3
Within Groups	466	14	33			
Total	1361	20				

Supplementary Table 5: TUKEYHSD post-hoc test results for SNVs >1.00% values of each genotype

Comparison	Abs. Mean Diff	Q Critical Value	Signifcant?
East and Central Africa vs East Africa	17	15	Yes
East and Central Africa vs West Africa I	16	14	Yes

Supplementary Table 6: ANOVA for SNVs >5.00% values of each genotype

ANOVA

Source of Variation	SS	df	MS	F	P-value	F crit
Between Groups	136	6	23	2	0.2034	3
Within Groups	191	14	14			
Total	327	20				

Supplementary Table 7: ANOVA for SNVs >10.00% values of each genotype

ANOVA

Source of Variation	SS	df	MS	F	P-value	F crit
Between Groups	136	6	23	2	0.2034	3
Within Groups	191	14	14			
Total	327	20				

Supplementary Table 8: ANOVA for Coding SNVs values of each genotype

ANOVA

Source of Variation	SS	df	MS	F	P-value	F crit
Between Groups	88	6	15	2	0.2197	3
Within Groups	128	14	9			
Total	215	20				
